# Thielavins W–Z_7_, New Antifouling Thielavins from the Marine-Derived Fungus *Thielavia* sp. UST030930-004

**DOI:** 10.3390/md15050128

**Published:** 2017-04-29

**Authors:** Zhuang Han, Yong-Xin Li, Ling-Li Liu, Liang Lu, Xian-Rong Guo, Xi-Xiang Zhang, Xiao-Yong Zhang, Shu-Hua Qi, Ying Xu, Pei-Yuan Qian

**Affiliations:** 1 Institute of Deep-sea Science and Engineering, Chinese Academy of Sciences, 28 Luhuitou Road, Sanya 572000, China; zhuanghan@idsse.ac.cn; 2 Division of Life Science, The Hong Kong University of Science and Technology, Clear Water Bay, Hong Kong, China; liyongxin@connect.ust.hk (Y.-X.L.); leonie@nwsuaf.edu.cn (L.-L.L.); luliangust@163.com (L.L.); 3 Imaging & Characterization Core lab, King Abdullah University of Science and Technology, Thuwal 23955-6900, Saudi Arabia; xianrong.guo@kaust.edu.sa; 4 Physical Science and Engineering, King Abdullah University of Science and Technology, Thuwal 23955-6900, Saudi Arabia; xixiang.zhang@kaust.edu.sa; 5 Key Laboratory of Marine Bio-resources Sustainable Utilization, South China Sea Institute of Oceanology, Chinese Academy of Sciences, 164 West Xingang Road, Guangzhou 510301, China; zhangxiaoyong@scsio.ac.cn (X.-Y.Z.); shuhuaqi@scsio.ac.cn (S.-H.Q.); 6 College of Life Science, Shenzhen University, 3688 Nanhai Ave, Shenzhen 518060, China

**Keywords:** marine-derived fungus, *Thielavia* sp., antifouling, *Balanus* (=*Amphibalanus*) *amphitrite*, thielavins

## Abstract

Eleven new depsides—thielavins W–Z (**1**–**4**) and thielavins Z_1_–Z_7_ (**5**–**11**)—and also four known thielavins—A, H, J, and K (**12**–**15**)—were isolated from the ethyl acetate extract of a marine-derived fungal strain *Thielavia* sp UST030930-004. All of these compounds were evaluated for antifouling activity against cyprids of the barnacle *Balanus* (=*Amphibalanus*) *amphitrite*. The results showed that compounds **1**–**3** and **6**–**13** were active, with EC_50_ values ranging from 2.95 ± 0.59 to 69.19 ± 9.51 μM, respectively. The inhibitive effect of compounds **1**–**3** and **7** was reversible. This is the first description of the antifouling activity of thielavins against barnacle cyprids.

## 1. Introduction

Marine microorganisms are being explored as potentially important sources of environmentally friendly antifouling (AF) compounds because they can be cultivated to produce diverse chemical compounds under optimal culture conditions [1]. Several potent antifouling compounds have been isolated from various microorganisms, including polyethers from the marine bacterium *Winogradskyella Poriferorum* [2], 12-methyltetradecanoid acid and butenolides from *Streptomyces* sp. UST040711-290 and *Streptomyces Albidoflavus* [3,4], 3-chloro-2,5-dihydroxybenzyl alcohol from the marine fungus *Ampelomyces* sp. [5], and Diindol-3-ylmethanes from *Pseudovibrio Denitrificans* [6]. Of these compounds, butenolides are regarded as the most potent natural antifoulants [7]. The binding targets of the butenolide 5-octylfuran-2(5H)-one have been identified in the barnacle *Balanus* (=*Amphibalanus*) *amphitrite*, the bryozoan *Bugula neritina* and the marine bacterium *Vibrio* sp. UST020129-010 [8]. These studies clearly demonstrate that marine microbes are a promising source of effective antifouling compounds.

**Figure 1:**
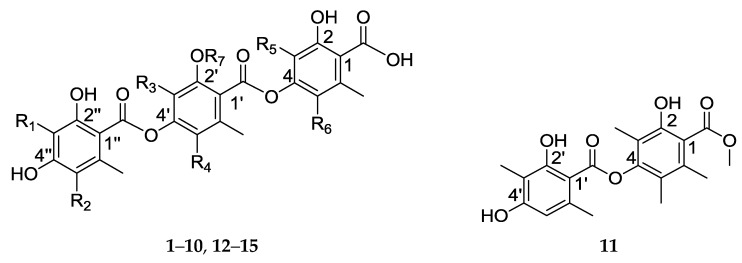
Structures of compounds **1**–**15**.

In the present study, we report 11 new depsides—thielavins W–Z, Z_1_–Z_7_ (**1**–**11**)—and four known thielavins (Figure 1)—thielavins A, H, J, and K (**12**–**15**) [9]—which we isolated from the marine-derived fungal strain *Thielavia* sp. UST030930-004, and we evaluated the antifouling activity of each against the barnacle *B. amphitrite* cyprids.

## 2. Results and Discussion

### 2.1. Identification of the Fungus

The internal transcribed spacer (ITS) sequence of the fungal isolate UST030930-004 (GenBank accession number KJ716558) had a similarity of 97% with *Thielavia terrestris* NRRL 8126 (CP003011), indicating that isolate UST030930-004 is a *Thielavia* sp. closely related to this taxon.

### 2.2. Structure Elucidation

Fifteen compounds were isolated from the ethyl acetate extract of *Thielavia* sp. UST030930-004, including the 11 new compounds—thielavins W–Z (**1**–**4**) and thielavins Z_1_–Z_7_ (**5**–**11**)—together with 4 known compounds—thielavins A, H, J, and K (**12**–**15**). Their chemical structures were determined using MS, 1D- and 2D-NMR spectroscopy.

**Figure 2:**
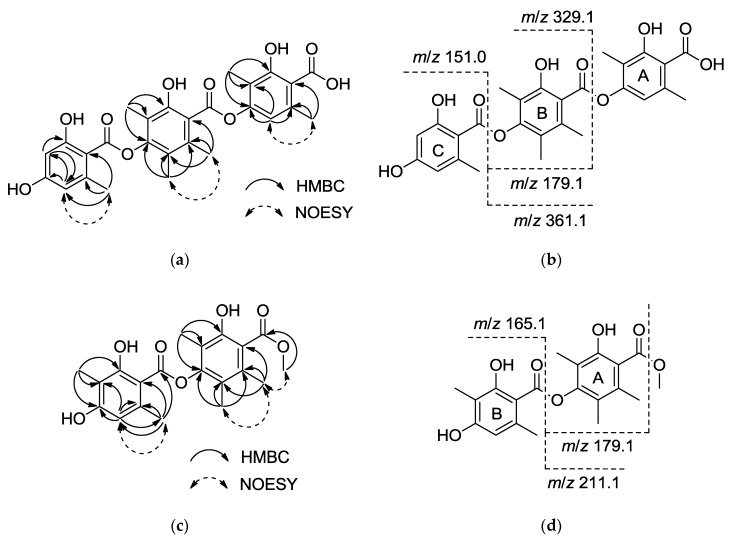
(**a**) Key HMBC and NOESY correlations; (**b**) ESIMS in-source fragmentation of compound **1**; (**c**) Key HMBC and NOESY correlations; (**d**) ESIMS in-source fragmentation of compound **11**.

Compound **1** was isolated as a white amorphous powder. The positive HRESIMS provided [M + H]^+^ at *m*/*z* 511.1604, corresponding to a molecular formula of C_27_H_26_O_10_ (calcd. for C_27_H_27_O_10_ [M + H]^+^ 511.1599), and the UV spectrum revealed absorption at *λ*_max_ 218.1, 267.8, and 304.2 nm. The ^1^H, ^13^C-NMR (Table 1) and HSQC spectra of **1** indicated the presence of 6 methyl groups at *δ*_H_ 2.52 (3H, s), 2.40 (3H, s), 2.29 (3H, s), 2.06 (3H, s), 2.06 (3H, s), 1.99 (3H, s), 18 olefinic carbons, including fifteen quaternary carbons at *δ*_C_ 138.7, 165.4, 114.2, 149.5, 114.7, 131.8, 150.7, 116.5, 149.1, 120.8, 121.6, 141.0, 161.4, 160.8, and 107.6, and 3 tertiary carbons at *δ*_C_ 110.3, 100.6, 110.3. Three carbonyl signals were also observed at *δ*_C_ 172.0, 167.1, and 166.7. These data suggested that the compound is similar to thielavin H (13) [9]. The only difference between **1** and thielavin H was that one methyl group in thielavin H was absent in **1**. The structure of **1** was established based on HMBC correlations and in-source collision-induced dissociation (ISCID) fragment ions. The HMBC correlations observed from the methyl group *δ*_H_ 1.99 (3-Me) to *δ*_C_ 165.4 (C-2), 114.2 (C-3) and 149.5 (C-4), from *δ*_H_ 2.52 (6-Me) to *δ*_C_ 138.7 (C-1), 110.3 (C-5) and 114.7 (C-6), and from an olefinic proton *δ*_H_ 6.18 (H-5) to C-4 and C-6 indicated the presence of a 2,4-dioxygenated-3,6-dimethylbenzene substructure (A). The second substructure (B) was confirmed by long-range couplings from *δ*_H_ 2.06 (3′-Me) to *δ*_C_ 150.7 (C-2′), 116.5 (C-3′) and 149.1(C-4′), and from *δ*_H_ 2.06 (5′-Me) to *δ*_C_ 120.8 (C-5′), 121.6 (C-6′) and C-4′, and couplings from another methyl group *δ*_H_ 2.29 (6′-Me) to *δ*_C_ 131.8 (C-1′), C-5′ and C-6′. The HMBC correlations from *δ*_H_ 2.40 (6″-Me) to *δ*_C_ 141.0 (C-1″), 110.3 (C-5″) and 107.6 (C-6″), correlations from an olefinic proton *δ*_H_ 6.26 (H-3″) to *δ*_C_ 161.4 (C-2″), C-5″, and correlations from olefinic proton *δ*_H_ 6.26 (H-5″) to *δ*_C_ 100.6 (C-3″), 160.8 (C-4″) and C-6″, indicated the presence of a 2,4-dioxygenated-6-methylbenzene substructure (C). From the deduction above, the remaining three carbonyl groups can only be assigned to C-1, C-1′ and C-1″, and these carbonyl groups may be interchangeable as no HMBC correlations were observed (Figure 2a). The positive ISCID MS/MS gave ion peaks at *m*/*z* 329.1, 151.0, 361.1 and 179.1, suggesting the absence of [M − A]^+^, [M − A − B]^+^, [M − C + 2H]^+^ and [M − A − C + 2H]^+^ fragments (Figure 2b), indicating that the order of the substructures is A − B − C (Figure 2b). Thus the structure of **1** was determined (shown in Figure 1) and named thielavin W.

Comparison of the UV, NMR, HRESIMS and ISCID MS/MS data shows that compounds **2**–**10** have the same core structure as compound **1**. Their structures were determined using the procedure described for compound **1**, and named thielavins X–Z and Z_1_–Z_5_, respectively. All of the ^1^H and ^13^C-NMR assignments of the new compounds **2**–**10** are summarized in Table 1, Table 2, Table 3 and Table 4.

Compound **11** was obtained as a white amorphous powder. Positive HRESIMS gave [M + H]^+^ as *m*/*z* 375.1464, and the molecular formula was established as C_20_H_22_O_7_ (calcd. for C_20_H_23_O_7_ [M + H]^+^ 375.1438). The maximum UV absorption occurred at *λ*_max_ 215.8, 276.1, and 308.9 nm, and the ^1^H NMR (Table 3) revealed the presence of 5 quaternary methyl signals [*δ*_H_ 1.96 (3H, s), 1.96 (3H, s), 2.13 (3H, s), 1.97 (3H, s), and 2.54 (3H, s)], 1 methoxy signal [*δ*_H_ 3.83 (3H, s)], and 1 olefinic proton signal [*δ*_H_ 6.40 (1H, s)]. The ^13^C-NMR and HSQC results (Table 3) indicated the presence of 12 olefinic carbons, including 11 quaternary carbons at *δ*_C_ 162.6, 161.0, 150.7, 148.5, 139.4, 132.1, 121.4, 120.1, 116.0, 108.6, and 102.7, 1 tertiary carbon at *δ*_C_ 111.0 and 2 carbonyl groups at *δ*_C_ 168.7 and 168.9. Based on these data, compound **11** was proposed as a thielavin derivative consisting of two hydroxybenzoic acid groups with a methyl ester terminus. HMBC revealed correlations (Figure 2c) between *δ*_H_ 1.96 (3-Me) and *δ*_C_ 150.7 (C-2), 116.0 (C-3) and 148.5 (C-4), between *δ*_H_ 1.96 (s, 5-Me) and *δ*_C_ 120.1 (C-5), 121.4 (C-6), C-4, and between *δ*_H_ 2.13 (6-Me) to *δ*_C_ 132.1 (C-1), C-5 and C-6, and also revealed long range correlations between *δ*_H_ 3.83 (1-COOMe) and *δ*_C_ 168.7 (1-C=O). NOESY results show the correlation from protons 6-Me to methoxy proton (1-COOMe), indicating the presence of a 2-hydroxyl-4-oxygenated-3,5,6-trimethylbenzoic methyl ester subunit, corresponding to the ion fragment of *m*/*z* 179.1. The HMBC correlations (Figure 2c) from *δ*_H_ 1.97 (3′-Me) to *δ*_C_ 162.6 (C-2′), 108.6 (C-3′) and 161.0 (C-4′), from *δ*_H_ 6.40 (H-5′) to *δ*_C_ 102.7 (C-6′), 24.0 (6′-Me), C-3′ and C-4′, and from *δ*_H_ 2.54 (6′-Me) to *δ*_C_ 139.4 (C-1′), 111.0 (C-5′) and C-6′, suggested the presence of a 2-hydroxyl-4-oxygenated-3,6-dimethylbenzoyl subunit, corresponding to an ion fragment with *m*/*z* 211.1 (Figure 2d). These two units should be connected by an ester bond, but there was no correlation between the two units and the connection was only supported by ion fragments [M − B + 2H]^+^, [M – Ome − B + H]^+^ and [M – Ome − A]^+^ at *m*/*z* 211.1, 179.1 and 165.1, respectively. Thus, the structure of 11 was determined (shown in Figure 1**)** and named thielavin Z_7_.

Compounds **12**–**15** were identified as thielavin A (**12**), thielavin H (**13**), thielavin J (**14**), and thielavin K (**15**), by comparison of their spectral data with those reported in the literature [9,10].

### 2.3. Anti-Larval Settlement and Recovery Activities

**Figure 3:**
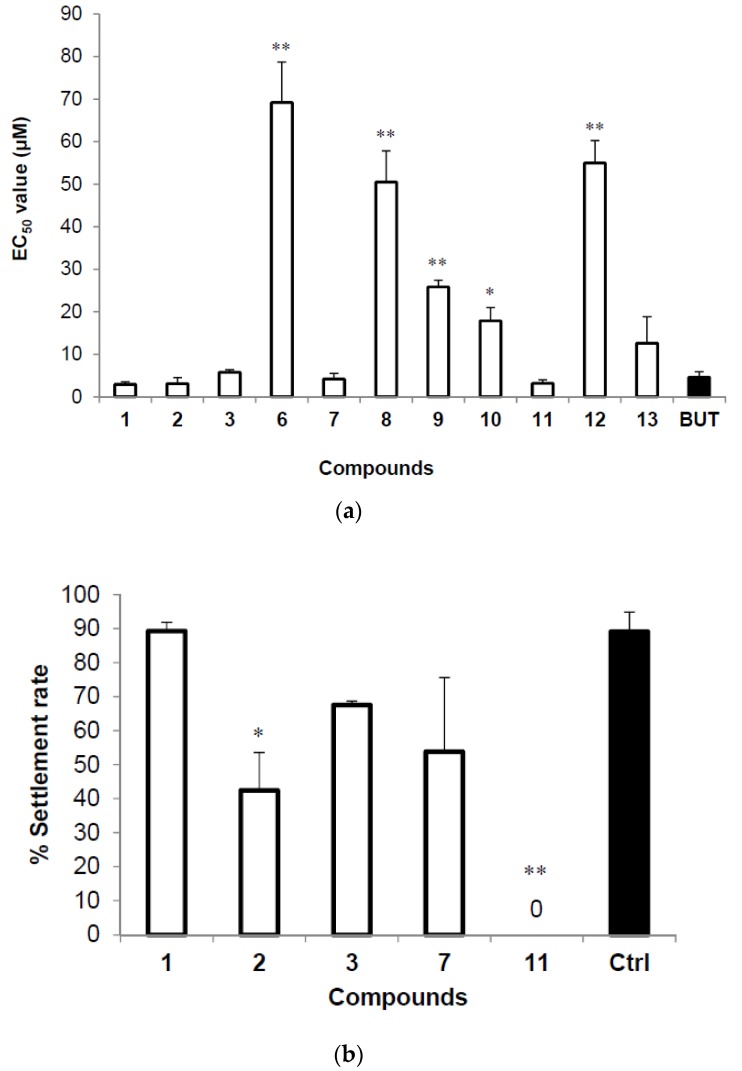
(**a**) EC_50_ of thielavin compounds against cyprids of *Balanus amphitrite*; Butenolide served as a positive control. (**b**) Recovery of *Balanus amphitrite* cyprid settlement in filtered seawater after a 24 h treatment with 10 μM of the thielavins we identified. The results were observed 48 h after being removed to filtered seawater. Values are presented as means ± SE of triplicate experiments. Asterisks indicate significant differences from control using Tukey’s test (* *p* < 0.05, ** *p* < 0.01, oneway ANOVA).

The anti-larval settlement activities of compounds **1**–**15** against cyprid larvae of *B. amphitrite* are summarized in Table 5. Compounds **1**–**3** and compounds **6**–**13** deterred larval settlement (Figure 3a). Due to the poor solubility of this group of compounds, we could not determine the LC_50_ values. Compounds **1**–**3**, **7**, and **11** also showed narcotic effects against cyprids of *B. amphitrite* at a concentration of 10 μM. Many organic molecules cause narcosis in barnacle larvae [11,12]. With increasing concentrations of active thielavins, cyprids lost their phototactic response, showed reduced appendage activity and were completely immobilized at a concentration of 10 μM. However, when the cyprids were transferred into 0.22 μM filtered seawater FSW following exposure to thielavins for 24 h, some recovered quickly from the chemical shock and completed their attachment and metamorphosis. The recovery rates of cyprids treated with 10 μM of compounds **1**–**3**, **7** and **11** demonstrated that larvae had the highest recovery rate from treatment with compound **1**, while no larvae recovered from treatment with compound **11** for 24 h (Figure 3b). Of all of the compounds, compound **1** showed excellent antifouling activity, and cyprids treated with this compound had the highest recovery rate. Thus, compound **1** is a promising natural antifoulant.

## 3. Materials and Methods

### 3.1. General Experimental Procedure

^1^H/^13^C and 2D-NMR spectral data were obtained using Varian Inova 500 MHz NMR spectrometers (Varian, Palo Alto, CA, USA). High-resolution mass spectra were acquired from UPLC-TOF-MS. The UPLC system was a Waters ACQUITY UPLC system (Waters, Manchester, UK) equipped with 150 mm × 2.1 mm Waters Acquity BEH C18 1.7-μm UPLC column and coupled to a Bruker micrOTOF-Q II mass spectrometer (Brucker Daltonics GmbH, Bremen, Germany). UV spectra were measured with a Shimadzu UV-2600 UV–vis spectrophotometer (Shimadzu, Kyoto, Japan) in an ACN solution. Semi-preparative reversed-phase HPLC was performed on a Waters 2695 liquid chromatography (Waters, Milford, CT, USA) with Luna C18(2) column (250 mm × 10 mm, 5 μm 100 Å, Phenomenex, Torrance, CA, USA). Column chromatography was performed on Rp-18 silica gel (Merck, Darmstadt, Germany).

### 3.2. Isolation and Identification of the Fungus

The fungal strain UST030930-004 was isolated from 12-d biofilms developed at the pier of the Hong Kong University of Science and Technology (Hong Kong, China) in Port Shelter. The biofilms were developed on polystyrene dishes submerged in seawater for 12 days in September 2003 and scraped from the dishes using a sterile glass coverslip. The scraped samples were suspended in 1 mL and 10 mL of autoclaved Ringer solution separately. For each sample, 200 μL of the mixture was spread on a Corn Meal Agar plate (Oxoid Ltd. Hampshire, UK) containing the antibiotics streptomycin and penicillin (final concentrations of 100 and 50 mg·L^−1^) to inhibit bacterial contamination. These plates were incubated at 27 °C for 7 to 14 days. The hyphal tip was transferred to new agar plates, incubated at 27 °C, and replated until a pure culture was obtained.

The total genomic DNA of the fungal isolate UST030930-004 was extracted as described by Lai et al. [13], and the internal transcribed spacer (ITS) gene sequences were amplified by the polymerase chain reaction (PCR) using primers ITS1 (5′-TCCGTAGGTGAACCTGCGG-3′) and ITS4 (5′-TCCTCCGCTTATTGAT ATGC-3′). The ITS region was sequenced and compared with reference sequences in GenBank by BLAST search, showing a similarity of 97% with *Thielavia terrestris* NRRL 8126 (CP003011). The ITS sequence of the fungal isolate UST030930-004 has been submitted to GenBank (Accession no. KJ716558). The fungal strain UST030930-004 was deposited in the China Center for Type Culture Collection (CCTCC) as CCTCC AF 2014015.

### 3.3. Fermentation, Bioassay-Guided Isolation, and Purification

The fungal strain UST030930-004 was cultured in a liquid medium containing 24 g·L^−1^ of potato-dextrose broth (Difco Laboratories, Detroit, MI, USA) and 20 g·L^−1^ of sea salts. Seed cultures were prepared in 50-mL Falcon tubes (BD Labware, Bedford, MA, USA), each containing 25 mL of medium, and cultivated at 23 °C for 3 days with shaking at 160 rpms. Afterwards, 2.8 L flasks, each containing 1.0 L of the same medium, were used for large-scale fermentation (16 L) with the following conditions: inoculation volume 5% (*v*/*v*), temperature 23 °C, rotation speed 160 rpms and a duration of 10 days on a shaker. The culture was filtered through 8 layers of cheesecloth to separate the filtrate and mycelia, which were treated separately. The filtrate was extracted with an equal volume of ethyl acetate (EA) three times, while the mycelial pellet was suspended in 80% acetone and sonicated using an ultrasonicator (Branson B-12, Danbury, CT, USA). The wet residue obtained after rotary evaporation to remove acetone was partitioned with EA. The filtrate and mycelial EA extracts were combined and evaporated in vacuo at 35 °C to dryness. The EA extract (22.0 g) was subjected to reversed-phase C18 flash chromatography and then eluted with solvents using a step gradient of H_2_O-MeOH to obtain fractions 1–5 (Fr. 1–5). Fr. 1–5 were tested for anti-larval settlement activity against *Balanus amphitrite*, and those showing potential activity were further purified. Fr. 3 was purified using semi-preparative HPLC (Phenomenex Luna C18 (2) 250 × 10 mm column) with MeOH/H_2_O (63:37, *v*/*v*) containing 5 mM ammonium acetate at the flow rate of 3 mL/min. In total, we obtained pure compounds **1** (2.4 mg), **2** (2.4 mg), **3** (3.9 mg), **4** (1.6 mg), **5** (4.2 mg), **6** (3.6 mg)**, 7** (2.0 mg), **8** (1.6 mg), **9** (0.8 mg), **10** (0.7 mg), **11** (0.7 mg), **12** (3.6 mg), **13** (4.4 mg), **14** (6.9 mg) and **15** (2.3 mg).

### 3.4. Spectral Data

Thielavin W (**1**): White powder, UV ACN *λ*_max_ 218.1, 267.8, 304.2 nm; ^1^H and ^13^C-NMR data, see Table 1; HRESIMS *m*/*z* 511.1604 [M + H]^+^ (calcd. for C_27_H_27_O_10_ 511.1599); ISCID data, see Table 4.

Thielavin X (**2**): White powder, UV ACN *λ*_max_ 218.1, 267.8, 306.5 nm; ^1^H and ^13^C-NMR data, see Table 1; HRESIMS *m*/*z* 525.1727 [M + H]^+^ (calcd. for C_28_H_29_O_10_ 525.1755); ISCID data, see Table 4.

Thielavin Y (**3**): White powder, UV ACN *λ*_max_ 218.1, 276.1, 304.7 nm; ^1^H and ^13^C-NMR data, see Table 1; HRESIMS *m*/*z* 525.1765 [M + H]^+^ (calcd. for C_28_H_29_O_10_ 525.1755); ISCID data, see Table 4.

Thielavin Z (**4**): White powder, UV ACN *λ*_max_ 218.1, 276.1, 306.5 nm; ^1^H and ^13^C-NMR data, see Table 1; HRESIMS *m*/*z* 525.1757 [M + H]^+^ (calcd. For C_28_H_29_O_10_ 525.1755); ISCID data, see Table 4.

Thielavin Z_1_ (**5**): White powder, UV ACN *λ*_max_ 218.1, 276.1, 306.9 nm; ^1^H and ^13^C-NMR data, see Table 2; HRESIMS *m*/*z* 525.1747 [M + H]^+^ (calcd. for C_28_H_29_O_10_ 525.1755); ISCID data, see Table 4.

Thielavin Z_2_ (**6**): White powder, UV ACN *λ*_max_ 218.1, 267.8, 305.2 nm; ^1^H and ^13^C-NMR data, see Table 2; HRESIMS *m*/*z* 539.1916 [M + H]^+^ (calcd. for C_29_H_31_O_10_ 539.1912); ISCID data, see Table 4.

Thielavin Z_3_ (**7**): White powder, UV ACN *λ*_max_ 214.6, 276.1, 309.1 nm; ^1^H and ^13^C-NMR data, see Table 2; HRESIMS *m*/*z* 525.1758 [M + H]^+^ (calcd. for C_28_H_29_O_10_ 525.1755); ISCID data, see Table 4.

Thielavin Z_4_ (**8**): White powder, UV ACN *λ*_max_ 218.1, 267.8, 305.2 nm; ^1^H and ^13^C-NMR data, see Table 2; HRESIMS *m*/*z* 511.1564 [M + H]^+^ (calcd. for C_27_H_27_O_10_ 511.1599); ISCID data, see Table 4.

Thielavin Z_5_ (**9**): White powder, UV ACN *λ*_max_ 213.4, 267.8, 307.0 nm; ^1^H and ^13^C-NMR data, see Table 3; HRESIMS *m*/*z* 525.1778 [M + H]^+^ (calcd. for C_28_H_29_O_10_ 525.1755); ISCID data, see Table 4.

Thielavin Z_6_ (**10**): White powder, UV ACN *λ*_max_ 215.8, 276.1, 307.0 nm; ^1^H and ^13^C-NMR data, see Table 3; HRESIMS *m*/*z* 587.1719/589.1646 [M + H]^+^ 3:1 (calcd. for C_30_H_32_ClO_10_ 587.1679); ISCID data, see Table 4.

Thielavin Z_7_ (**11**): White powder, UV ACN *λ*_max_ 215.8, 276.1, 308.9 nm; ^1^H and ^13^C-NMR data, see Table 3; HRESIMS *m*/*z* 375.1464 [M + H]^+^ (calcd. for C_20_H_23_O_7_ 375.1438); ISCID data, see Table 4.

### 3.5. Anti-Larval Settlement and Larval Recovery Assay

To prepare the larval culture of the barnacle *B. amphitrite*, adult brood stocks of *B. amphitrite* were collected from piling at the Pak Sha Wan Pier, Hong Kong (22°36′ N, 114°25′ E). Barnacle larvae were obtained and reared to cyprid stage as previously described by Harder et al. [14]. The cyprids were filtered and maintained in (FSW) at 4 °C overnight, before being used in the settlement assay. A stock solution of each extract or purified compound was prepared at 50 mg·mL^−1^ in dimethyl sulfoxide (DMSO) and further diluted to varying concentrations (25, 12.5, 6.25, 3.125, 1.562, 0.781 mg·mL^−1^) immediately before each bioassay. One milliliter of the test solutions was added to each well of a 24-well plate (Nunc, Thermo Scientific, Rochester, NY, USA), and approximately 15 larvae were gently pipetted into each well. For the negative controls, 1.0 mL of FSW together with 1.0 μL of DMSO, instead of the testing solution, was added to each well prior to the addition of larvae. The plates were incubated at 28 °C for 48 h in the dark. At the end of the incubation, the numbers of settled and swimming larvae were counted directly under a microscope, and where appropriate, potential toxic effects were noted. The number of settled larvae was expressed as a percentage of the total number of larvae per well. Three replicates of each extract concentration or purified compound were evaluated. EC_50_ represents the concentration of a compound that inhibits settlement of 50% of the larval population compared with the negative control, while the LC_50_ represents the concentration of a compound that is required to kill 50% of the larvae in a tested population. The experiments were performed in triplicate.

In the recovery assay, cyprids used in the negative controls were placed in new polypropylene containers containing FSW for 24 h, as described in the literature [15]. One milliliter of 10 μM of the test solutions was added to each well of a 24-well plate, and approximately 15 larvae were added to each well. After a 24-h incubation at 28 °C in the dark, the tested solutions were removed, and the treated larvae were washed 3 times with FSW prior to being plated in a new 24-well plate. For the negative controls, swimming larvae in a polypropylene container were transferred to 1.0 mL of FSW containing 1.0 μL of DMSO. The plates were maintained at 28 °C for 48 h before determining the percentage of attached cyprids of *B. amphitrite*. Three replicates of each experimental concentration were assessed.

## 4. Conclusions

Thielavins are para-depside-type compounds originally isolated from *Thielavia terricola* [10]. To date, 23 thielavins (thielavin A–V) have been discovered, including two thielavin Qs [16,17,18]. The thielavins act as indoleamine 2,3-dioxygenase, prostaglandin synthetase, glucose-6-phosphatase, cell wall transglycosylation and telomerase inhibitors [9,10,16,19,20]. We have not found any description of the antifouling activity of thielavin compounds. In the present study, we isolated 15 thielavins from a marine-derived fungus *Thielavia* sp. UST030930-004, including 11 new ones, and 11 of them had antifouling activities. Of all of the compounds, Thielavin W (**1**) showed excellent antifouling activity, and cyprids treated with this compound had the highest recovery rate. Thus, Thielavin W is a promising natural antifoulant.

## Figures and Tables

**Table:** 

	R_1_	R_2_	R_3_	R_4_	R_5_	R_6_	R_7_
**1**	H	H	Me	Me	Me	H	H
**2**	H	H	Me	Me	Me	H	Me
**3**	Me	H	Me	Me	H	H	Me
**4**	Me	H	H	Me	Me	H	Me
**5**	Me	H	Me	H	Me	H	Me
**6**	Me	H	Me	H	Me	Me	Me
**7**	Me	H	Me	Me	Me	H	H
**8**	H	H	Me	H	Me	H	Me
**9**	H	H	Me	H	Me	Me	Me
**10**	Me	Cl	Me	Me	Me	Me	Me
**12**	Me	H	Me	Me	Me	Me	H
**13**	H	H	Me	Me	Me	Me	H
**14**	Me	H	Me	Me	Me	H	Me
**15**	Me	H	Me	Me	Me	Me	Me

**Table 1:** The ^1^H (500 MHz) and ^13^C-NMR (125 MHz) NMR data for compounds **1**–**4** (*δ* in ppm, DMSO-*d*_6_).

Position	1		2		3		4	
	*δ*_H_	*δ*_C_	*δ*_H_	*δ*_C_	*δ*_H_	*δ*_C_	*δ*_H_	*δ*_C_
1		138.7		139.0		142.1		138.7
2		165.4		164.8		161.2		165.1
3		114.2		114.3	6.36 d 2.0	107.0		114.4
4		149.5		150.8		151.5		149.8
5	6.18 s	110.3	6.21 s	111.2	6.29 d 2.0	111.3	6.41 s	111.3
6		114.7		115.5		117.5		115.3
1′		131.8		132.2		132.4		135.5
2′		150.7		153.2		153.2		154.5
3′		116.5		121.8		121.6	7.01 s	104.2
4′		149.1		149.5		148.9		149.9
5′		120.8		125.8		125.6		121.2
6′		121.6		126.2		126.4		121.7
1″		141.0		141.0		139.5		139.3
2″		161.4		161.4		162.7		162.2
3″	6.26 s	100.6	6.27 s	100.6		108.7		114.4
4″		160.8		160.6		160.6		160.9
5″	6.26 s	110.3	6.27 s	110.2	6.44 s	111.3	6.14 s	111.3
6″		107.6		107.2		102.8		103.5
1-C=O		172.0 *		172.5 *		170.7 *		171.9 *
2-OMe								
3-Me	1.99 s	9.4	1.99 s	9.0			1.98 s	8.1
5-Me								
6-Me	2.52 s	23.1	2.40 s	23.0	2.56 s	24.1	2.55 s	23.8
1′-C=O		167.1 *		166.9 *		169.3 *		169.7 *
2′-OMe			3.78 s	62.0	3.79 s	62.0	3.82 s	56.3
3′-Me	2.06 s	10.2	2.13 s	10.0	2.08 s	9.9		
5′-Me	2.06 s	12.5	2.12 s	12.7	2.06 s	12.6	2.03 s	12.0
6′-Me	2.29 s	16.7	2.31 s	16.6	2.29 s	16.5	2.32 s	16.8
1″-C=O		166.7 *		165.9 *		165.9 *		165.7 *
3″-Me					1.99 s	8.1	1.96 s	9.1
5″-Me								
6″-Me	2.40 s	21.8	2.54 s	21.8	2.55 s	21.8	2.52 s	23.1

NMR assignments marked by an asterisk (*) are interchangeable.

**Table 2:** The ^1^H (500 MHz) and ^13^C-NMR (125 MHz) NMR data for compounds **5**–**8** (*δ* in ppm, DMSO-*d*_6_).

Position	5		6		7		8	
	*δ*_H_	*δ*_C_	*δ*_H_	*δ*_C_	*δ*_H_	*δ*_C_	*δ*_H_	*δ*_C_
1		139.1		139.1		138.9		138.9
2		156.2		156.7		162.0		165.2
3		115.4		115.4		116.5		114.2
4		151.0		150.7		151.7		149.5
5	6.40 s	113.2		118.6	6.64 s	115.8	6.16 s	109.8
6		114.1		120.4		112.1		115.8
1′		134.3		134.8		132.0		134.0
2′		156.2		156.2		150.7		156.1
3′		122.0		122.2		116.5		122.0
4′		150.6		149.3		148.8		150.8
5′	7.10 s	120.1	7.12 s	120.4		120.7	7.00 s	119.9
6′		125.7		125.4		121.7		126.0
1″		139.1		139.1		139.5		140.4
2″		162.0		162.0		162.7		160.0
3″		108.6		108.6		108.6	6.25 d 2.0	100.7
4″		160.9		160.9		161.0		161.1
5″	6.40 s	111.1	6.41 s	111.0	6.42 s	111.2	6.24 d 2.0	109.9
6″		103.6		103.7		102.8		108.3
1-C=O		172.7 *		171.5 *		173.0 *		171.7 *
2-OMe								
3-Me	2.03 s	9.1	2.08 s	9.9				
5-Me			2.10 s	12.6	2.10 s	9.2	1.97 s	9.3
6-Me	2.52 s	23.6	2.40 s	17.4				
1′-C=O		169.4 *		169.4 *	2.50 s	22.9	2.53 s	23.1
2′-OMe	3.82 s	61.9	3.79 s	61.9		169.5 *		167 *
3′-Me	2.11 s	9.6	2.13 s	9.6			3.81 s	62.0
5′-Me					2.02 s	10.1	2.14 s	9.6
6′-Me	2.4 s	18.7	2.40 s	19.2	2.01 s	12.4		
1″-C=O		165.1 *		165.0 *	2.30 s	16.7	2.39 s	18.6
3″-Me	1.98 s	8.1	1.98 s	8.1		166.4 *		165.4 *
5″-Me					1.98 s	8.0		
6″-Me	2.52 s	23.0	2.53 s	23.6				

NMR assignments marked by an asterisk (*) are interchangeable.

**Table 3:** The ^1^H (500 MHz) and ^13^C-NMR (125 MHz) NMR data for compounds **9**–**11** (*δ* in ppm, DMSO-*d*_6_).

Position	9		10		11	
	*δ*_H_	*δ*_C_	*δ*_H_	*δ*_C_	*δ*_H_	*δ*_C_
1		136.8		138.3		132.1
2		161.3		160.0		150.7
3		113.3		116.5		116.0
4		148.6		151.8		148.5
5		115.5		120.6		120.1
6		117.5		115.7		121.4
1′		134.5		134.1		139.4
2′		156.6		155.0		162.6
3′		122.2		122.7		108.6
4′		150.9		150.3		161.0
5′	7.01 s	120.2		126.4	6.40 s	111.0
6′		125.7		127.5		102.7
1″		140.3		136.2		
2″		161.1		* 160.8		
3″	6.23 s	100.5		112.8		
4″		161.2		* 157.9		
5″	6.23 s	109.9		107.2		
6″		108.3		116.2		
1-C=O		171.8 *		UD		168.7 *
1-COOMe					3.83 s	51.9
2-OMe						
3-Me	2.01 s	9.8	2.62 s	9.6	1.96 s	9.7
5-Me	2.04 s	12.5	2.42 s	12.6	1.96 s	12.3
6-Me	2.58 s	17.4	2.84 s	17.8	2.13 s	16.6
1′-C=O		167.1 *		UD		168.9
2′-OMe	3.78 s	62.0	3.90 s	61.9		
3′-Me	2.16 s	9.7	2.36 s	9.7	1.97 s	7.8
5′-Me			2.21 s	12.6		
6′-Me	2.40 s	19.1	2.44 s	16.7	2.54 s	24.0
1″-C=O		165.8 *		UD		
3″-Me			2.57 s	9.2		
5″-Me						
6″-Me	2.39 s	21.4	2.94 s	19.4		

NMR assignments marked by an asterisk (*) are interchangeable. UD: undetected. ^13^C-NMR signals were not observed due to a limited amount of compound **10**.

**Table 4:** Physicochemical properties of new thielavins.

	1	2	3	4
Molecular Formula	C_27_H_27_O_10_	C_28_H_29_O_10_	C_28_H_29_O_10_	C_28_H_29_O_10_
HRESIMS (pos) Obsd. (*m*/*z*)	511.1604	525.1727	525.1765	525.1757
Cald. (*m*/*z*)	511.1599	525.1755	525.1755	525.1755
ISCID (*m*/*z*)		493.2	507.2	507.1
	361.1	375.2	361.1	361.1
	329.1	343.1	357.1	343.1
	179.1	193.1	193.1	179.1
	151.0	151.0	165.1	165.1
UV	218.1	218.1	218.1	218.1
	267.8	267.8	276.1	276.1
	304.2	306.5	304.7	306.5
	**5**	**6**	**7**	**8**
Molecular Formula	C_28_H_29_O_10_	C_29_H_31_O_10_	C_28_H_29_O_10_	C_27_H_27_O_10_
HRESIMS (pos) Obsd. (*m*/*z*)	525.1747	539.1916	525.1758	511.1564
Cald. (*m*/*z*)	525.1755	539.1912	525.1755	511.1599
ISCID (*m*/*z*)		507.2		479.1
	361.1	375.2	361.1	361.1
	343.1	343.1	343.1	329.1
	179.1	179.1	179.1	179.1
	165.1	165.1	165.1	151.0
UV	218.1	218.1	214.6	218.1
	276.1	276.8	276.1	267.8
	306.9	305.2	309.1	305.2
	**9**	**10**	**11**	
Molecular Formula	C_28_H_29_O_10_	C_30_H_32_ClO_10_	C_20_H_23_O_7_	
HRESIMS (pos) Obsd. (*m*/*z*)	525.1778	587.1719/589.1646	375.1464	
Cald. (*m*/*z*)	525.1755	587.1679	375.1438	
ISCID (*m*/*z*)		389.1		
	375.2	391.1/393.1		
	329.1	199.0/201.0	211.1	
	179.1	193.1	179.1	
	151.0		165.1	
UV	213.4	215.8	215.8	
	267.8	276.1	276.1	
	307.0	307.0	308.9	

**Table 5:** Anti-larval settlement activity of compounds from the fungus *Thielavia* sp. UST030930-004 against *Balanus amphitrite*. EC_50_ means the settlement of 50% of the larval population was inhibited, compared with the negative control. Data presented are the mean ± SE of three independent experiments performed in triplicate. Butenolide was served as the positive control.

Compounds	EC_50_ (μM)	Compounds	EC_50_ (μM)
**1**	2.95 ± 0.59	**9**	25.86 ± 1.56
**2**	3.13 ± 1.37	**10**	17.86 ± 3.14
**3**	5.78 ± 0.60	**11**	3.20 ± 0.83
**6**	69.19 ± 9.51	**12**	54.99 ± 5.23
**7**	4.23 ± 1.30	**13**	12.64 ± 6.20
**8**	50.50 ± 7.35	Butenolide	4.62 ± 1.30

## Supplementary Materials

The following are available at www.mdpi.com/1660-3397/15/5/128/s1. All 1D, 2D-NMR, HRESIMS and ISCID-MS/MS spectra of compounds **1**–**11** are included.
