# Supplementary material for: Thielavins W–Z7, New Antifouling Thielavins from the Marine-Derived Fungus Thielavia sp. UST030930-004
Source: Mar Drugs. 2017 Apr 29;15(5):128. doi: 10.3390/md15050128 (PMC5450534; doi:10.3390/md15050128)
Supplement: Supplementary file 1 [file marinedrugs-15-00128-s001.pdf]

Thielavins W–Z$_7$, New Antifouling Thielavins from the Marine-Derived Fungus *Thielavia* sp. UST030930-004

Zhuang Han [1,2], Yong-Xin Li [2], Ling-Li Liu [2], Liang Lu [2], Xian-Rong Guo [3], Xi-Xiang Zhang [4], Xiao-Yong Zhang [5], Shu-Hua Qi [5], Ying Xu [2,6,*], Pei-Yuan Qian [2,*]

1 Institute of Deep-sea Science and Engineering, Chinese Academy of Sciences, 28 Luhuitou Road, Sanya 572000, China; zhuanghan@idsse.ac.cn
2 Division of Life Science, The Hong Kong University of Science and Technology, Clear Water Bay, Hong Kong, China; liyongxin@connect.ust.hk (Y.-X.L.); leonie@nwsuaf.edu.cn (L.-L.L.); luliangust@163.com (L.L.)
3 Imaging & Characterization Core lab, King Abdullah University of Science and Technology, Thuwal 23955-6900, Saudi Arabia; xianrong.guo@kaust.edu.sa
4 Physical Science and Engineering, King Abdullah University of Science and Technology, Thuwal 23955-6900, Saudi Arabia; xixiang.zhang@kaust.edu.sa
5 Key Laboratory of Marine Bio-resources Sustainable Utilization, South China Sea Institute of Oceanology, Chinese Academy of Sciences, 164 West Xingang Road, Guangzhou 510301, China; zhangxiaoyong@scsio.ac.cn (X.-Y.Z.); shuhuaqi@scsio.ac.cn (S.-H.Q.)
6 College of Life Science, Shenzhen University, 3688 Nanhai Ave, Shenzhen 518060, China
* Correspondence: boxuying@szu.edu.cn (Y.X.); boqianpy@ust.hk (P.-Y.Q.); Tel.: +852-23587331 (P.-Y.Q.); Fax: +852-23581559 (P.-Y.Q.)

Figure S1-S7: 1D, 2D NMR, HRESIMS and ISCID spectra of Compound **1**
Figure S8-S14: 1D, 2D NMR, HRESIMS and ISCID spectra of Compound **2**
Figure S15-S21: 1D, 2D NMR, HRESIMS and ISCID spectra of Compound **3**
Figure S22-S28: 1D, 2D NMR, HRESIMS and ISCID spectra of Compound **4**
Figure S29-S35: 1D, 2D NMR, HRESIMS and ISCID spectra of Compound **5**
Figure S36-S42: 1D, 2D NMR, HRESIMS and ISCID spectra of Compound **6**
Figure S43-S49: 1D, 2D NMR, HRESIMS and ISCID spectra of Compound **7**
Figure S50-S56: 1D, 2D NMR, HRESIMS and ISCID spectra of Compound **8**
Figure S57-S63: 1D, 2D NMR, HRESIMS and ISCID spectra of Compound **9**
Figure S64-S69: 1D, 2D NMR, HRESIMS and ISCID spectra of Compound **10**
Figure S70-S75: 1D, 2D NMR, HRESIMS and ISCID spectra of Compound **11**

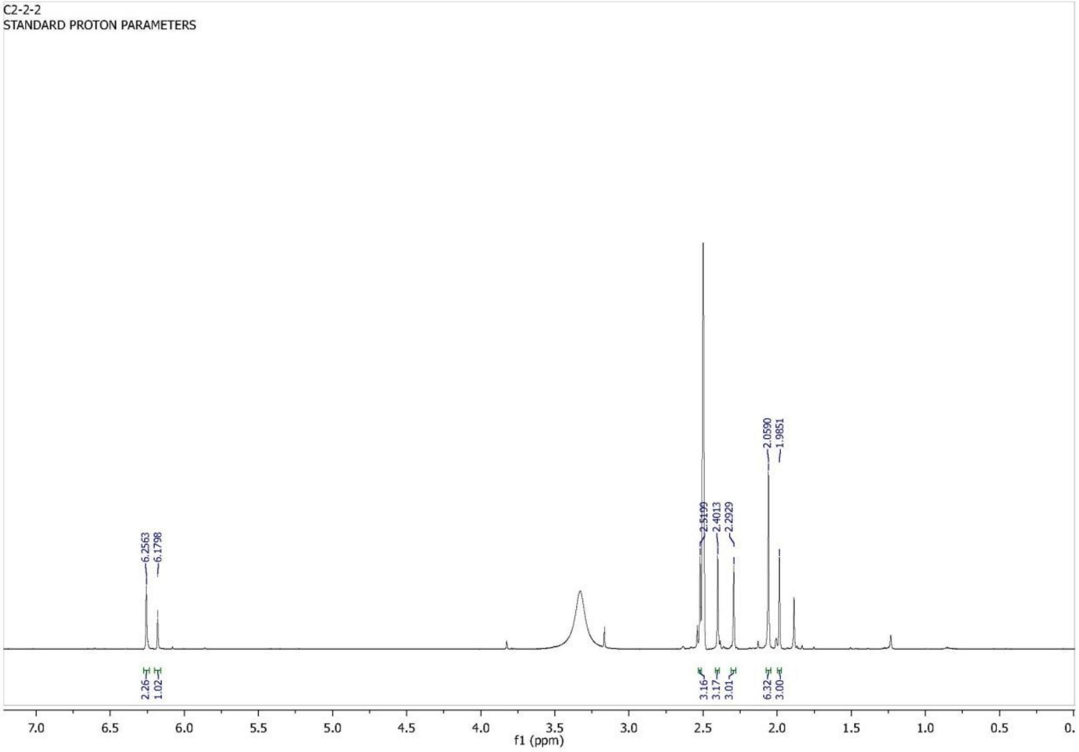

Figure S1 ^{1}H NMR spectrum of **1** in DMSO-d_6 (500 MHz)

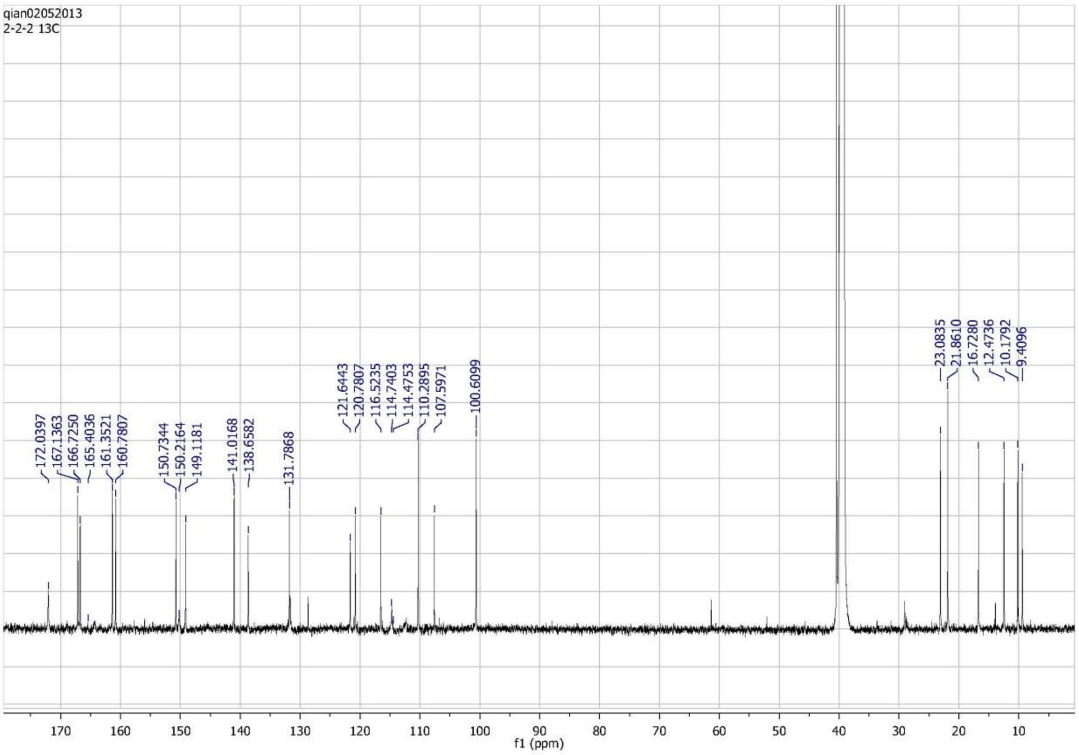

Figure S2 ^{13}C NMR spectrum of **1** in DMSO-d_6 (125 MHz)

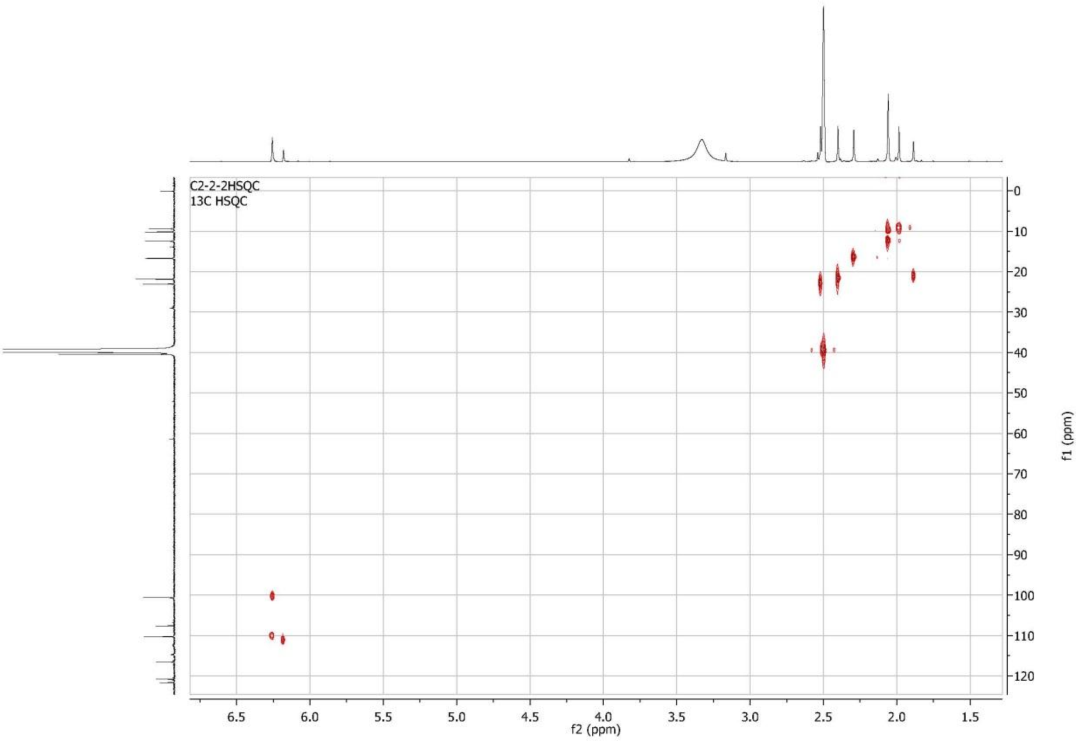

Figure S3 HMQC spectrum of **1** in DMSO-d_6

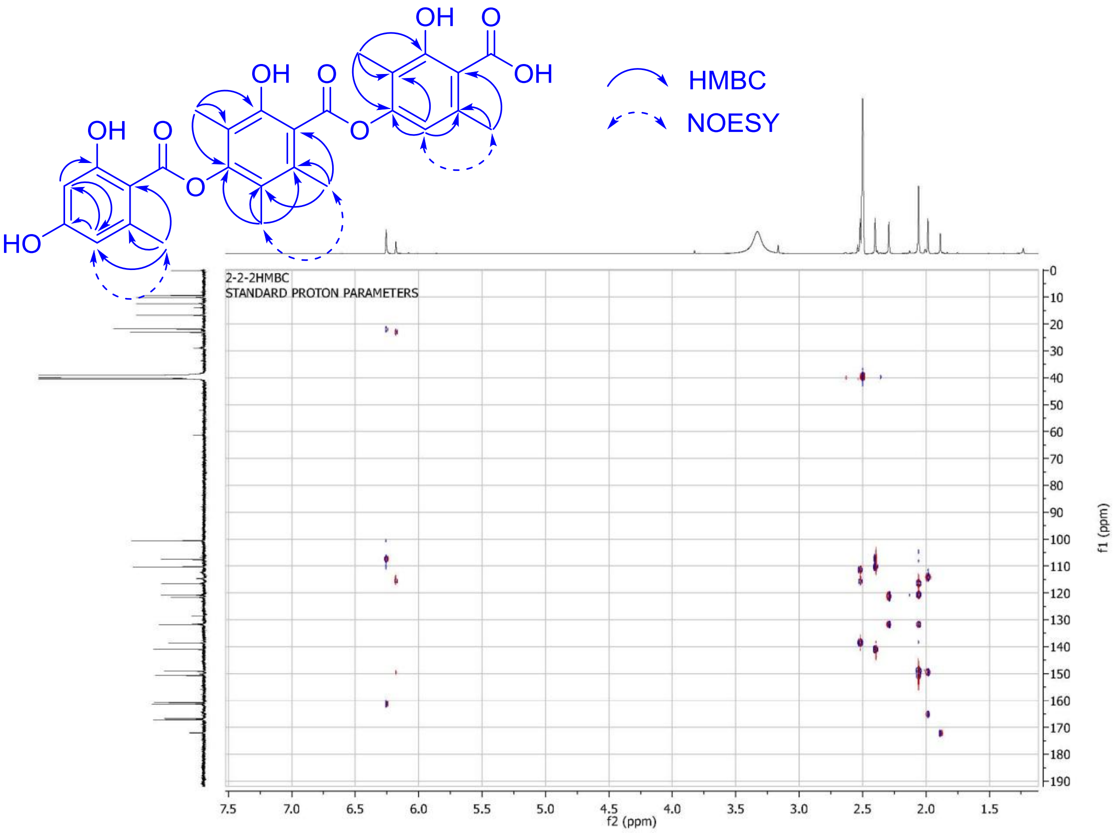

Figure S4 HMBC spectrum of **1** in DMSO-d_6

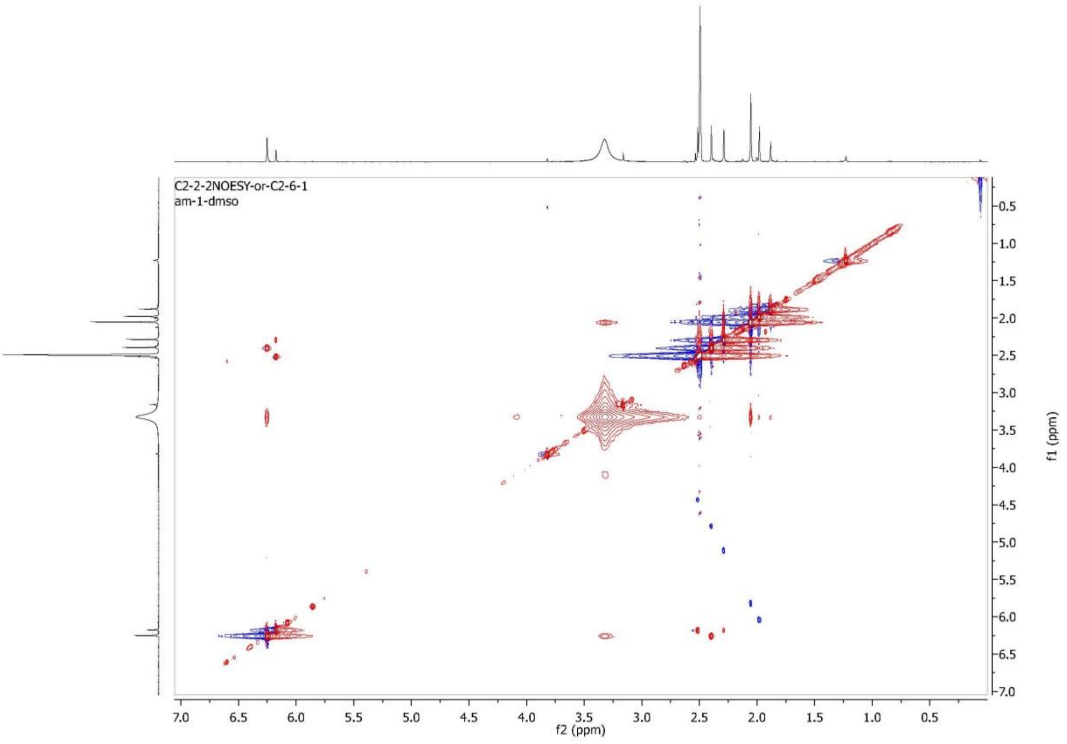

Figure S5 NOESY spectrum of **1** in DMSO-d_6

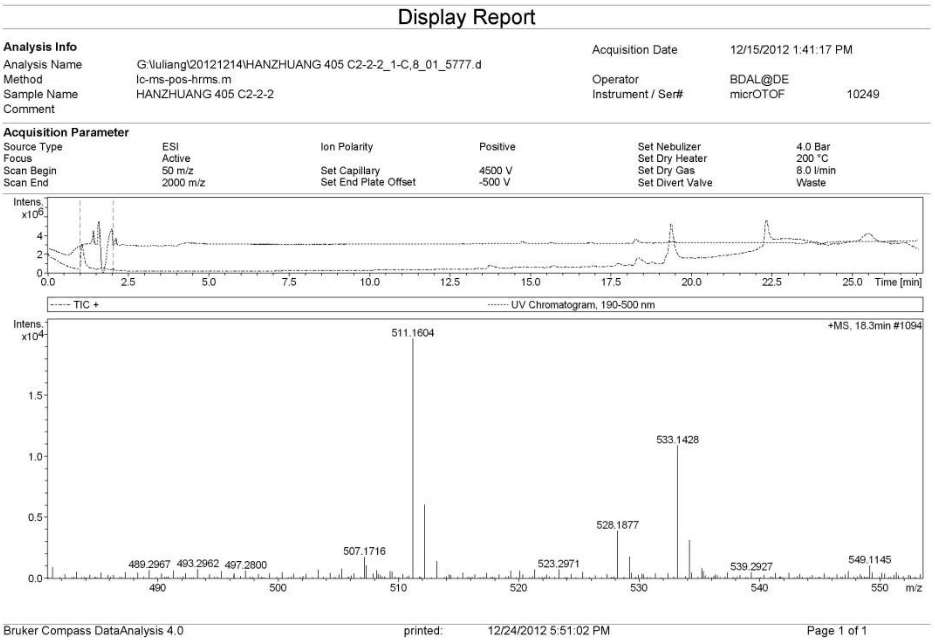

Figure S6 HRESIMS spectrum of **1**

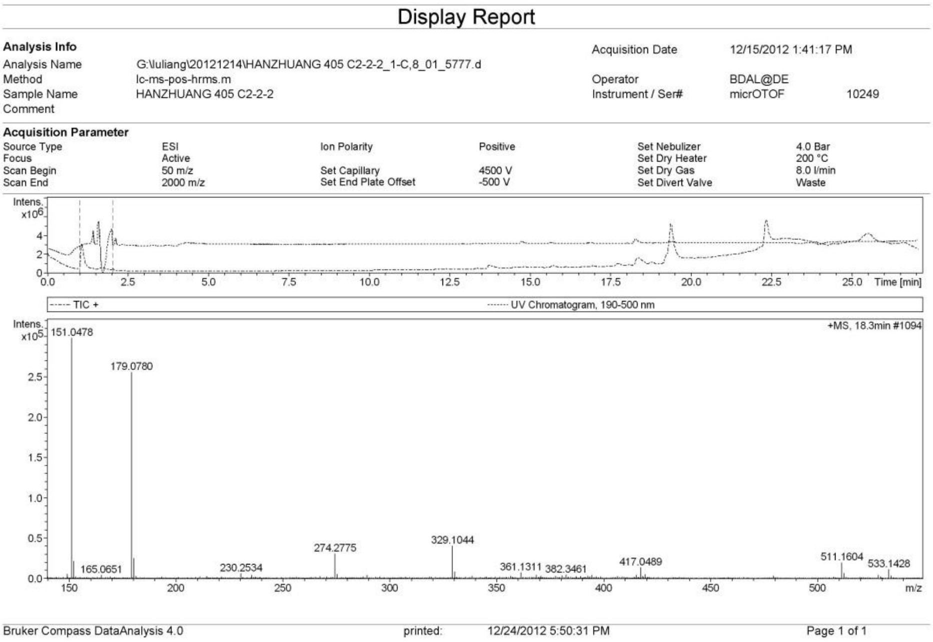

Figure S7 ISCID spectrum of **1**

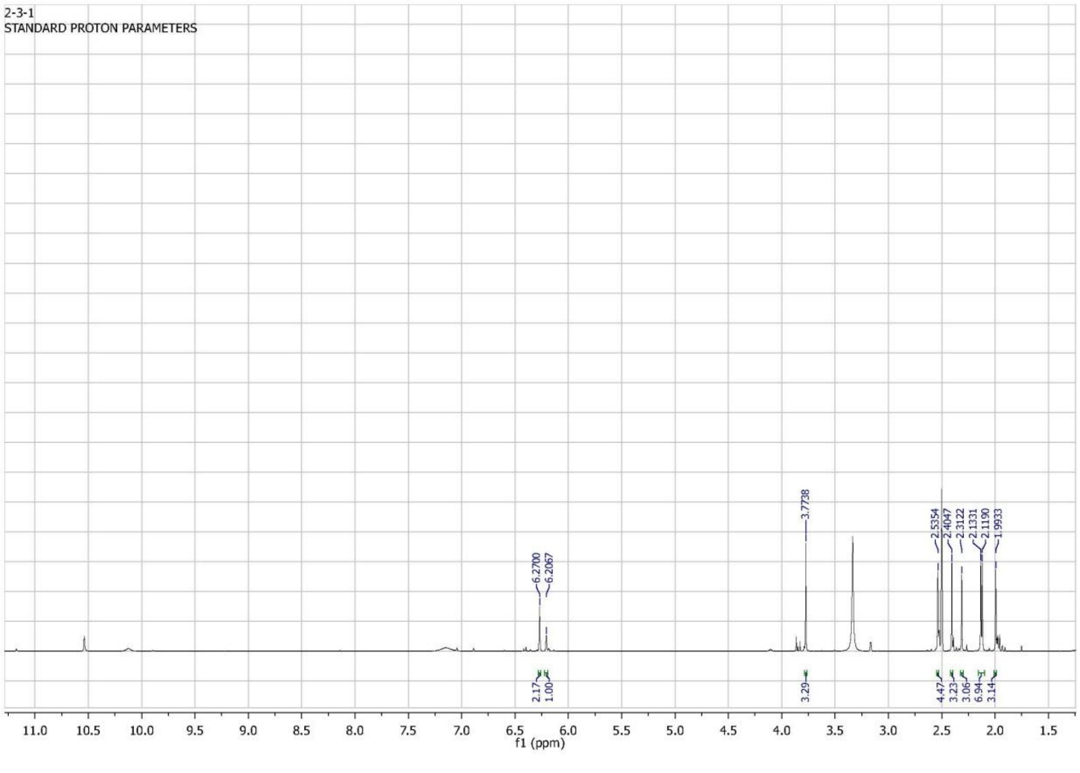

Figure S8 ^{1}H NMR spectrum of **2** in DMSO-d_6 (500 MHz)

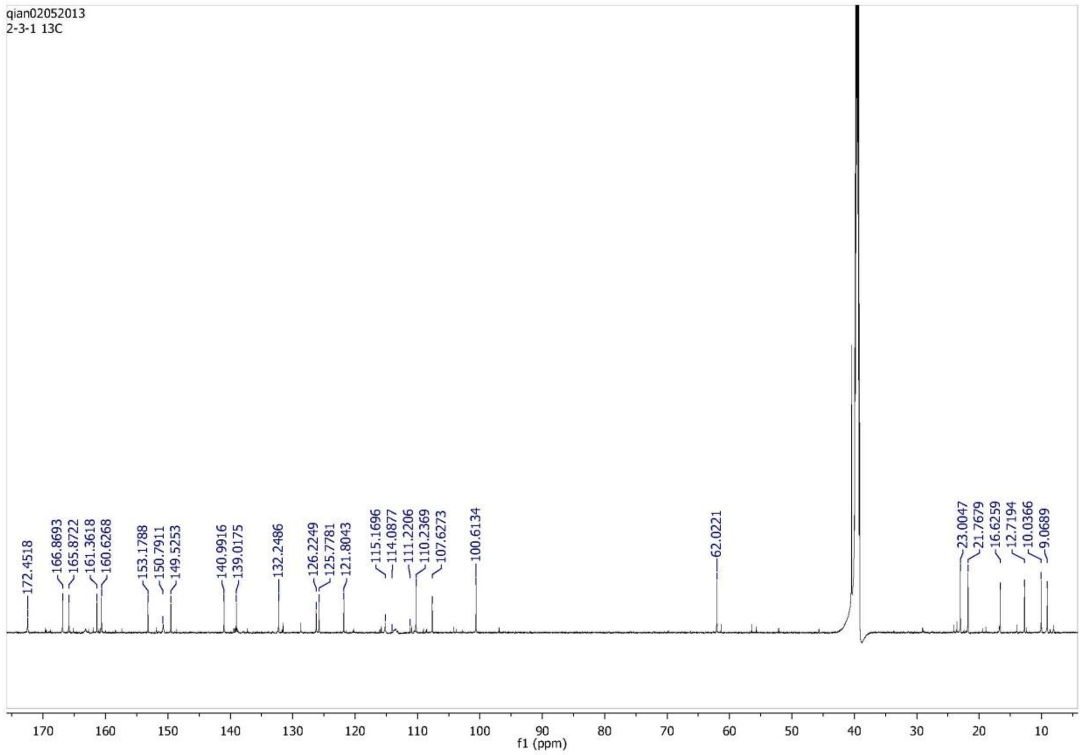

Figure S9 ^{13}C NMR spectrum of **2** in DMSO-d_6 (125 MHz)

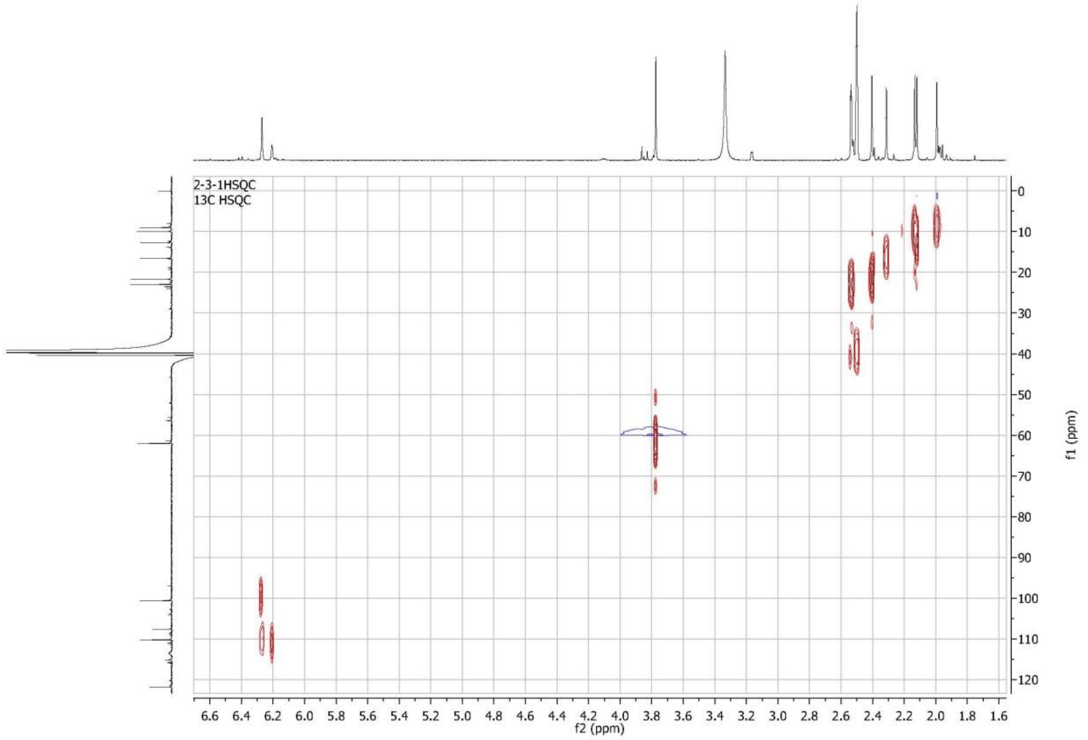

Figure S10 HMQC spectrum of **2** in DMSO-d_6

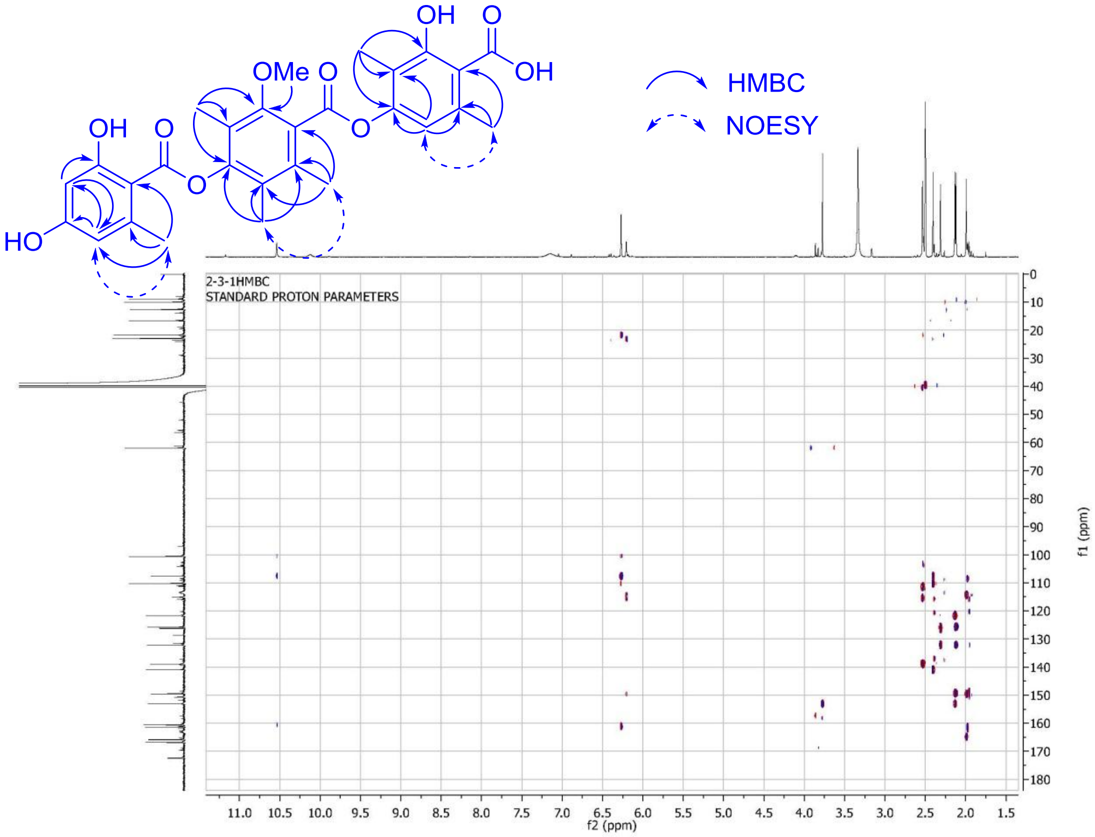

Figure S11 HMBC spectrum of **2** in DMSO-d_6

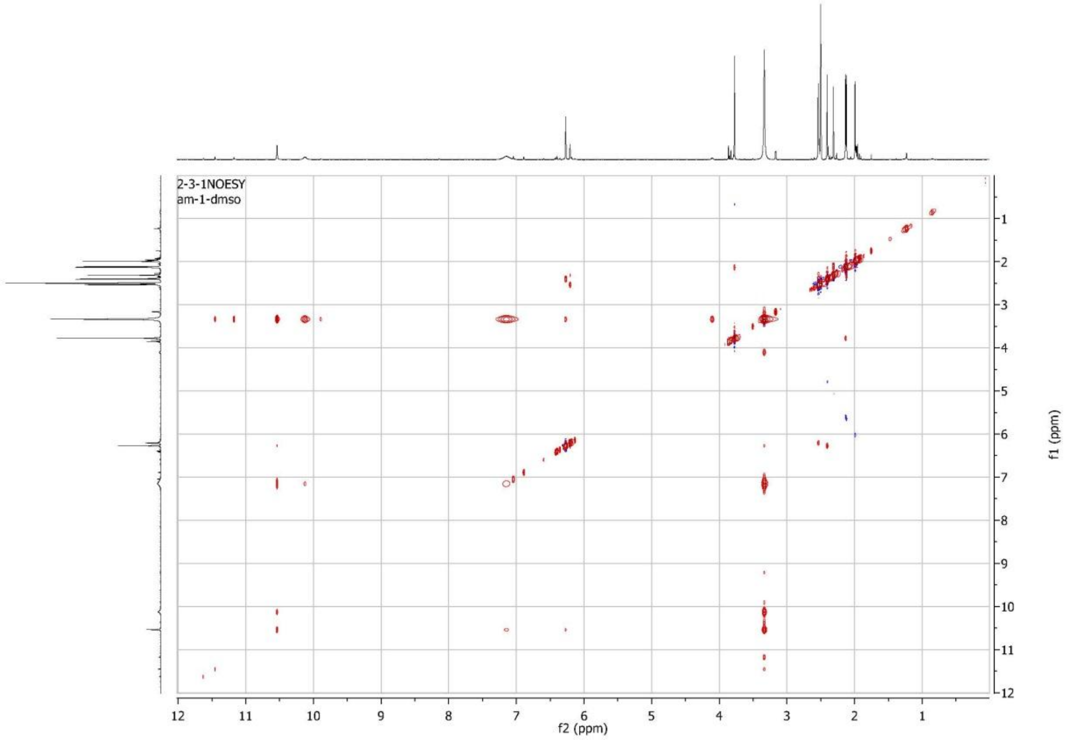

Figure S12 NOESY spectrum of **2** in DMSO-d_6

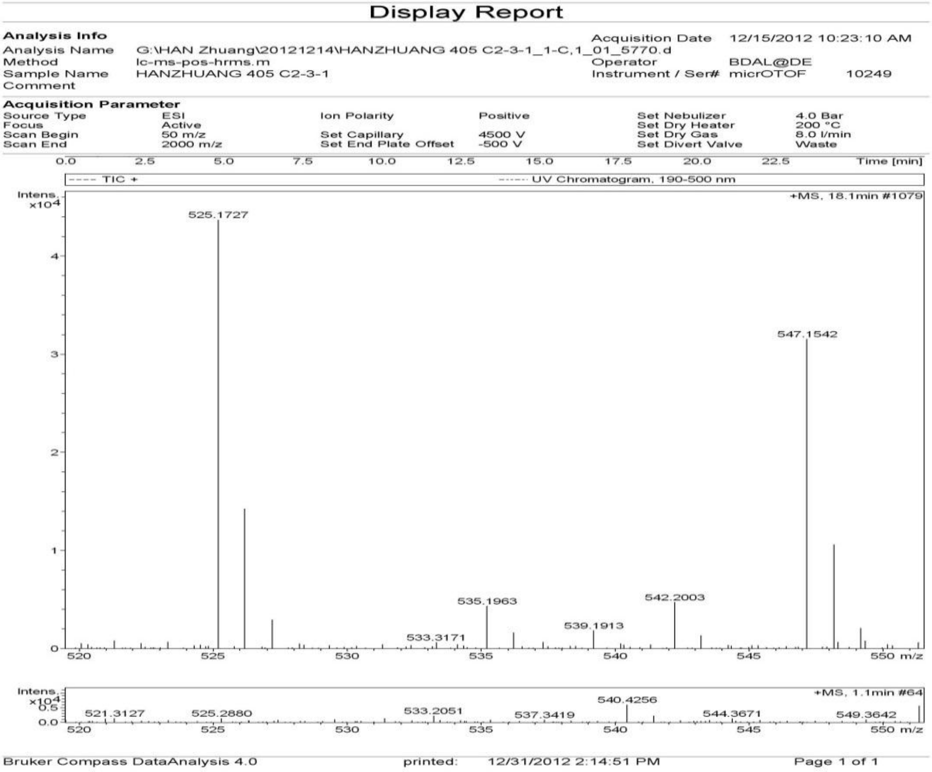

Figure S13 HRESIMS spectrum of **2**

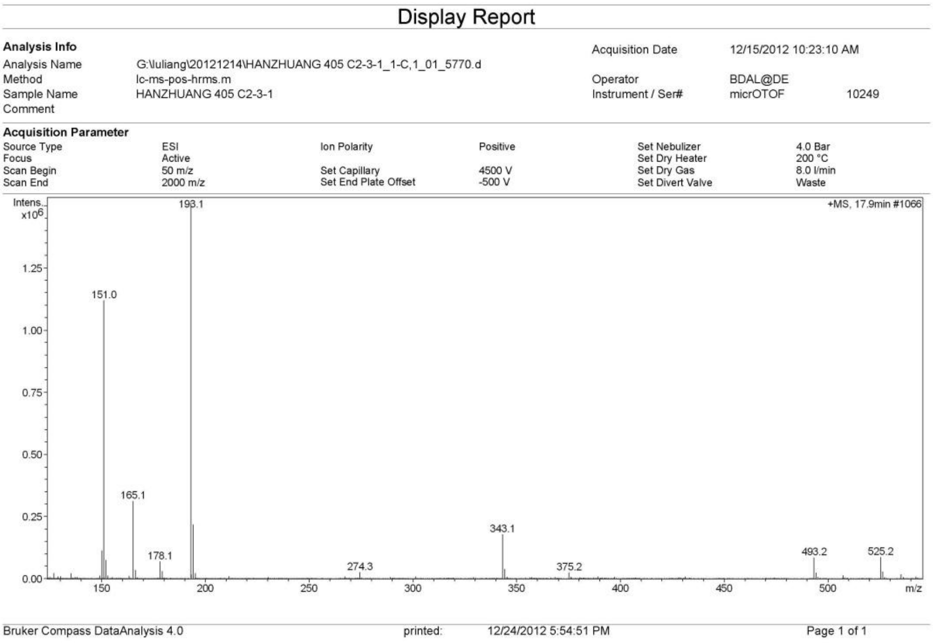

Figure S14 ISCID spectrum of **2**

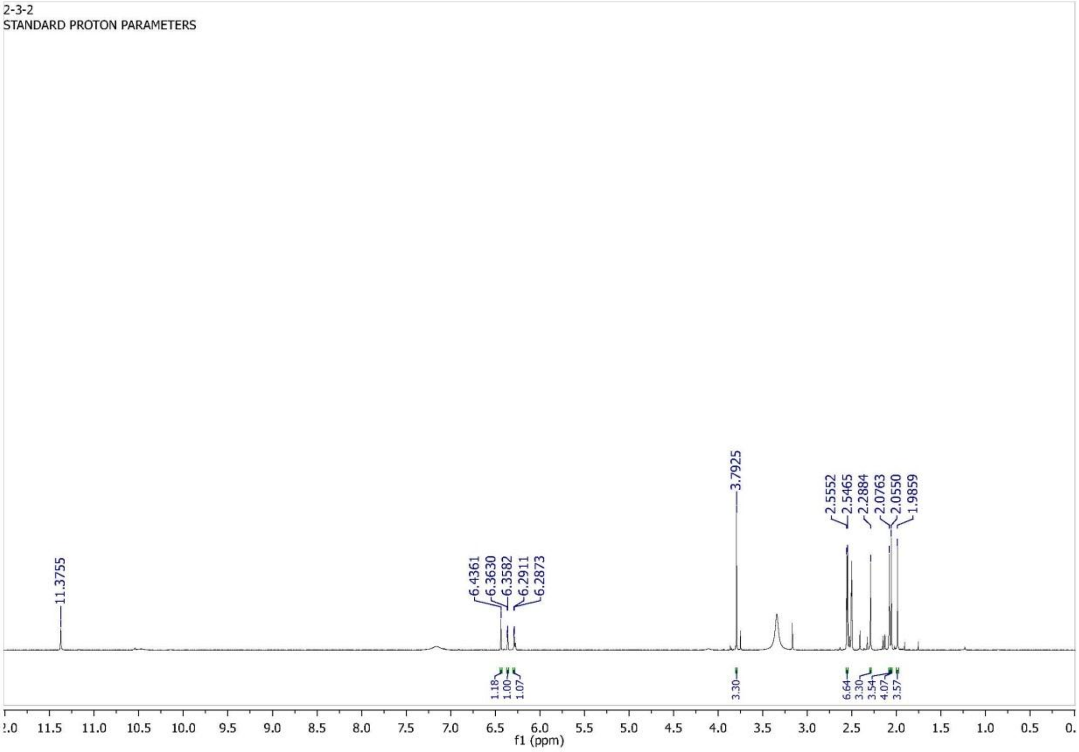

Figure S15 ^{1}H NMR spectrum of **3** in DMSO-d_6 (500 MHz)

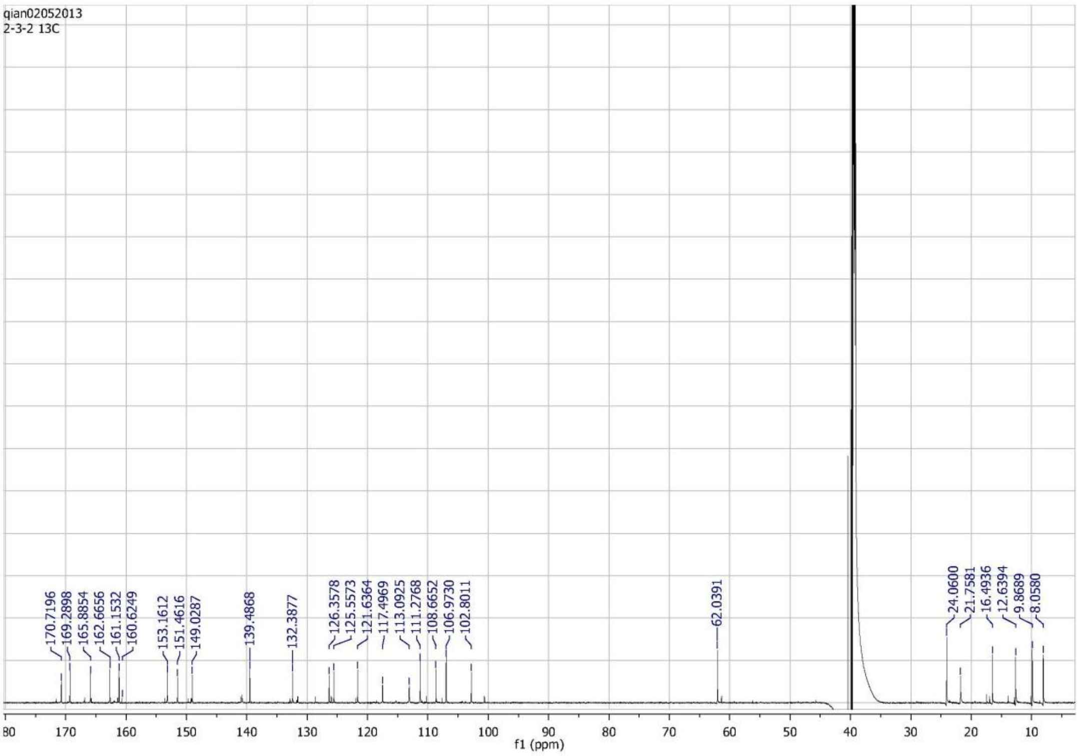

Figure S16 ^{13}C NMR spectrum of **3** in DMSO-d_6 (125 MHz)

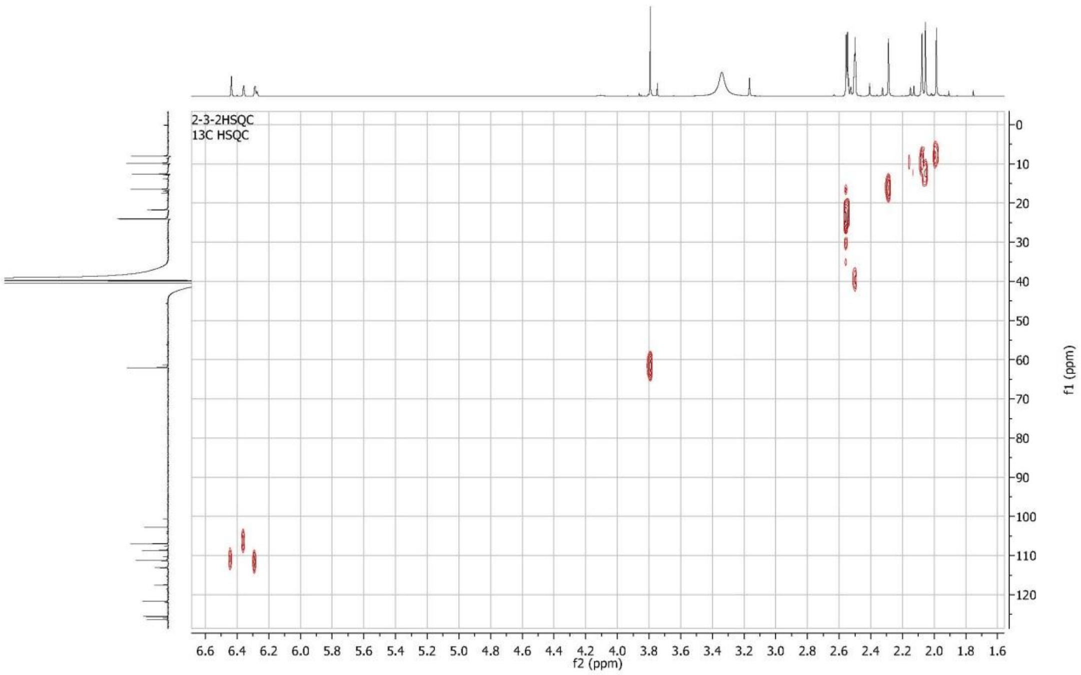

Figure S17 HMQC spectrum of **3** in DMSO-d_6

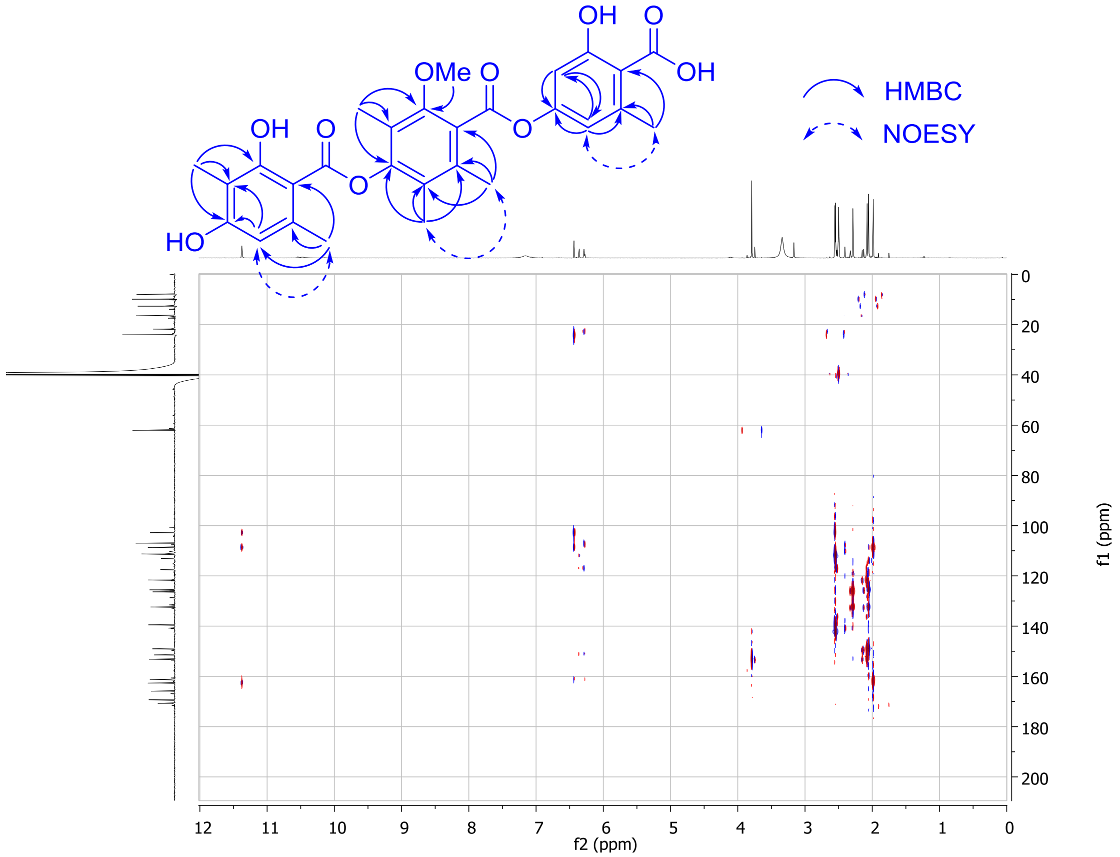

Figure S18 HMBC spectrum of **3** in DMSO-d_6

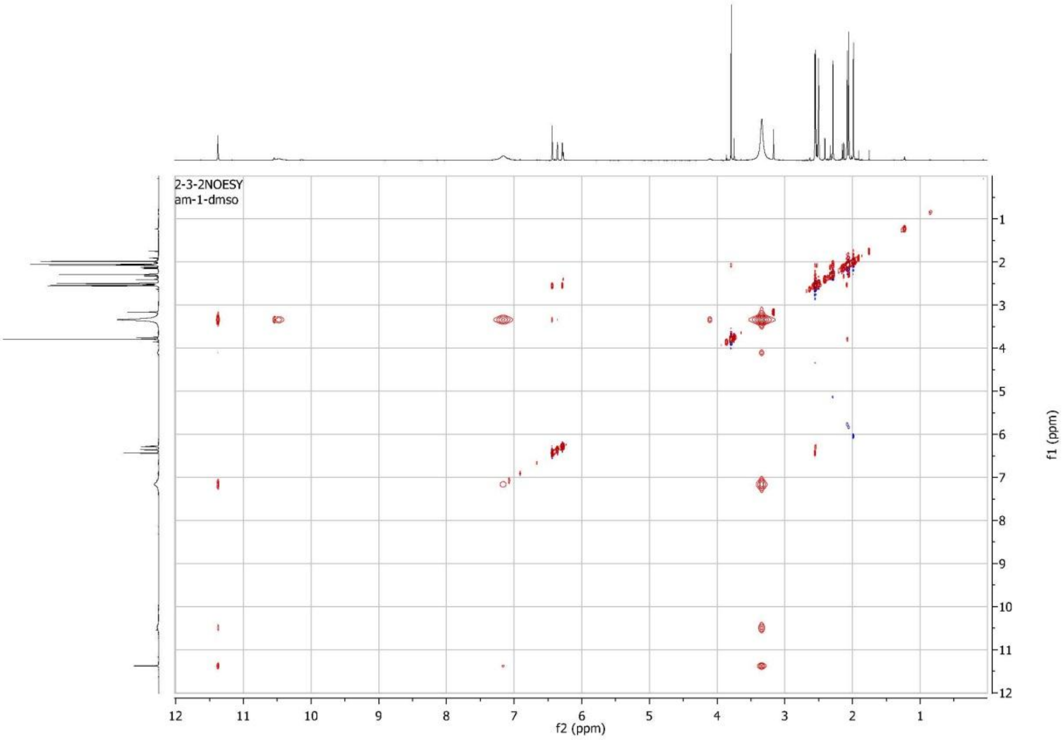

Figure S19 NOESY spectrum of **3** in DMSO-d_6

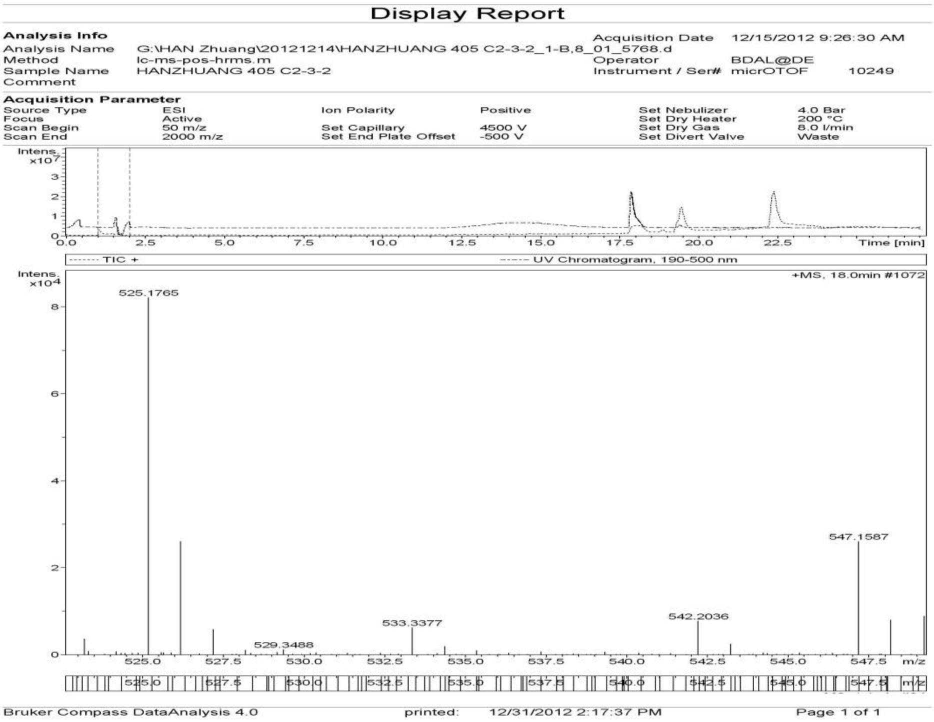

Figure S20 HRESIMS spectrum of **3**

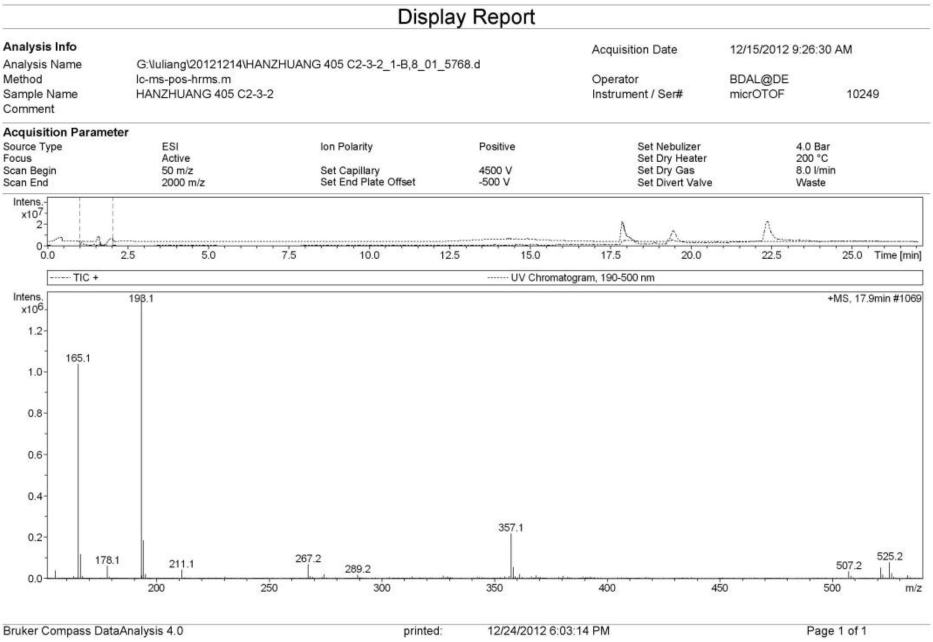

Figure S21 ISCID spectrum of **3**

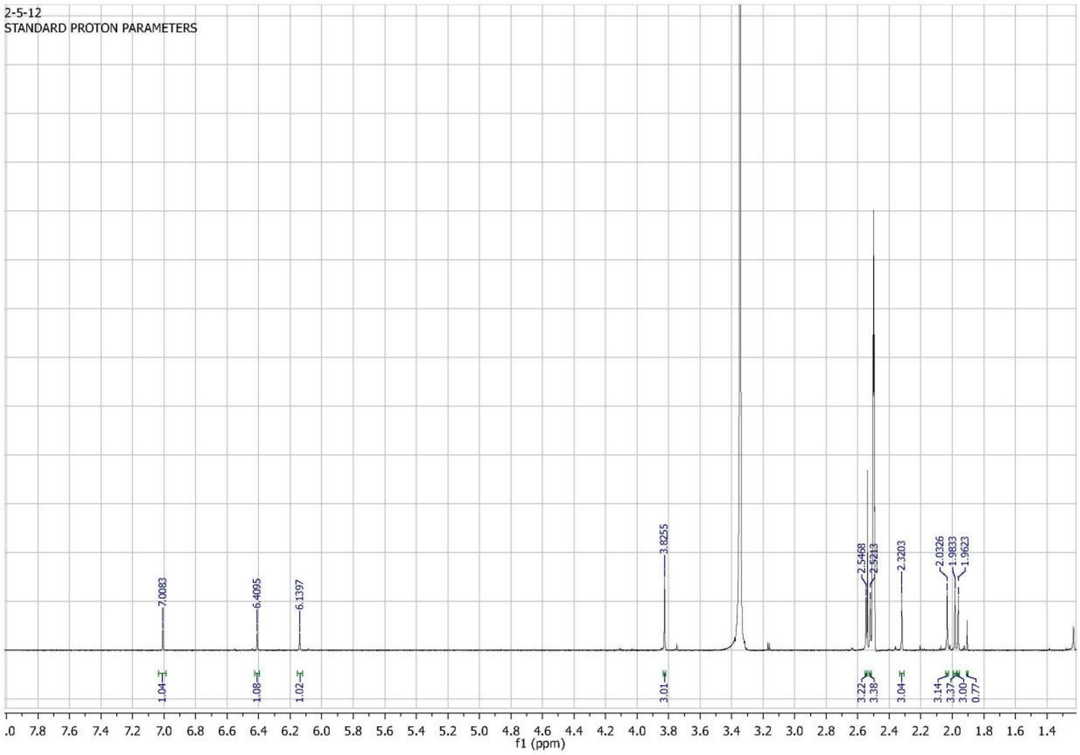

Figure S22 ^{1}H NMR spectrum of **4** in DMSO-d_6 (500 MHz)

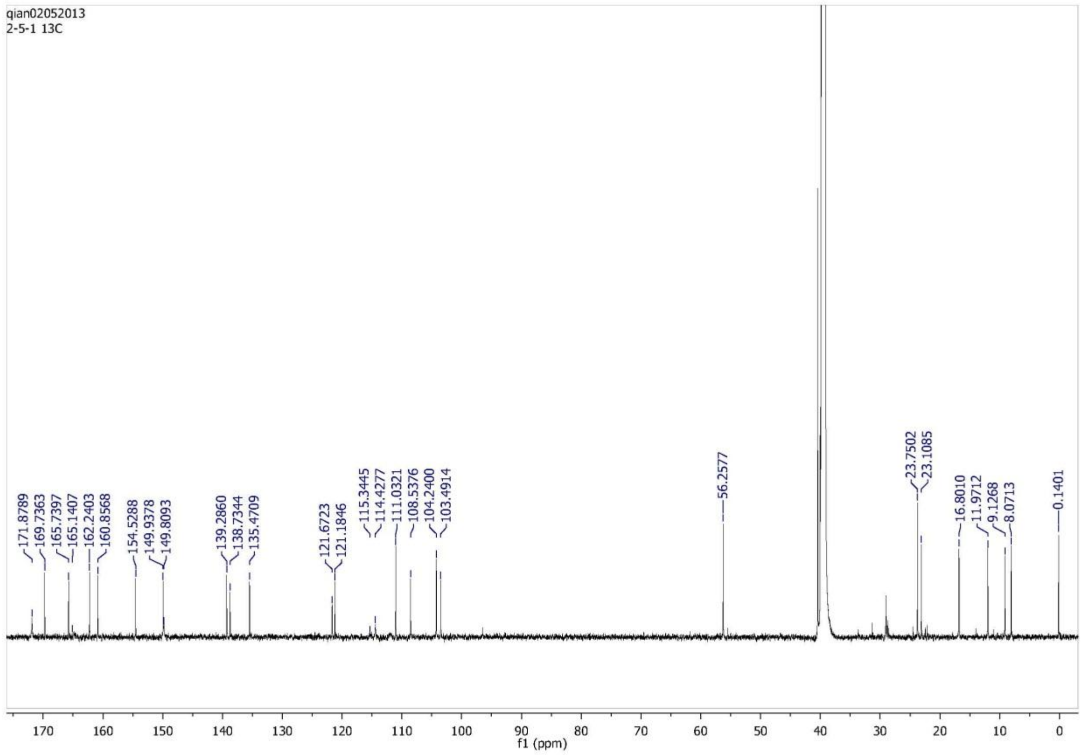

Figure S23 ^{13}C NMR spectrum of **4** in DMSO-d_6 (125 MHz)

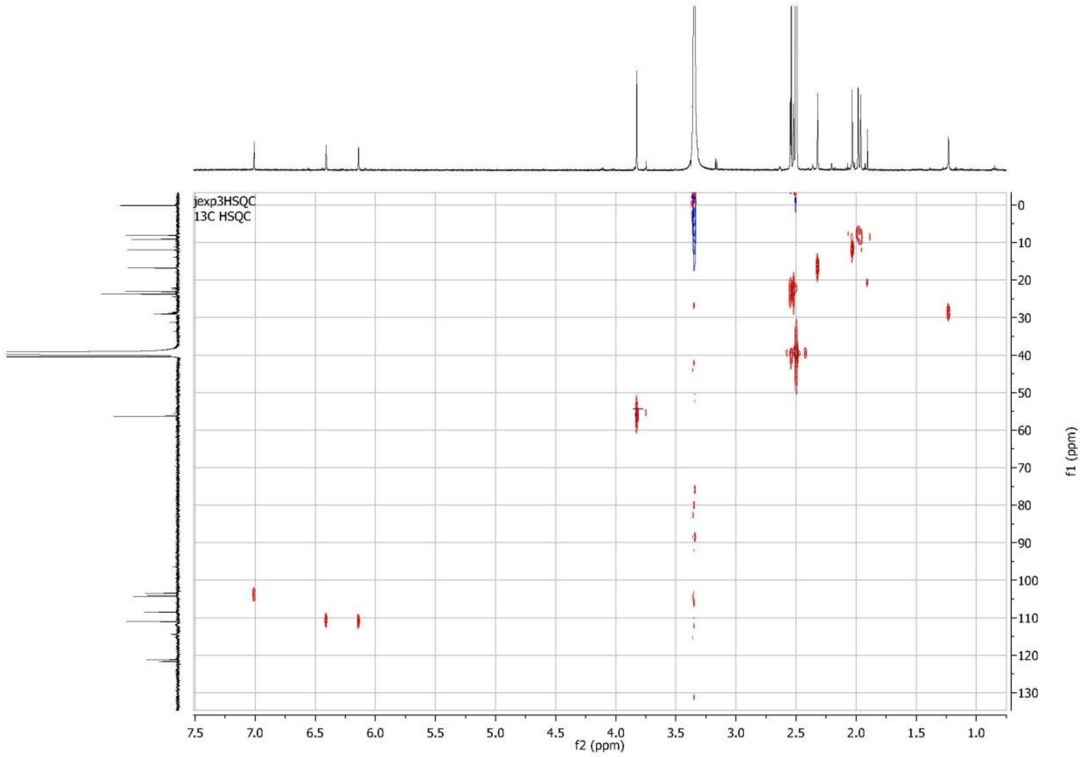

Figure S24 HMQC spectrum of **4** in DMSO-d_6

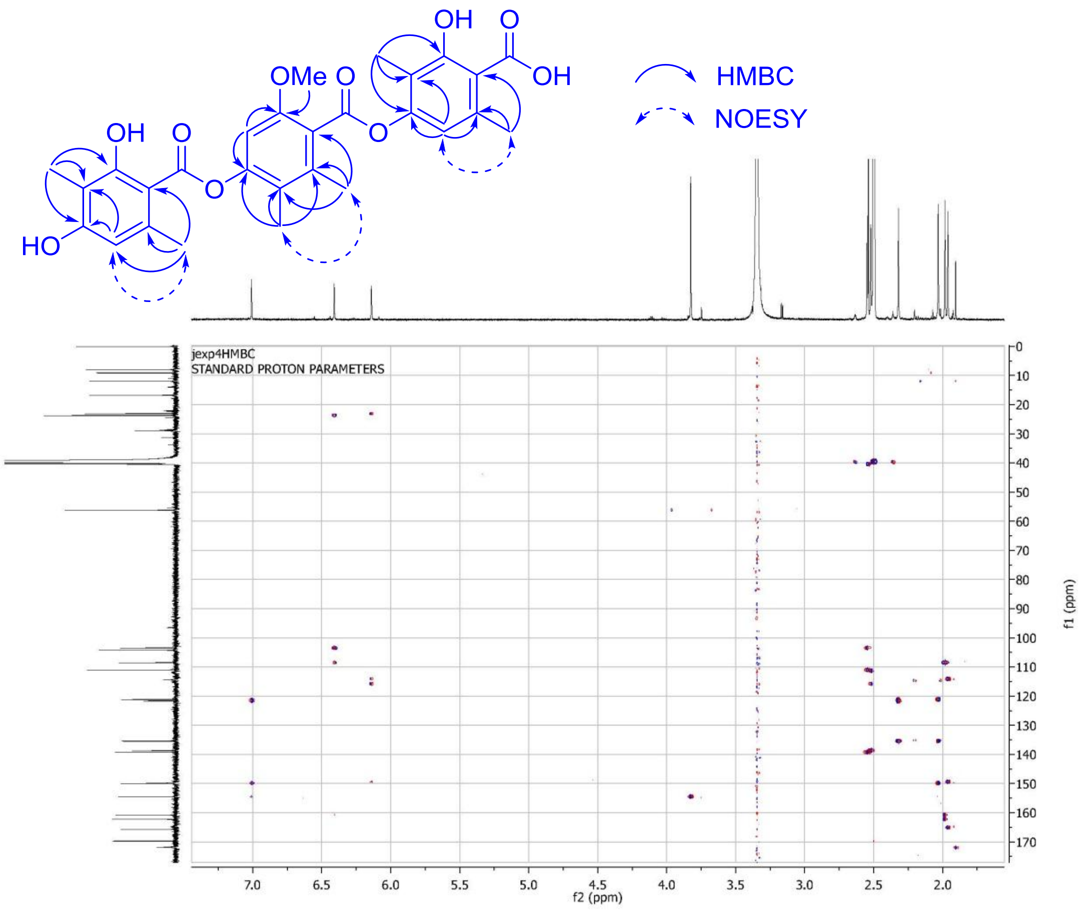

Figure S25 HMBC spectrum of **4** in DMSO-d_6

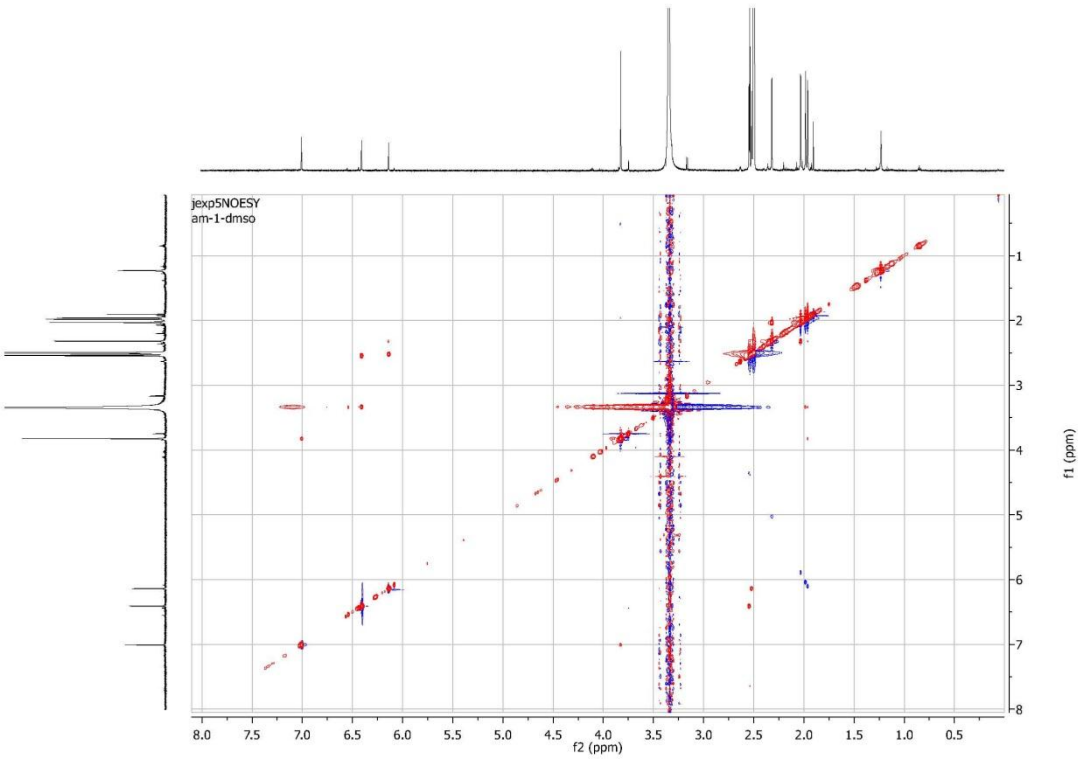

Figure S26 NOESY spectrum of **4** in DMSO-d_6

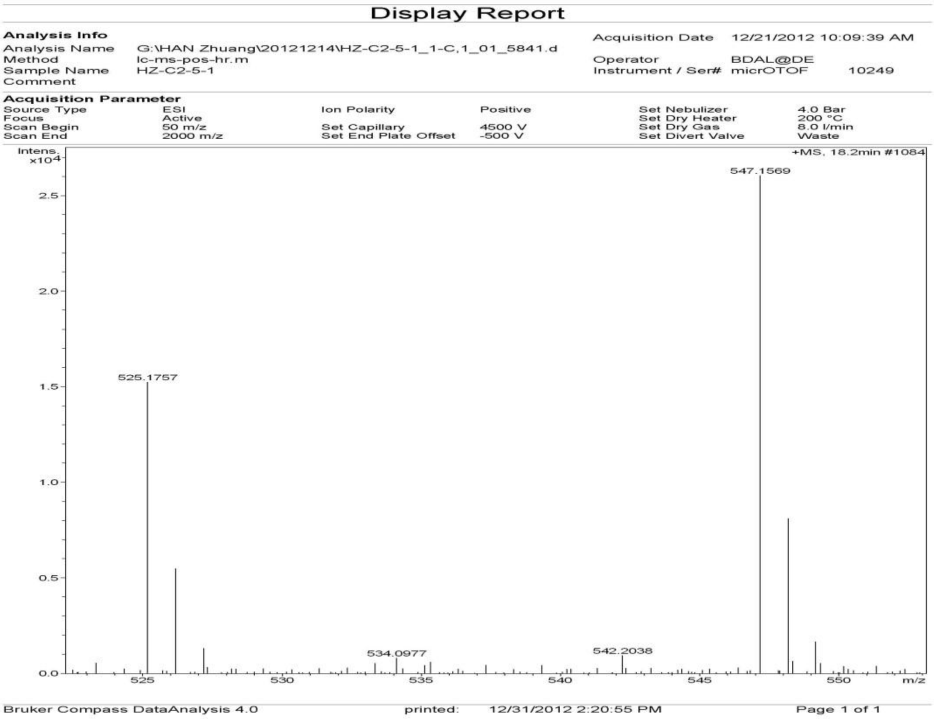

Figure S27 HRESIMS spectrum of **4**

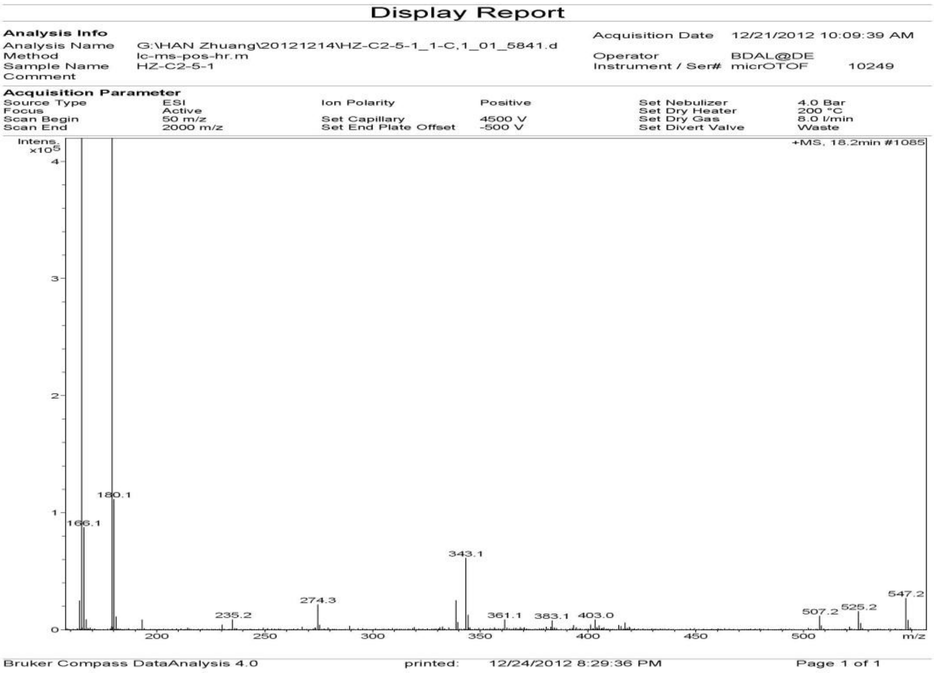

Figure S28 ISCID spectrum of **4**

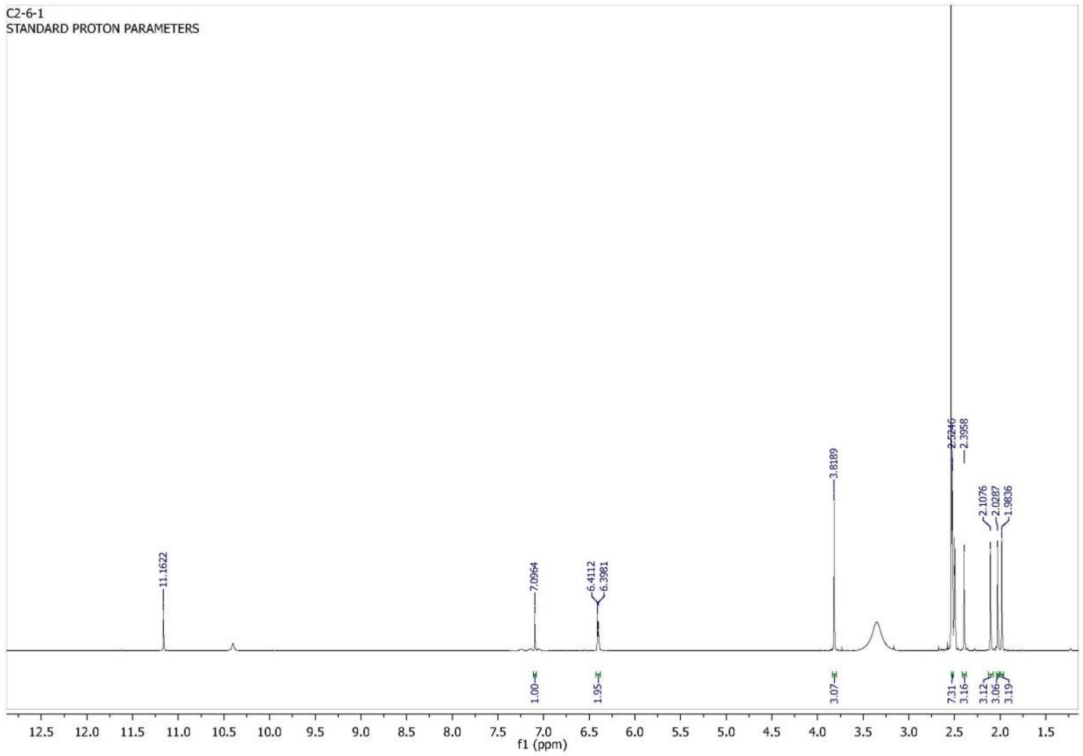

Figure S29 ^{1}H NMR spectrum of **5** in DMSO-d_6 (500 MHz)

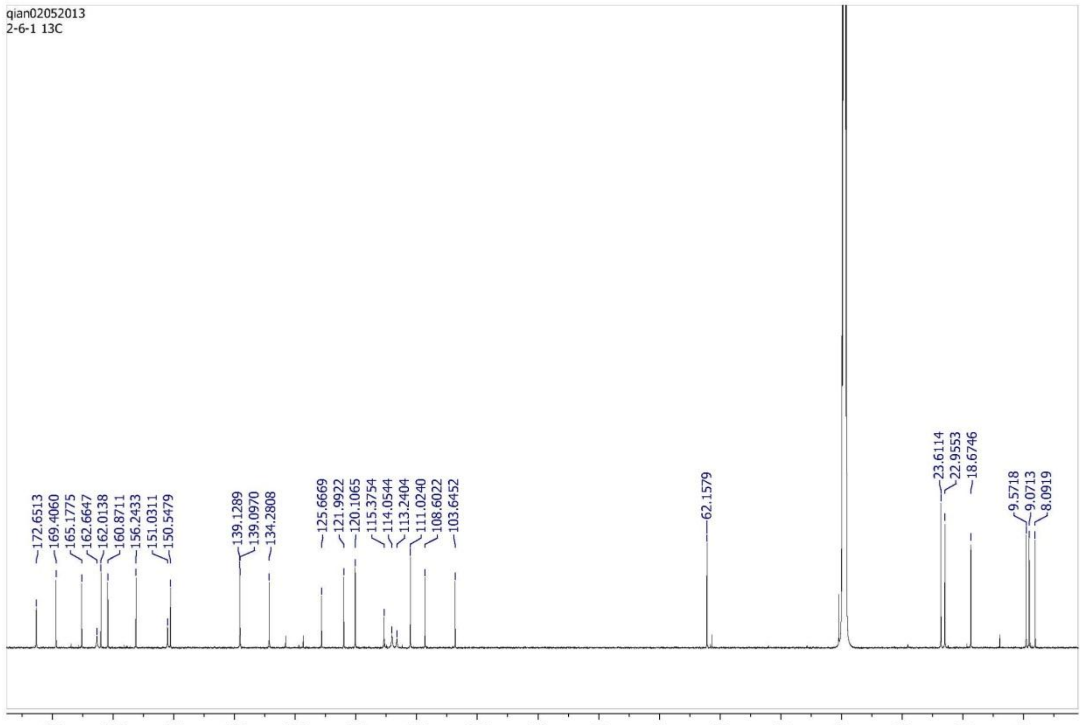

Figure S30 ^{13}C NMR spectrum of **5** in DMSO-d_6 (125 MHz)

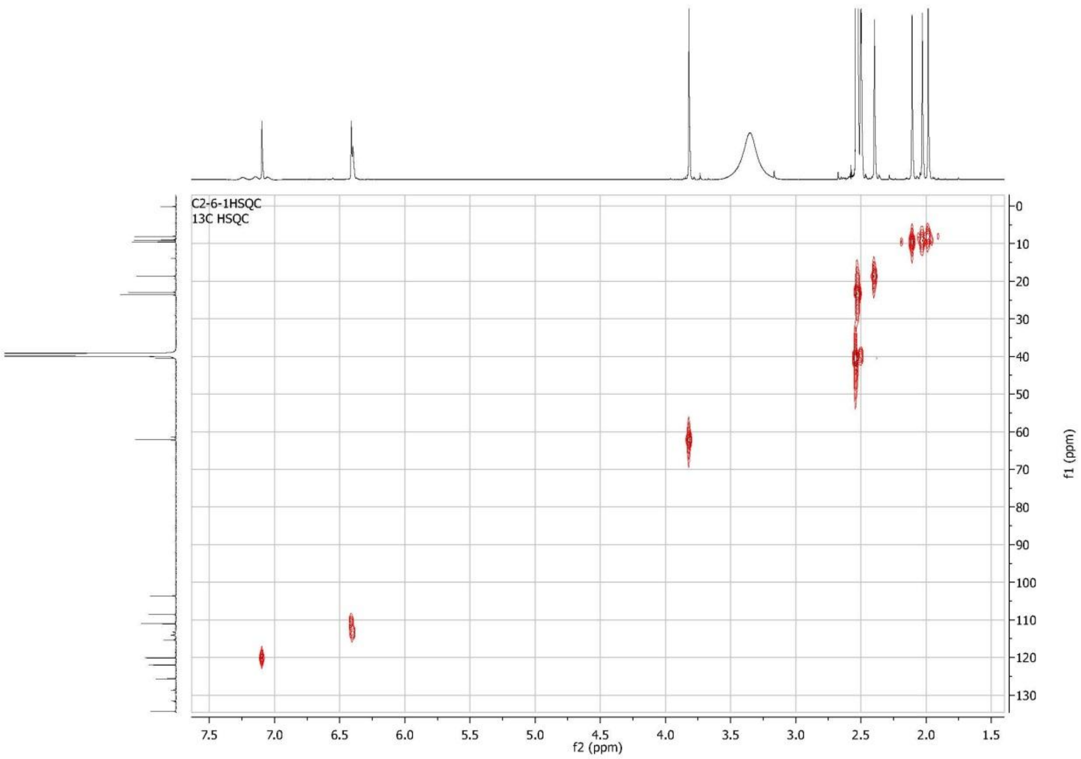

Figure S31 HMQC spectrum of **5** in DMSO-d_6

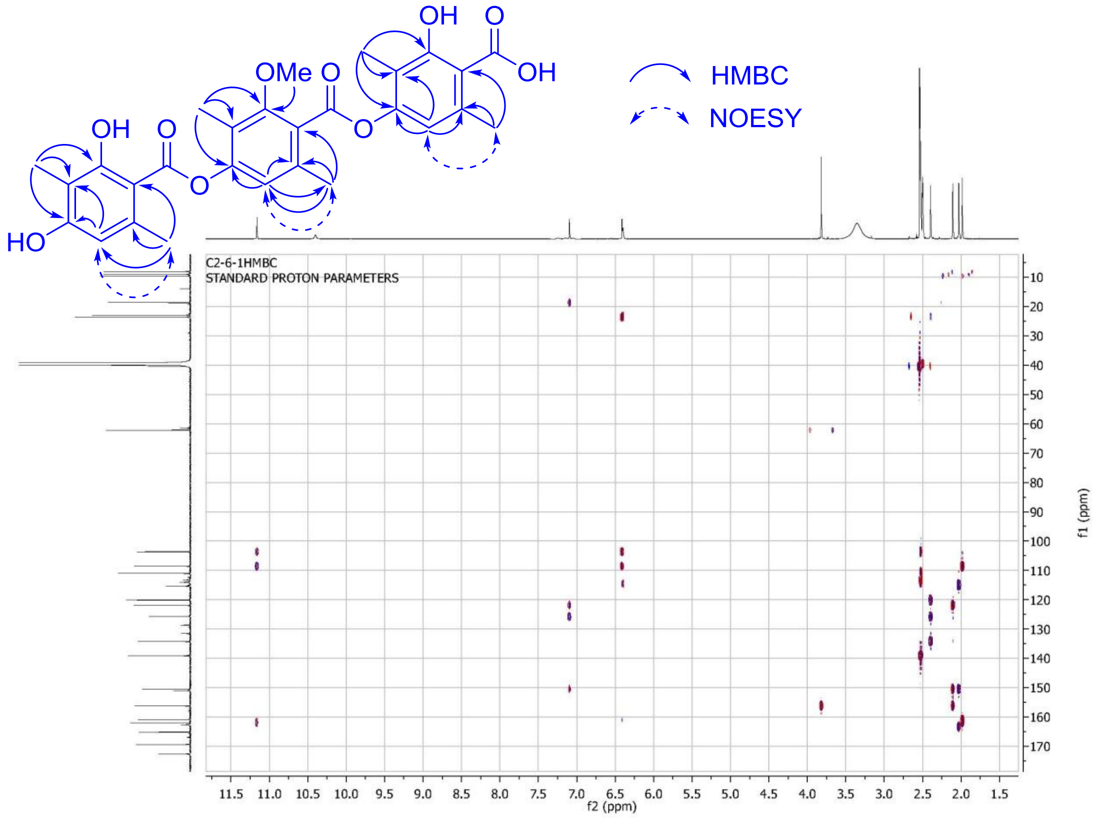

Figure S32 HMBC spectrum of **5** in DMSO-d_6

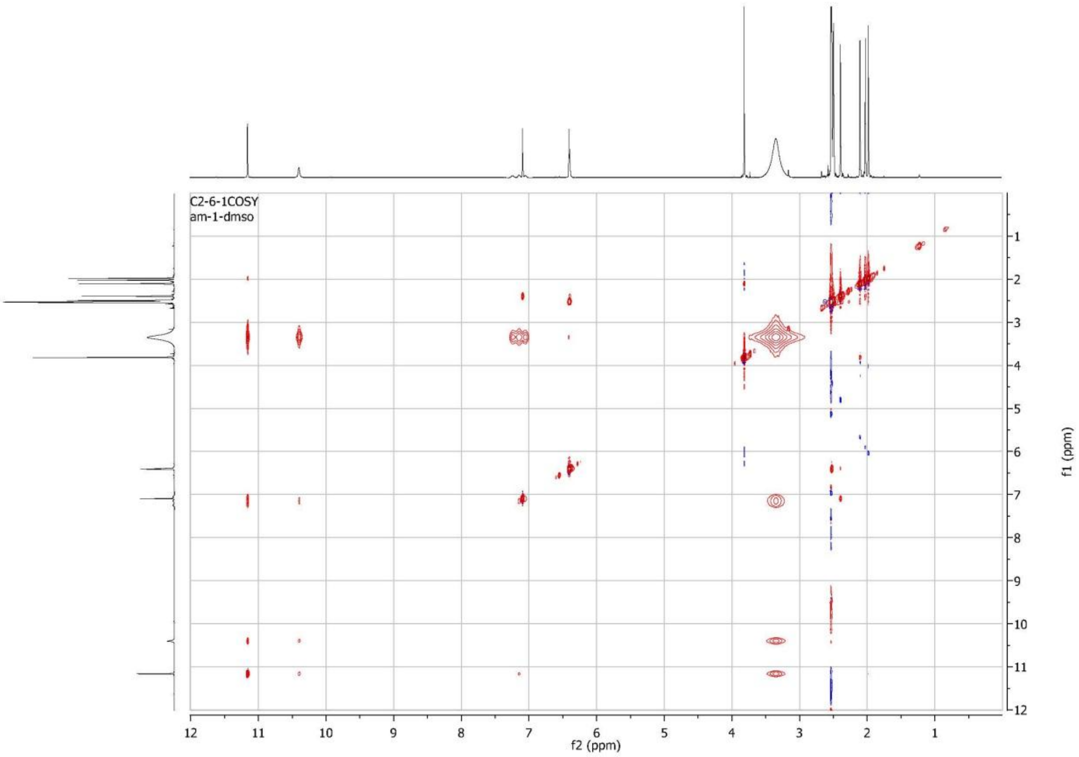

Figure S33 NOESY spectrum of **5** in DMSO-d_6

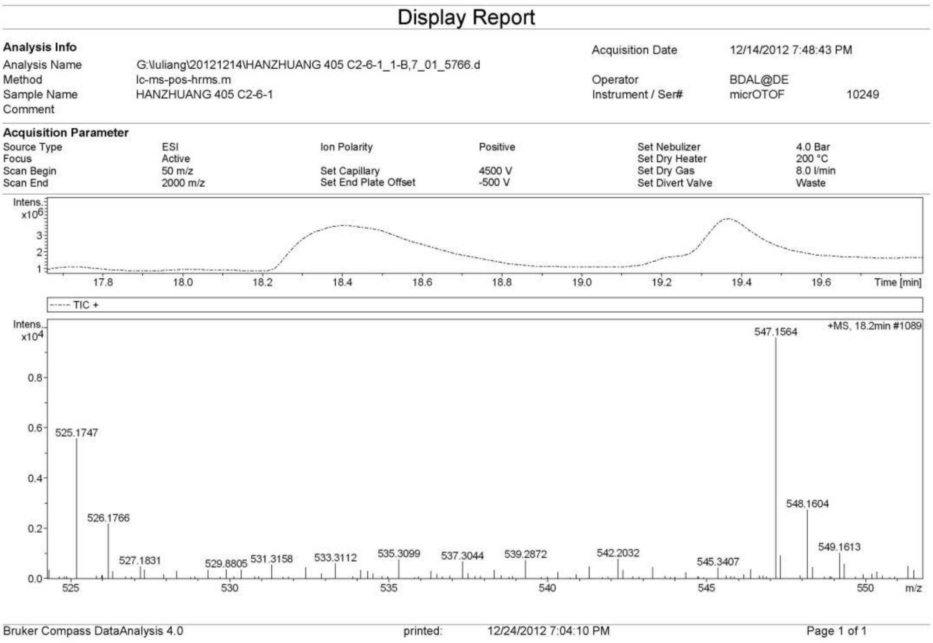

Figure S34 HRESIMS spectrum of **5**

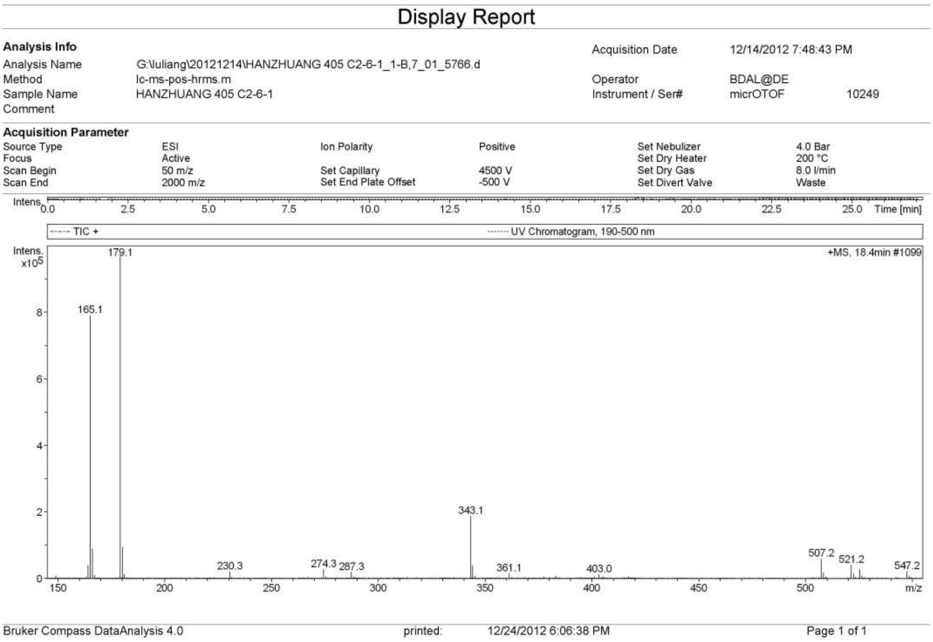

Figure S35 ISCID spectrum of **5**

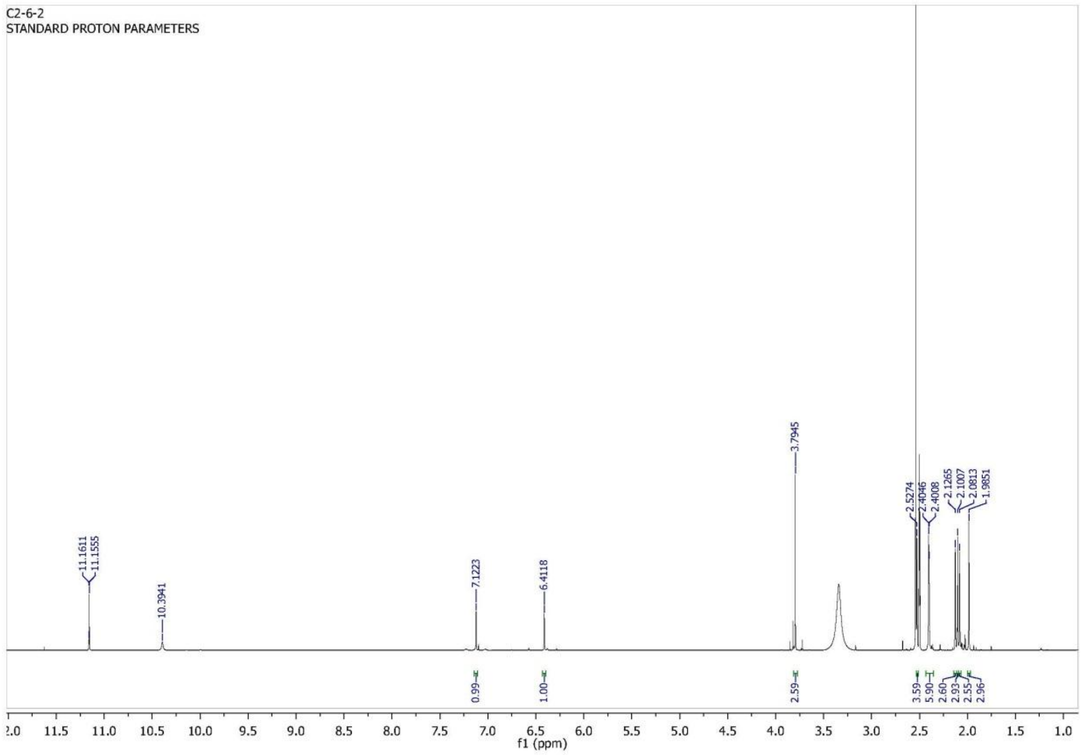

Figure S36 ^{1}H NMR spectrum of **6** in DMSO-d_6 (500 MHz)

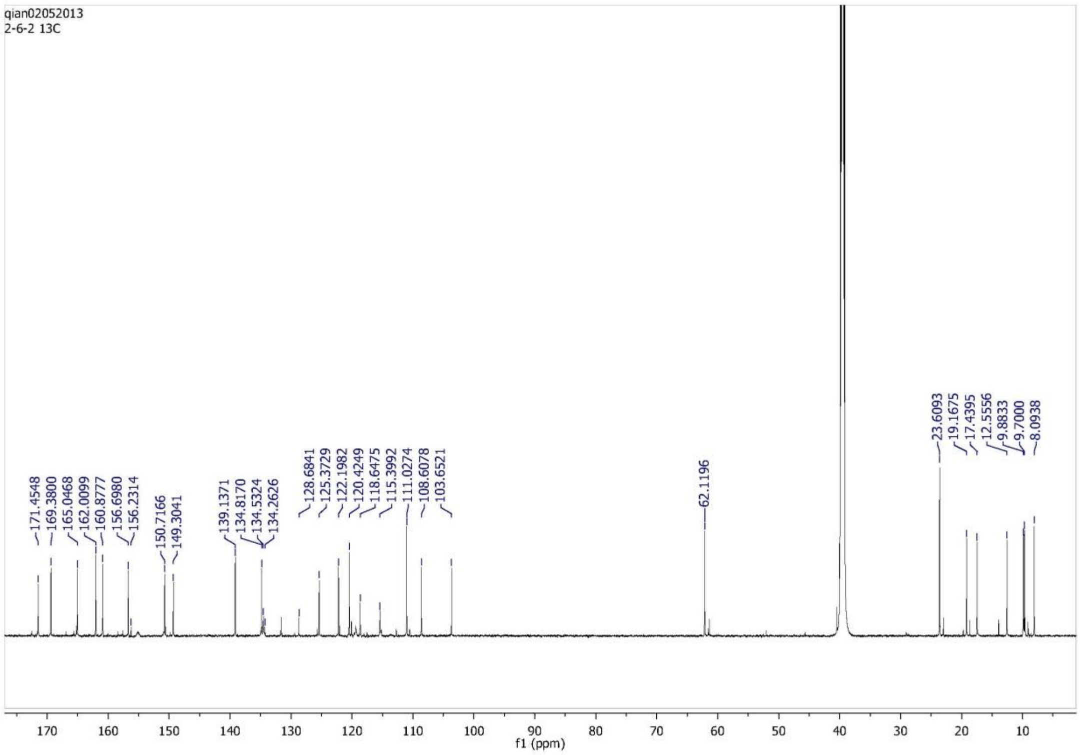

Figure S37 ^{13}C NMR spectrum of **6** in DMSO-d_6 (125 MHz)

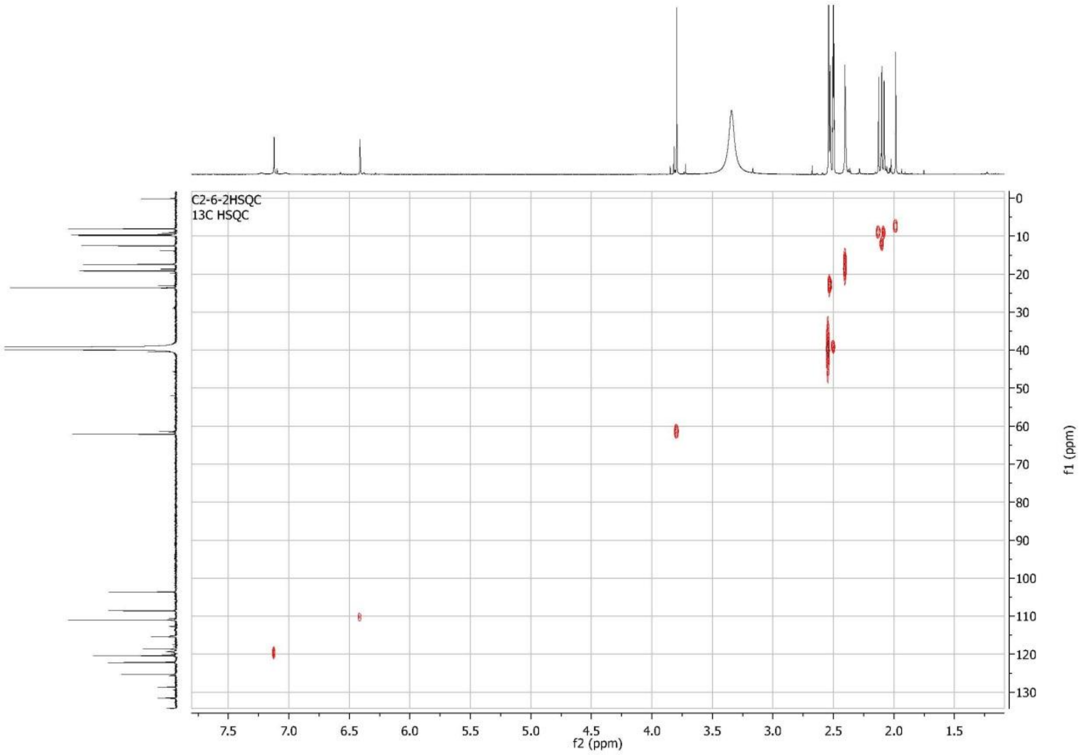

Figure S38 HMQC spectrum of **6** in DMSO-d_6

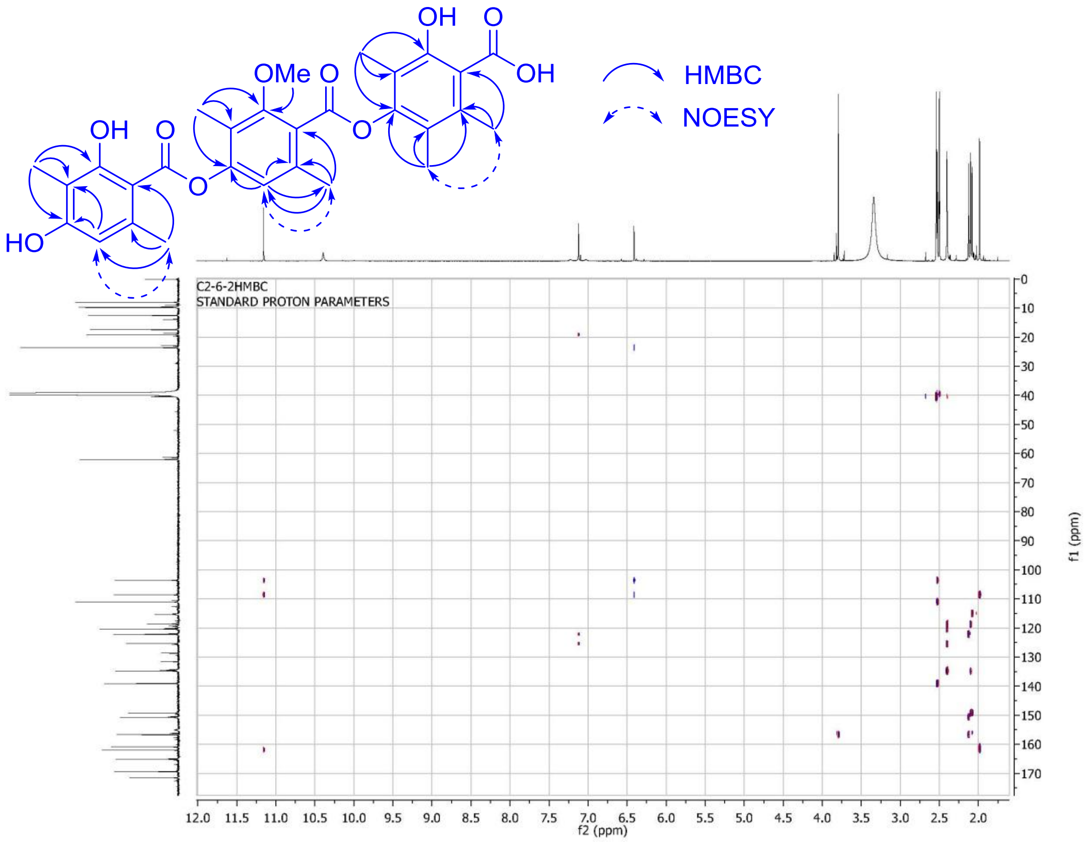

Figure S39 HMBC spectrum of **6** in DMSO-d_6

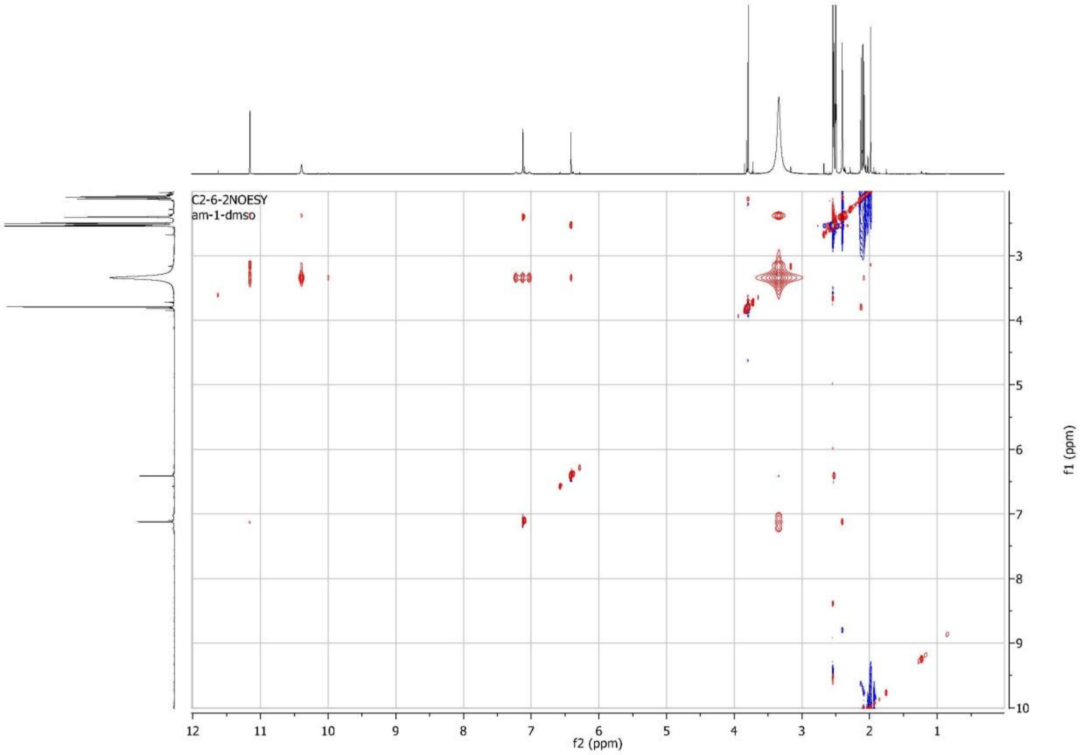

Figure S40 NOESY spectrum of **6** in DMSO-d_6

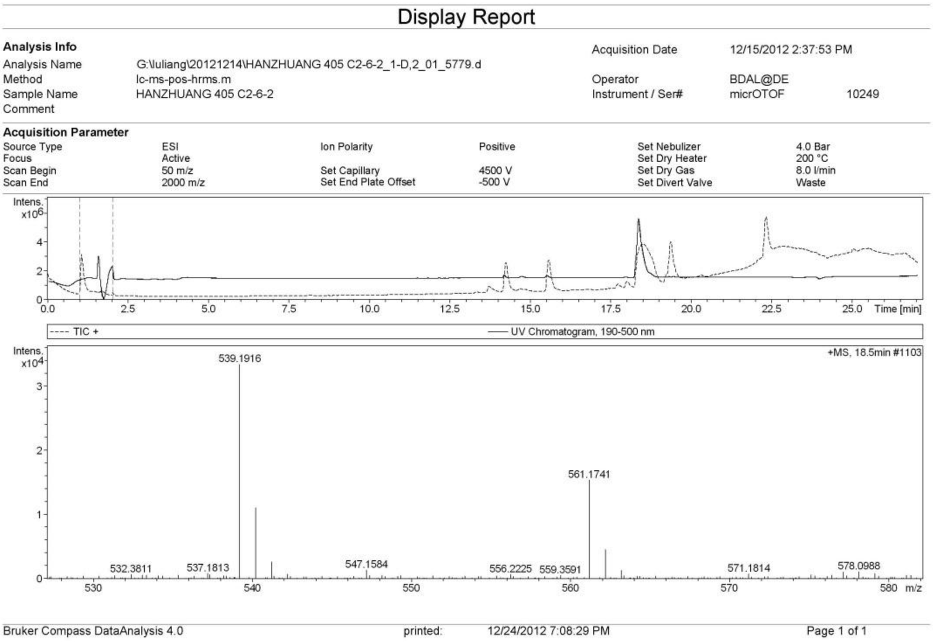

Figure S41 HRESIMS spectrum of **6**

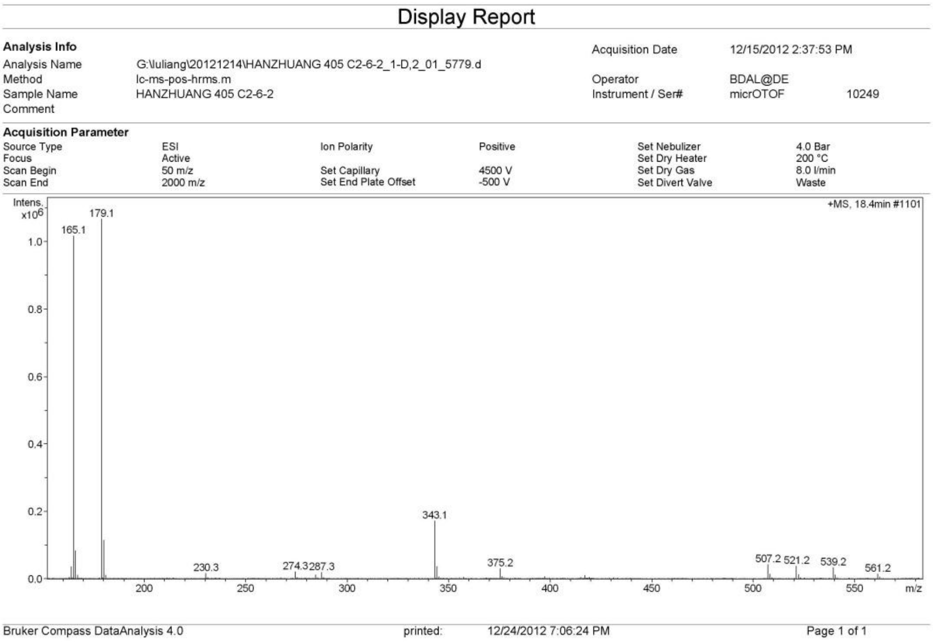

Figure S42 ISCID spectrum of **6**

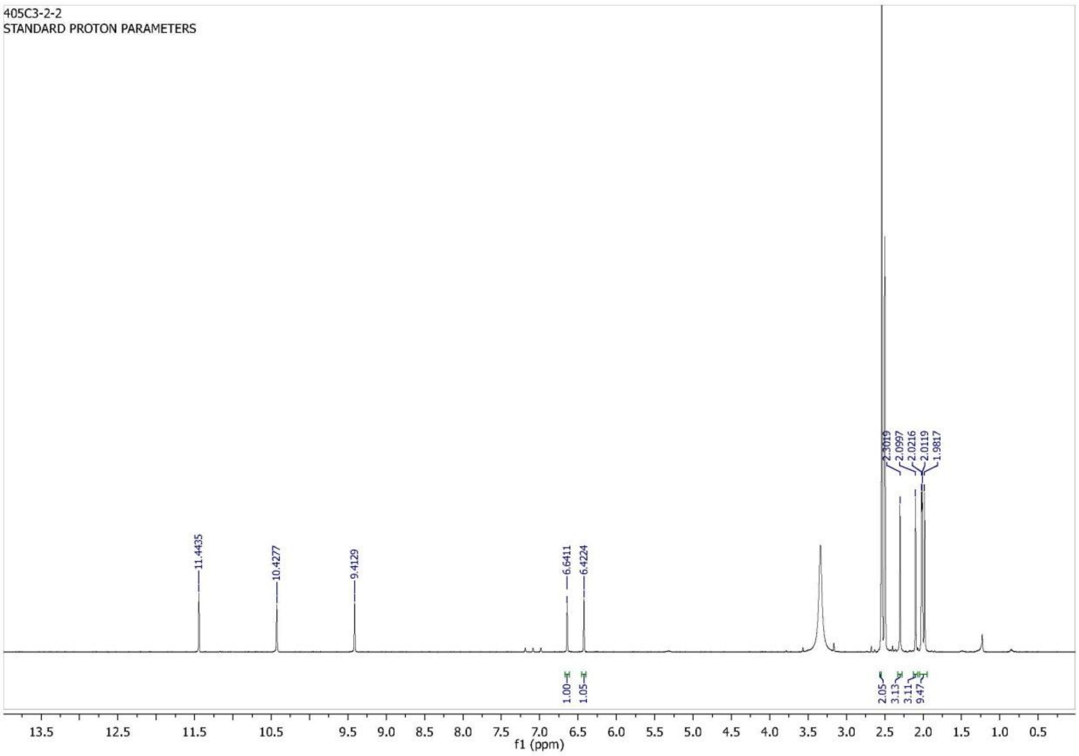

Figure S43 ^{1}H NMR spectrum of **7** in DMSO-d_6 (500 MHz)

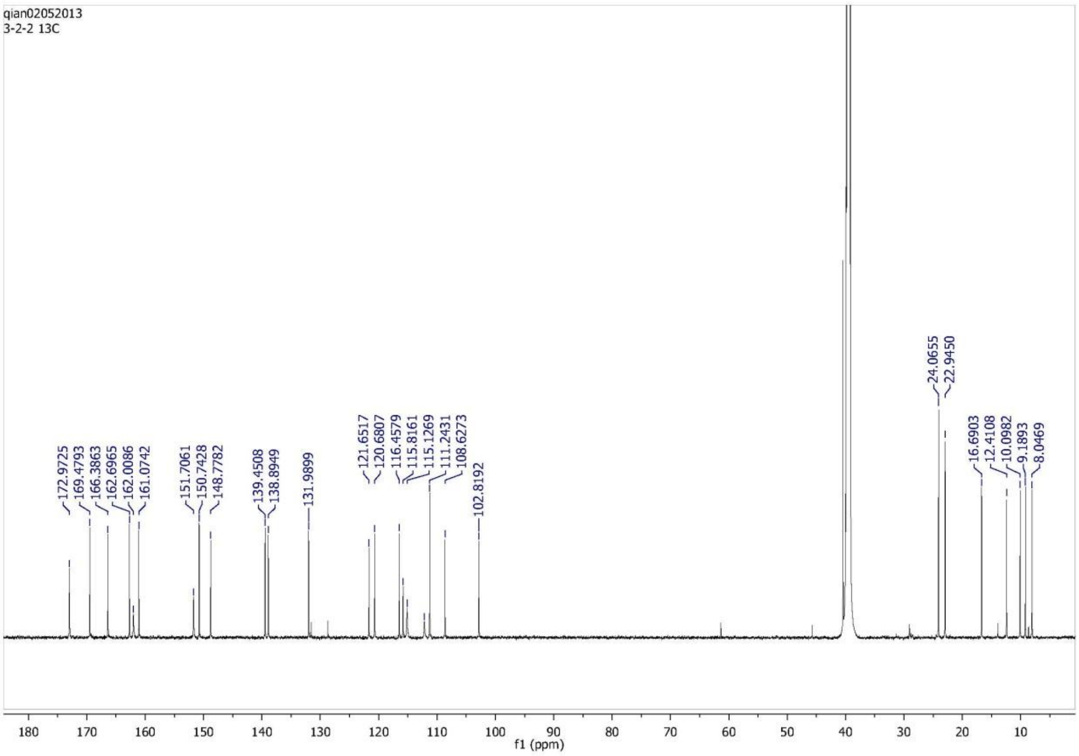

Figure S44 ^{13}C NMR spectrum of **7** in DMSO-d_6 (125 MHz)

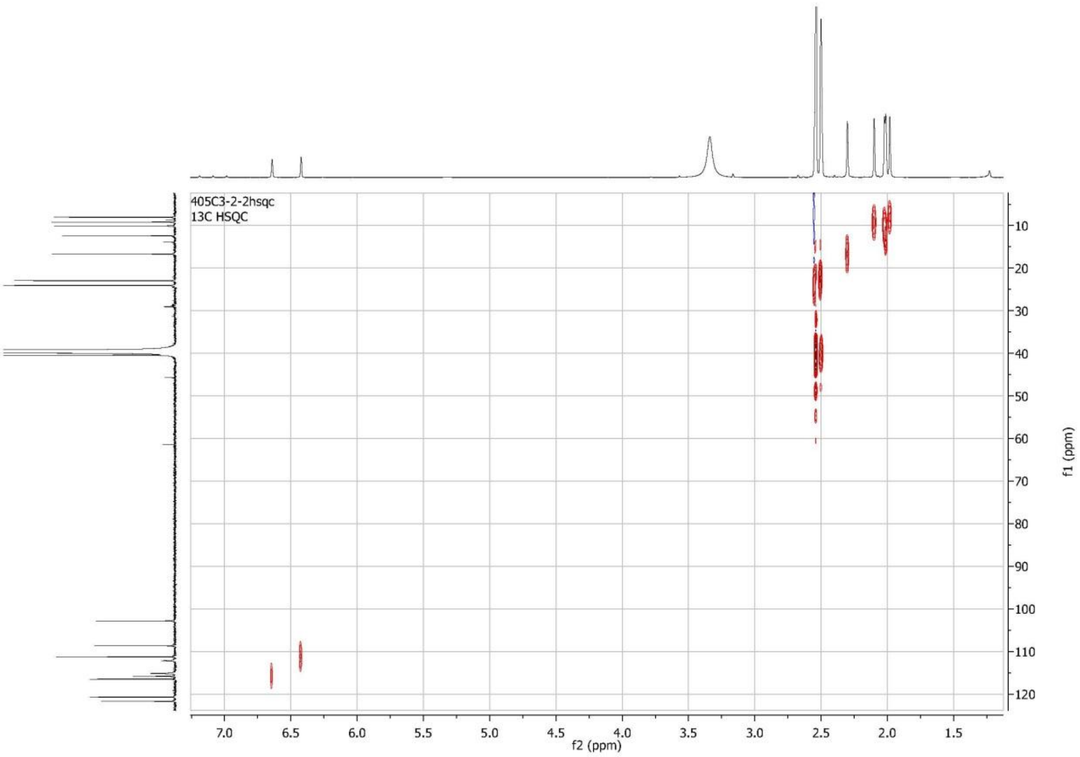

Figure S45 HMQC spectrum of **7** in DMSO-d_6

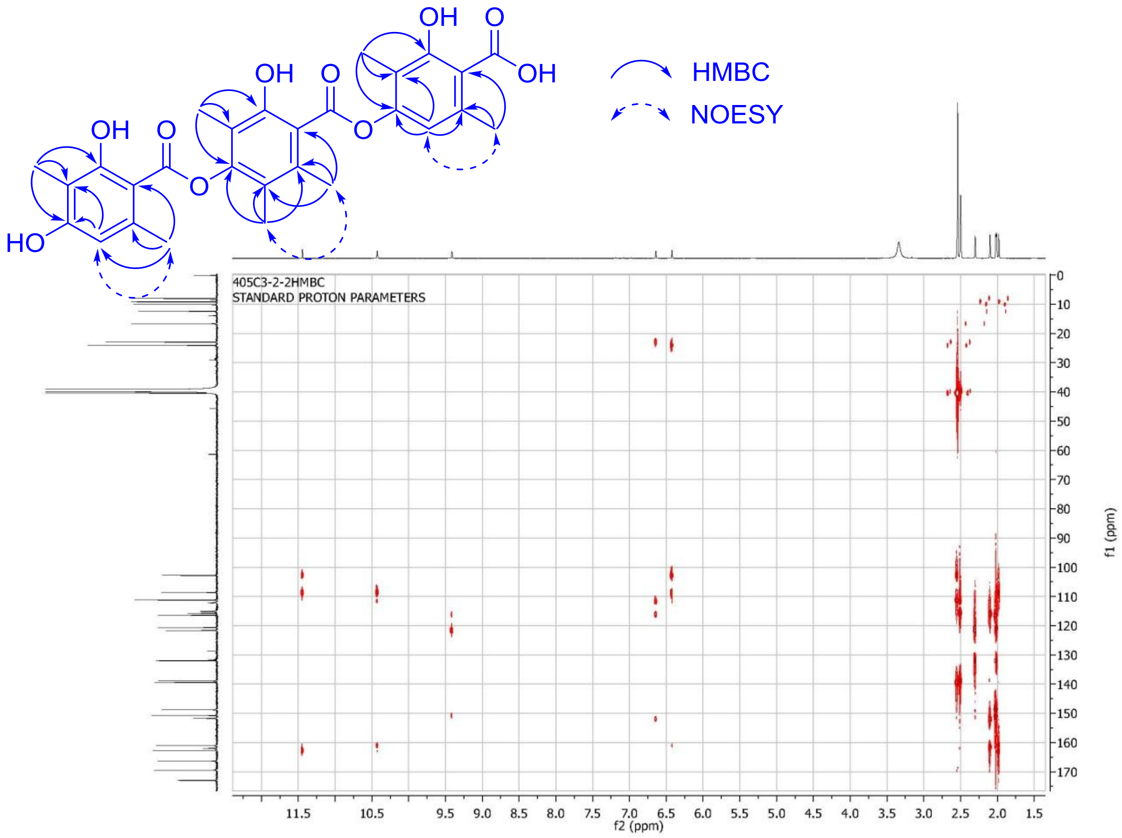

Figure S46 HMBC spectrum of **7** in DMSO-d_6

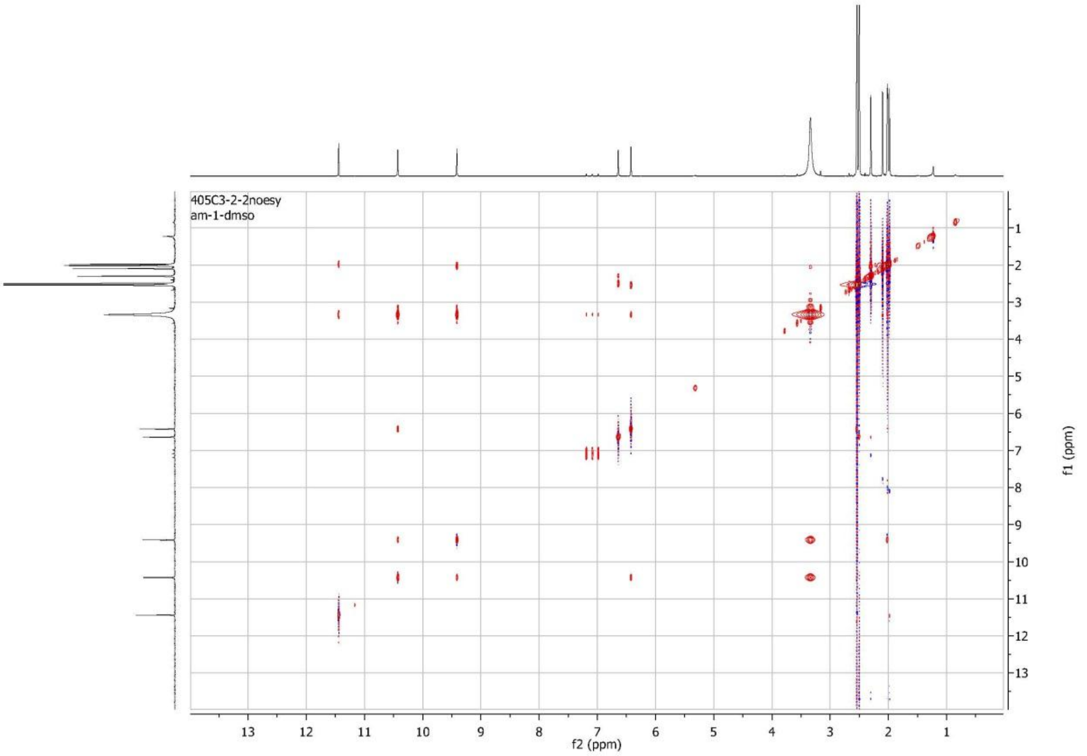

Figure S47 NOESY spectrum of **7** in DMSO-d_6

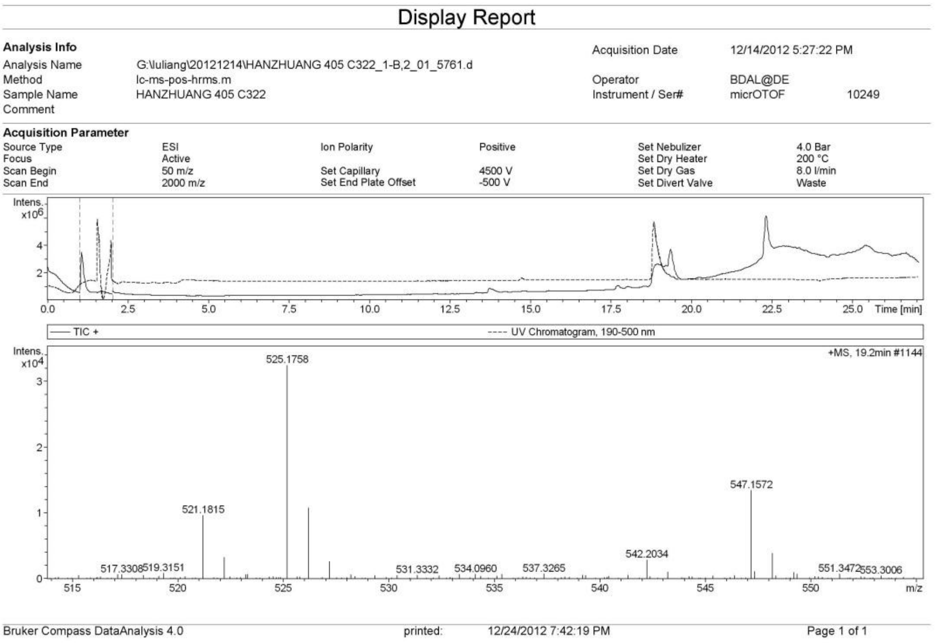

Figure S48 HRESIMS spectrum of **7**

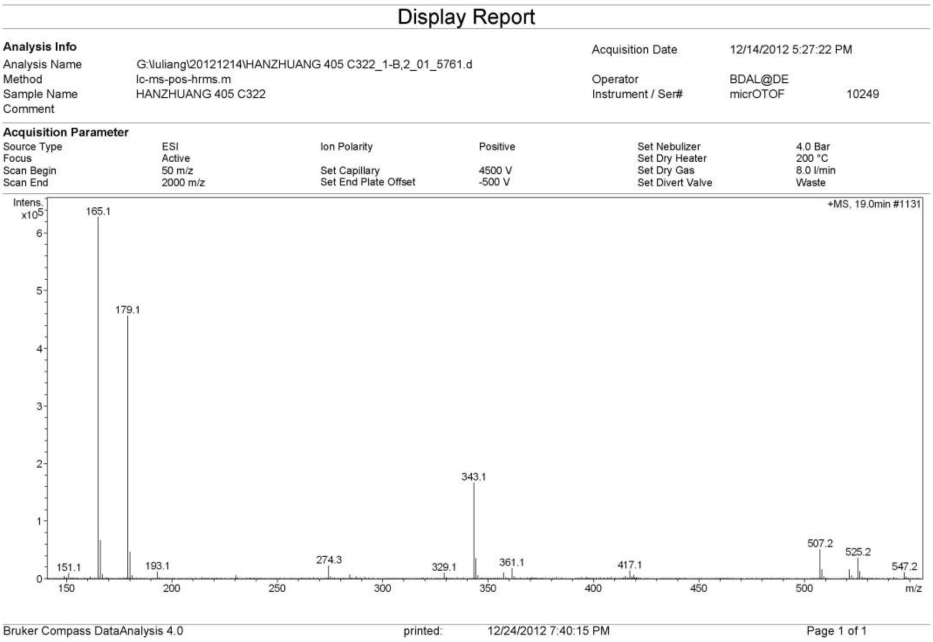

Figure S49 ISCID spectrum of **7**

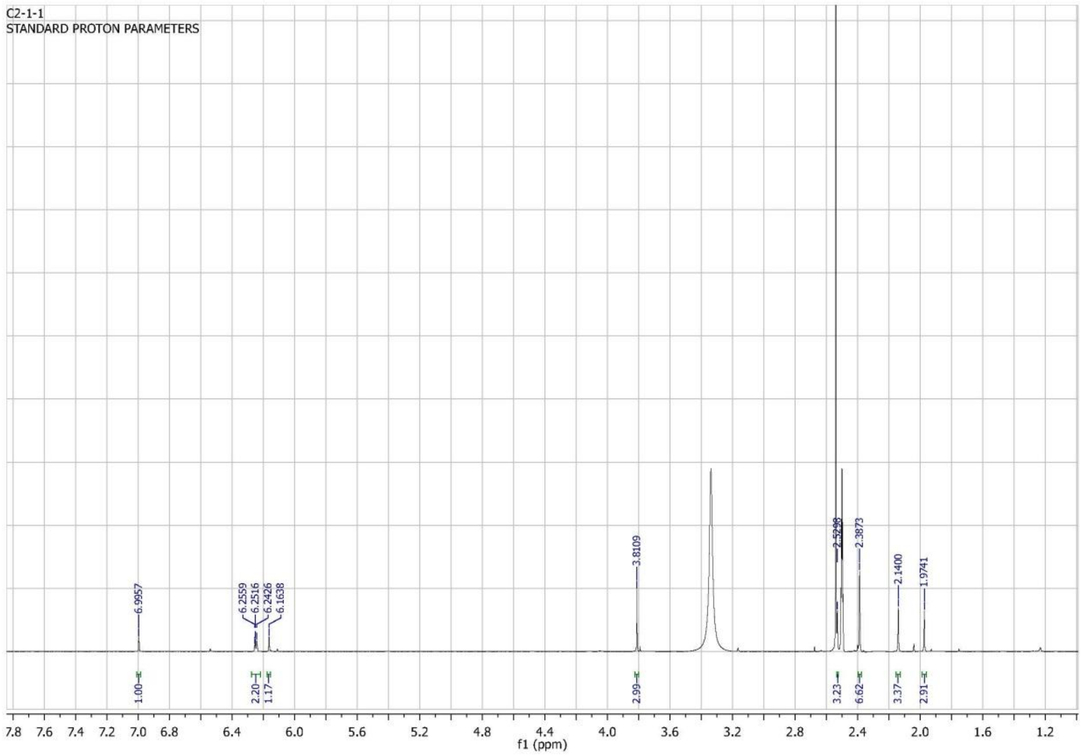

Figure S50 ^{1}H NMR spectrum of **8** in DMSO-d_6 (500 MHz)

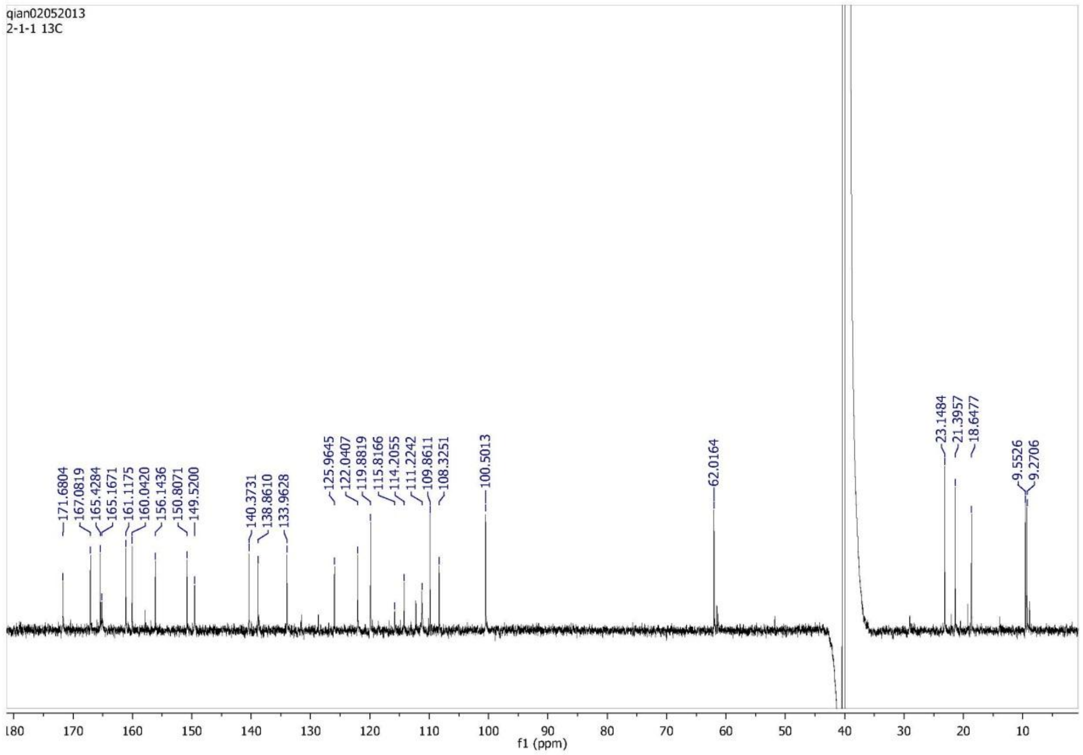

Figure S51 ^{13}C NMR spectrum of **8** in DMSO-d_6 (125 MHz)

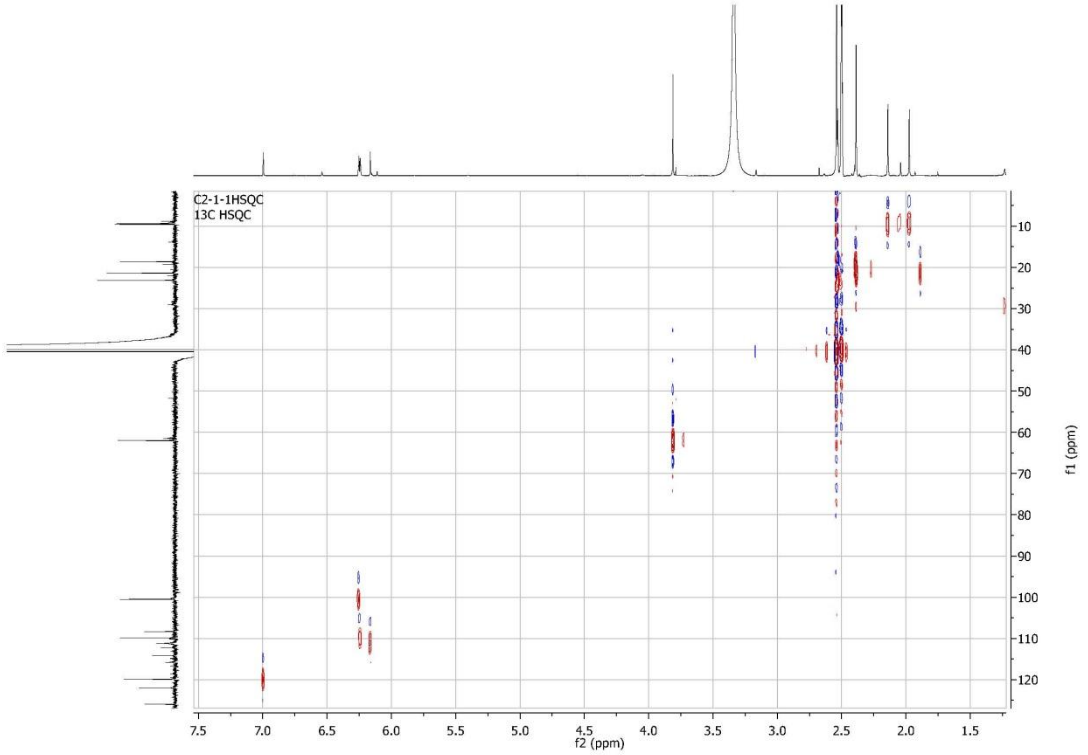

Figure S52 HMQC spectrum of **8** in DMSO-d_6

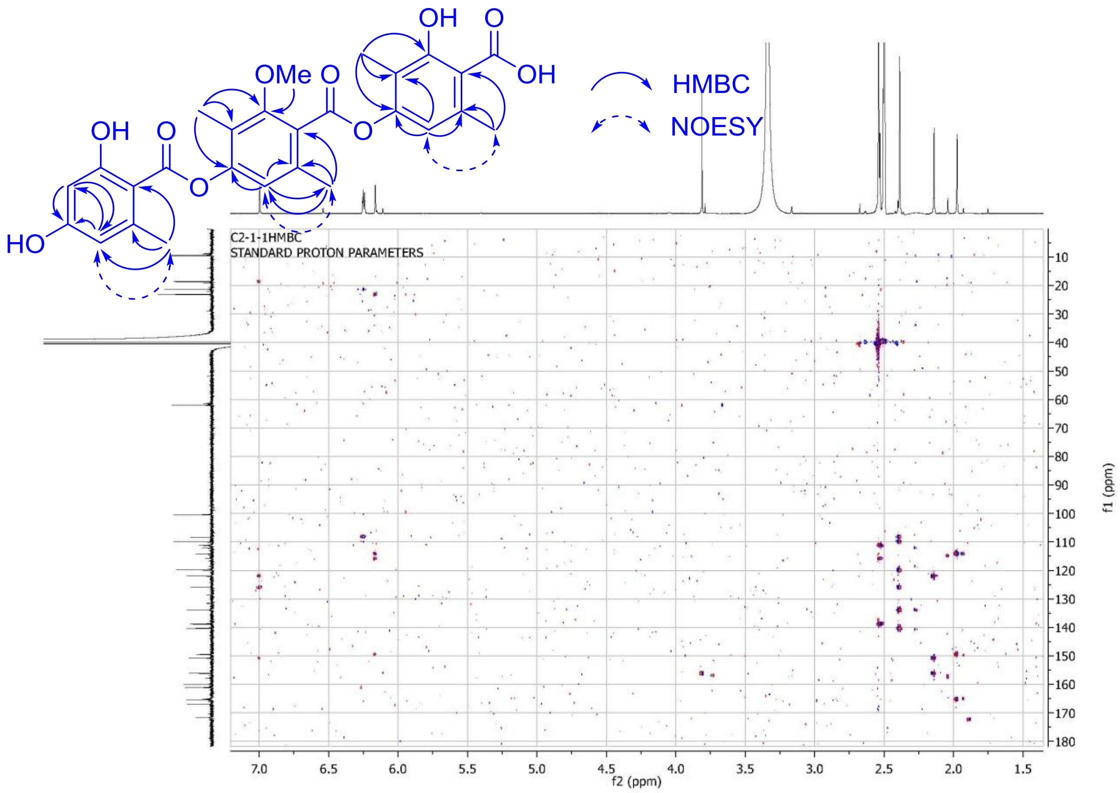

Figure S53 HMBC spectrum of **8** in DMSO-d_6

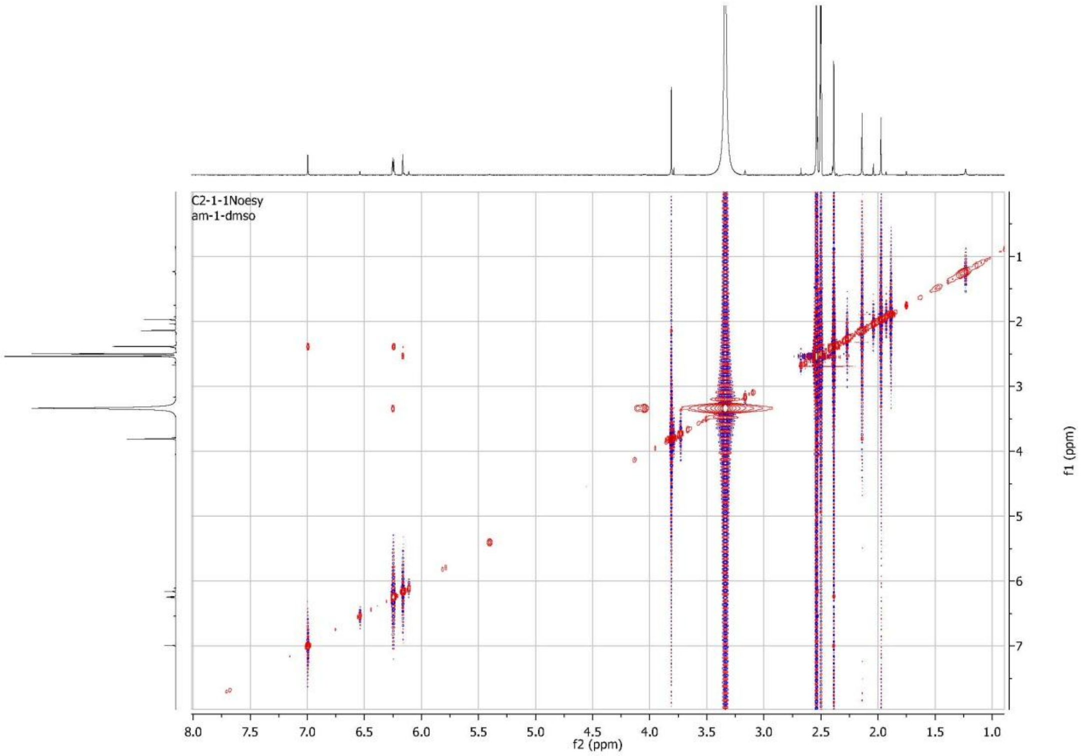

Figure S54 NOESY spectrum of **8** in DMSO-d_6

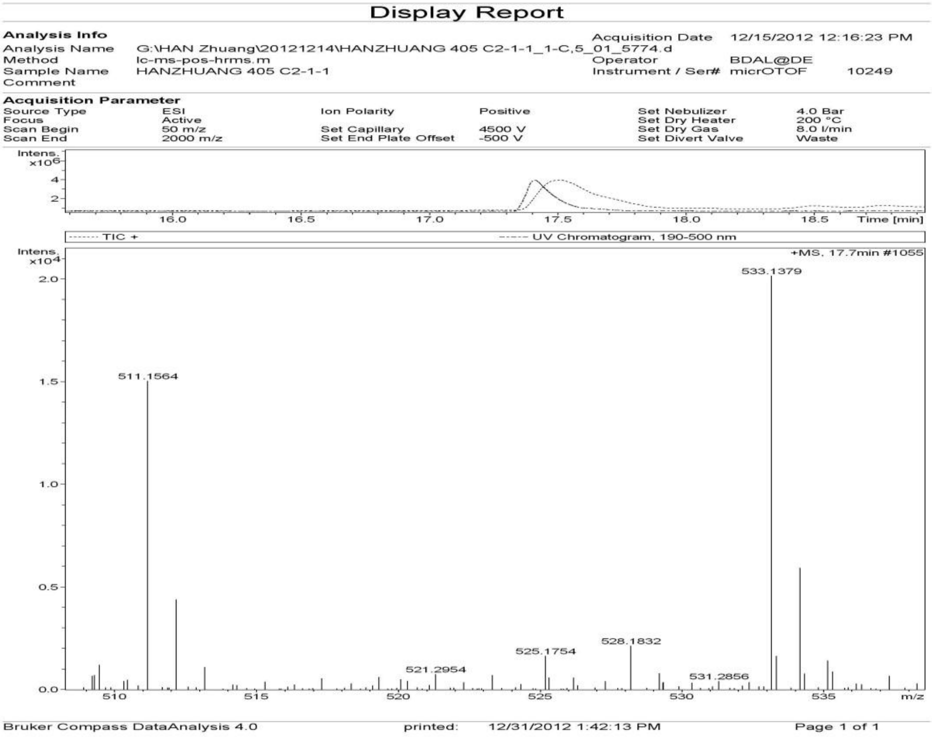

Figure S55 HRESIMS spectrum of **8**

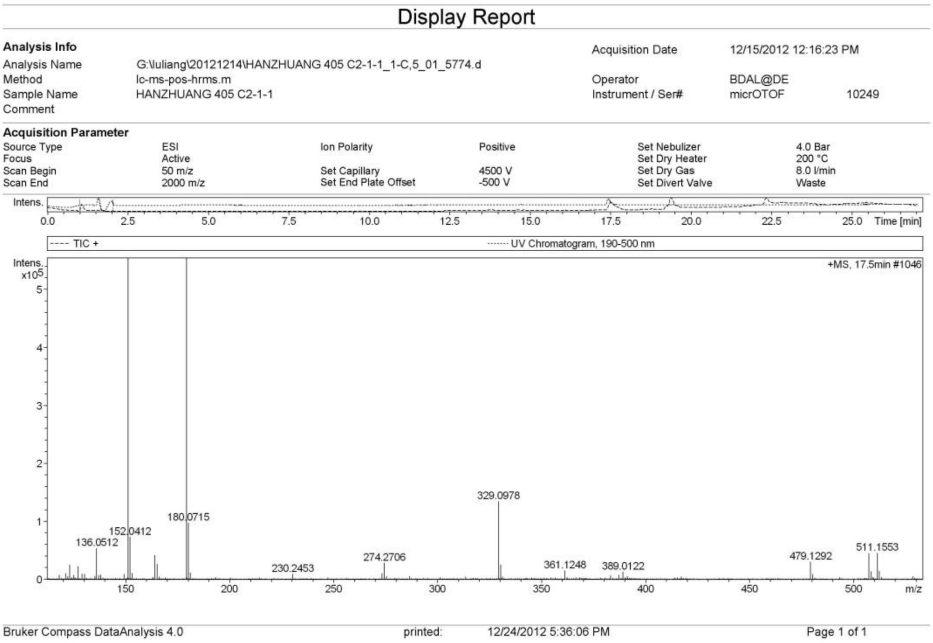

Figure S56 ISCID spectrum of **8**

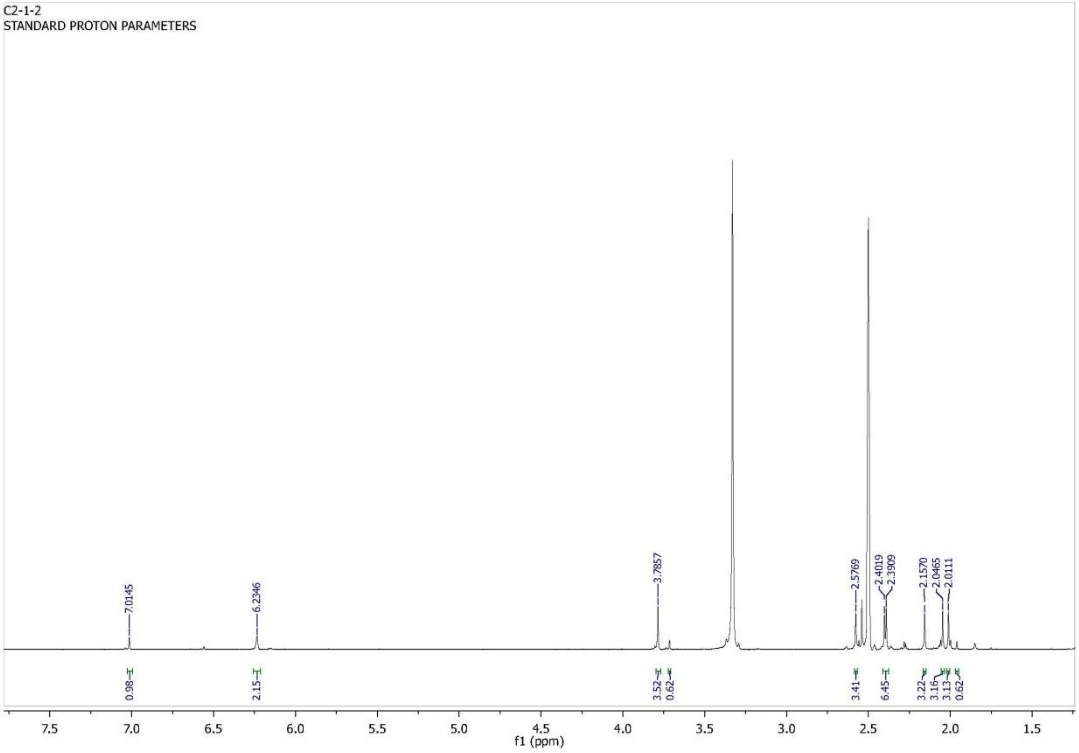

Figure S57 ^{1}H NMR spectrum of **9** in DMSO-d_6 (500 MHz)

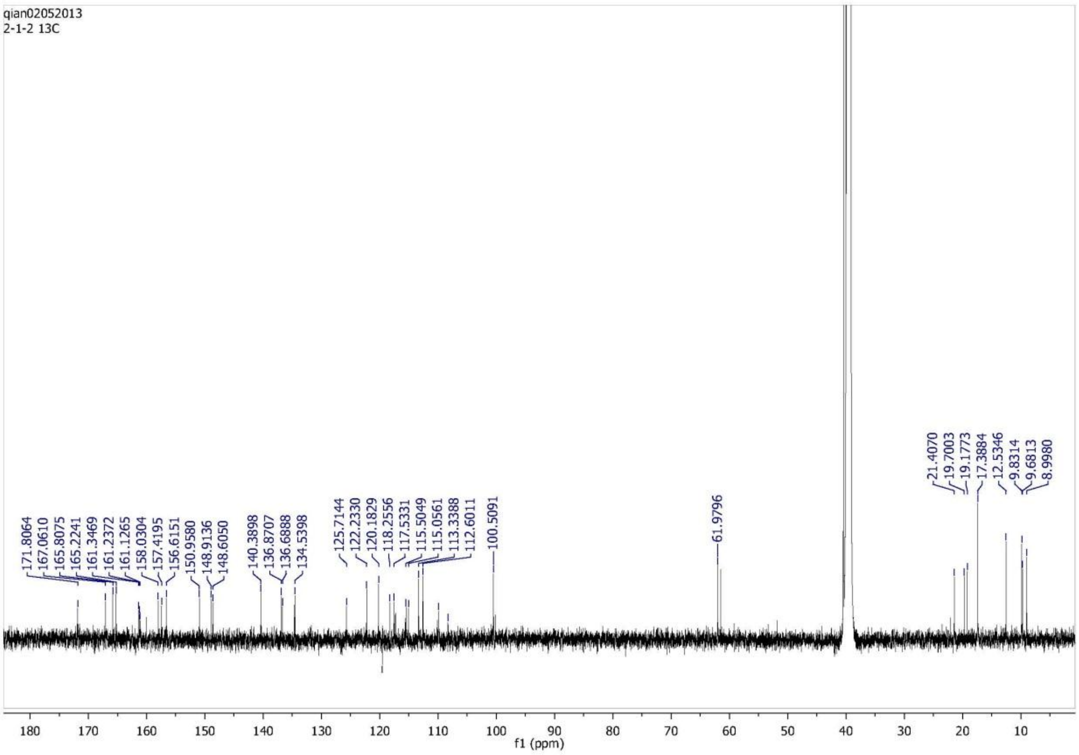

Figure S58 ^{13}C NMR spectrum of **9** in DMSO-d_6 (125 MHz)

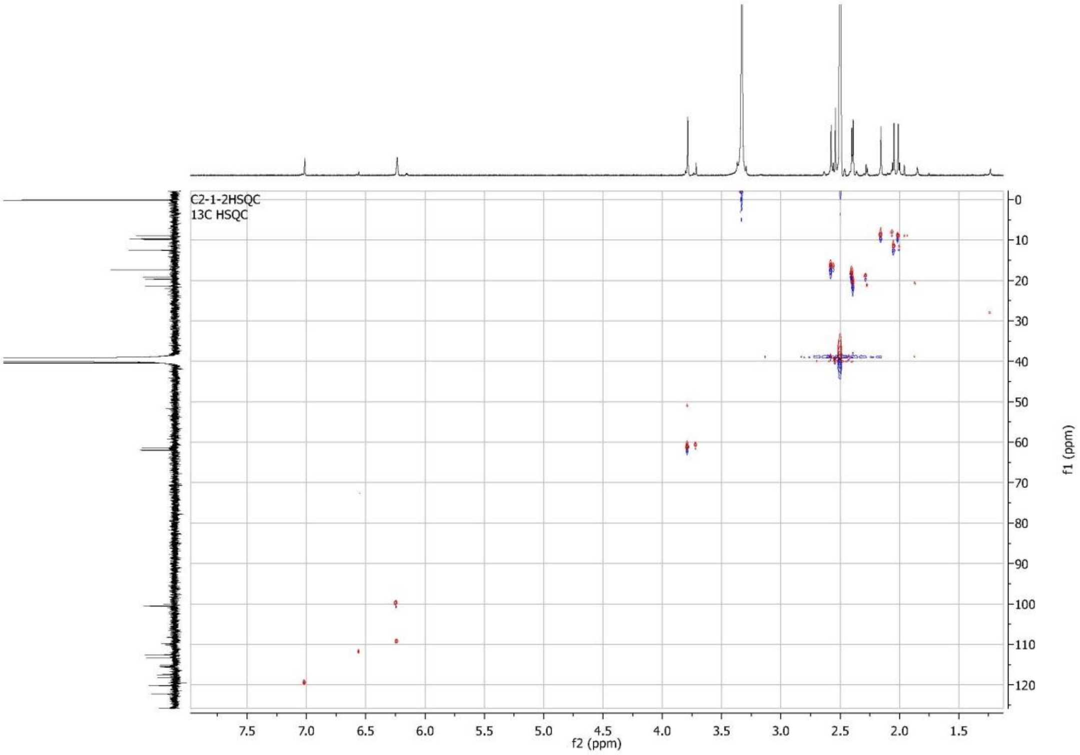

Figure S59 HMQC spectrum of **9** in DMSO-d_6

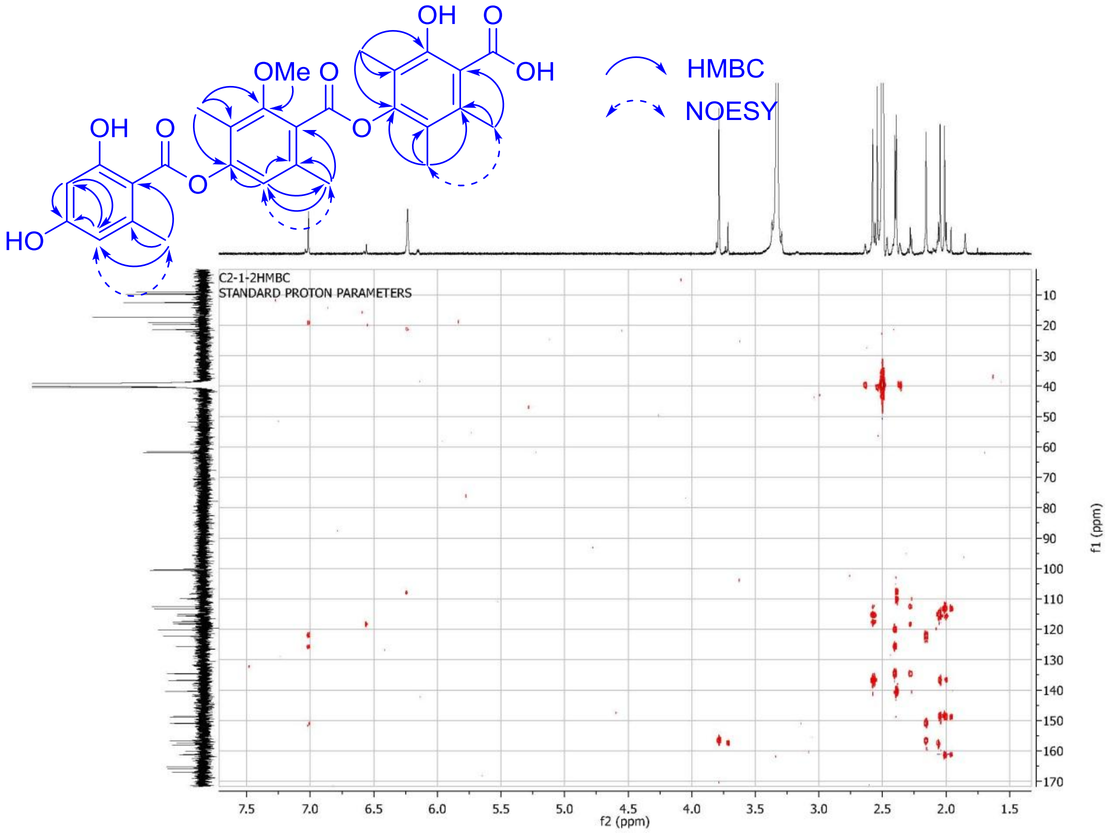

Figure S60 HMBC spectrum of **9** in DMSO-d_6

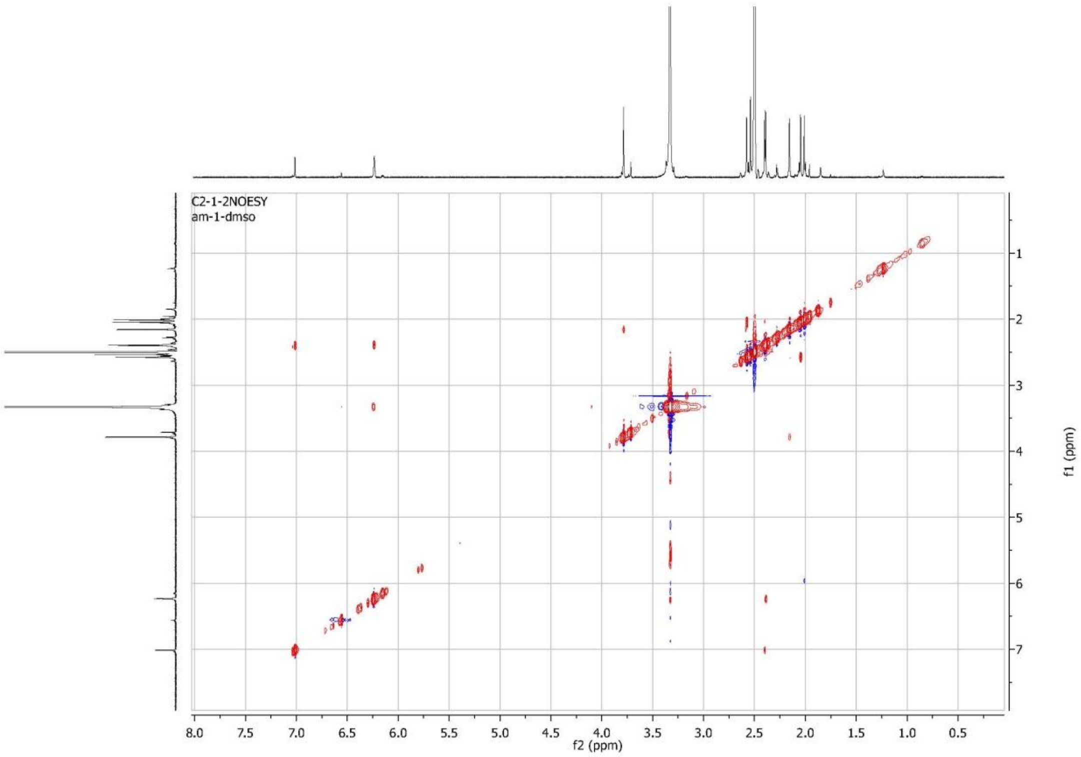

Figure S61 NOESY spectrum of **9** in DMSO-d_6

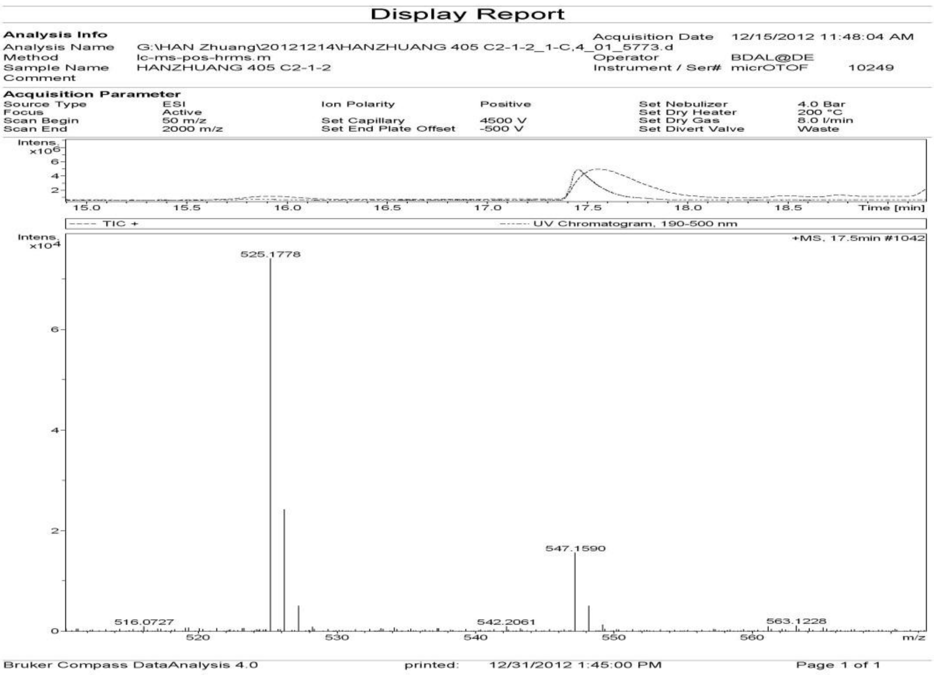

Figure S62 HRESIMS spectrum of **9**

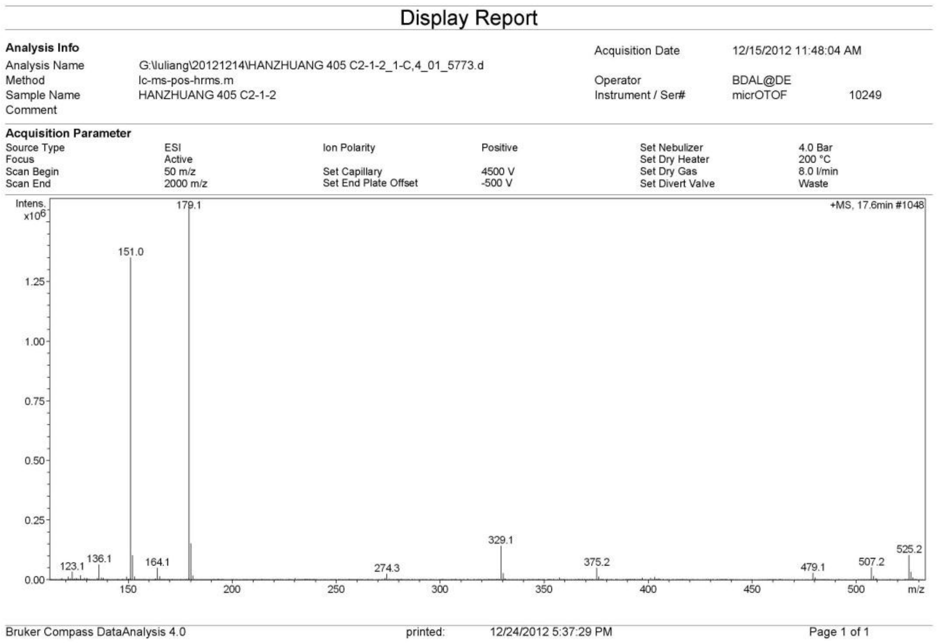

Figure S63 ISCID spectrum of **9**

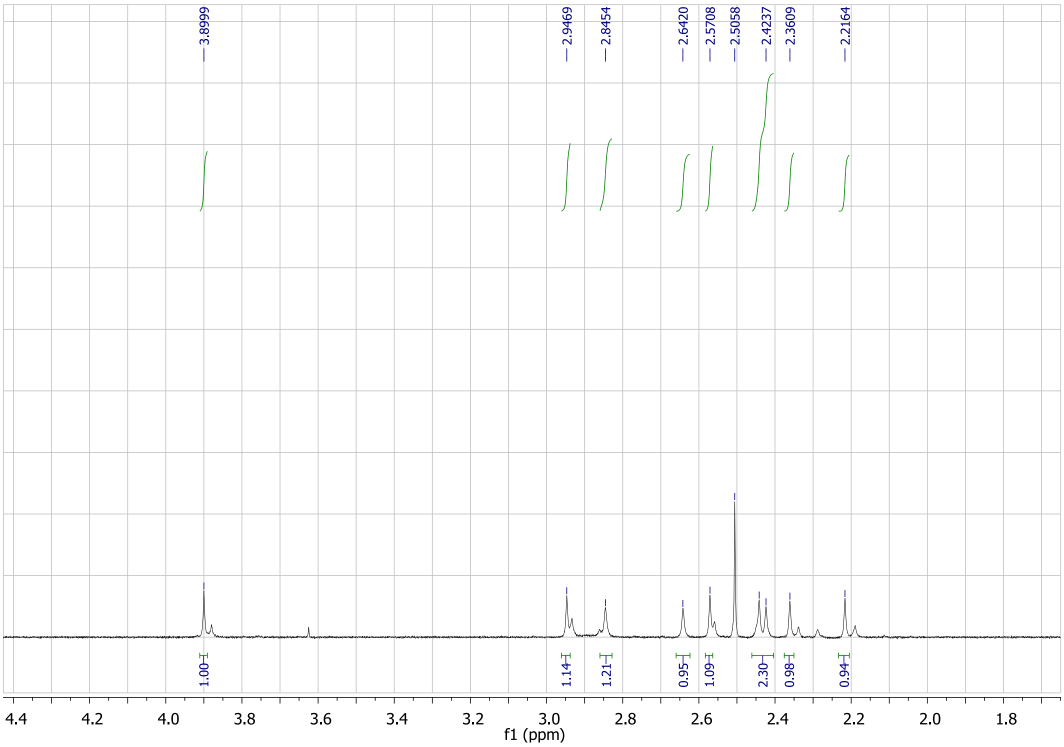

Figure S64 ^{1}H NMR spectrum of **10** in DMSO-d_6 (500 MHz)

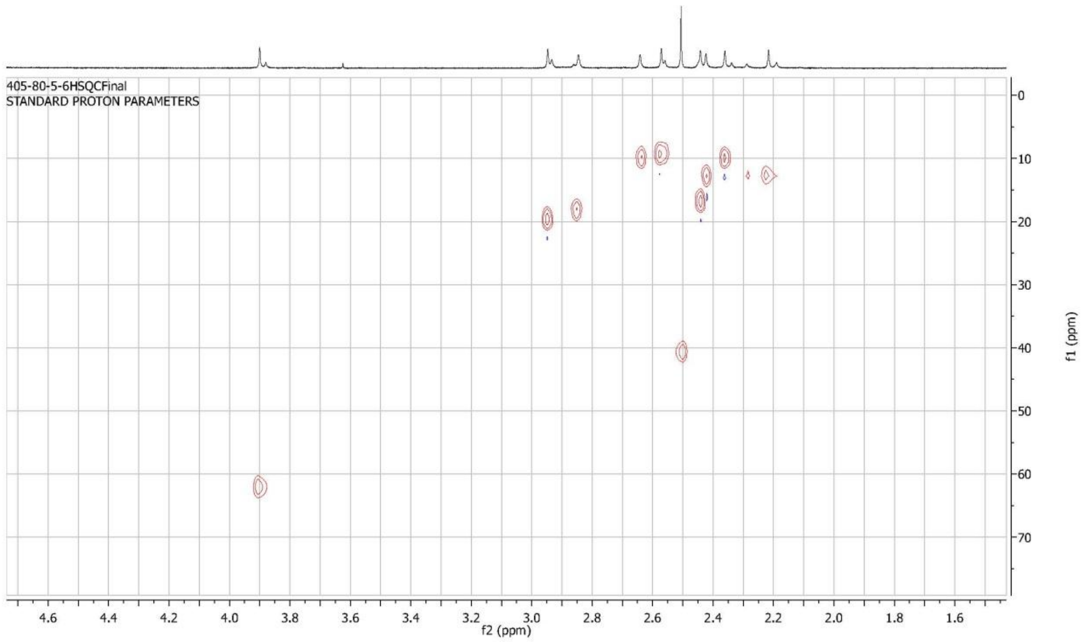

Figure S65 HMQC spectrum of **10** in DMSO-d_6

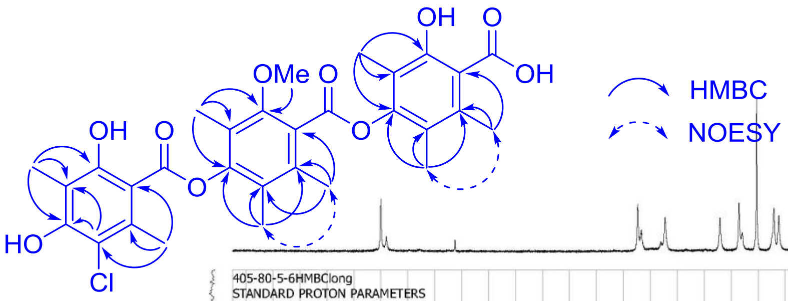

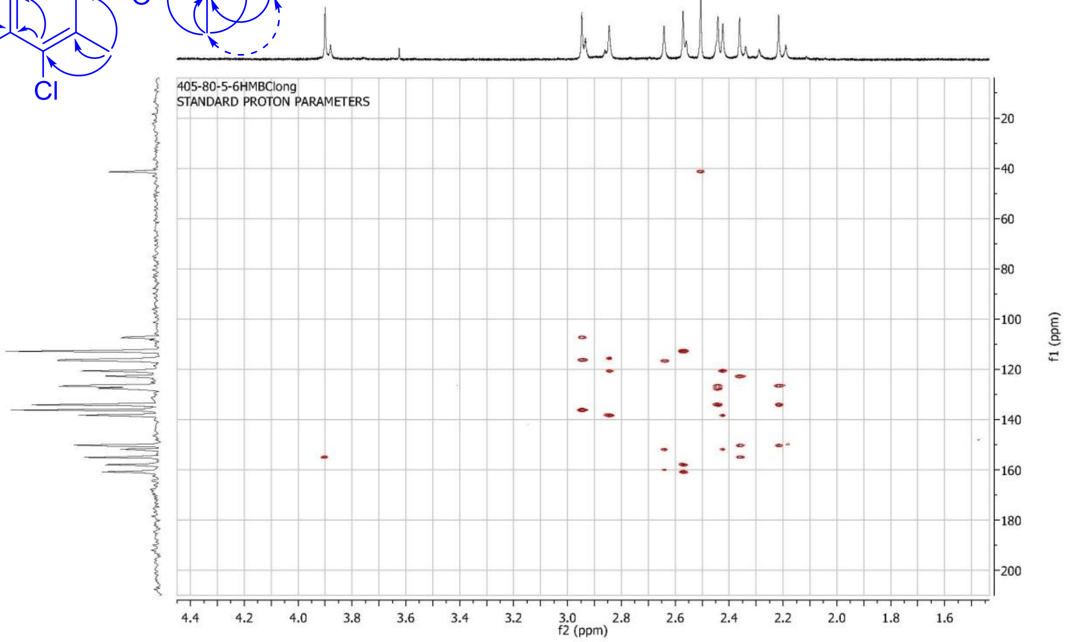

Figure S66 HMBC spectrum of **10** in DMSO-d_6

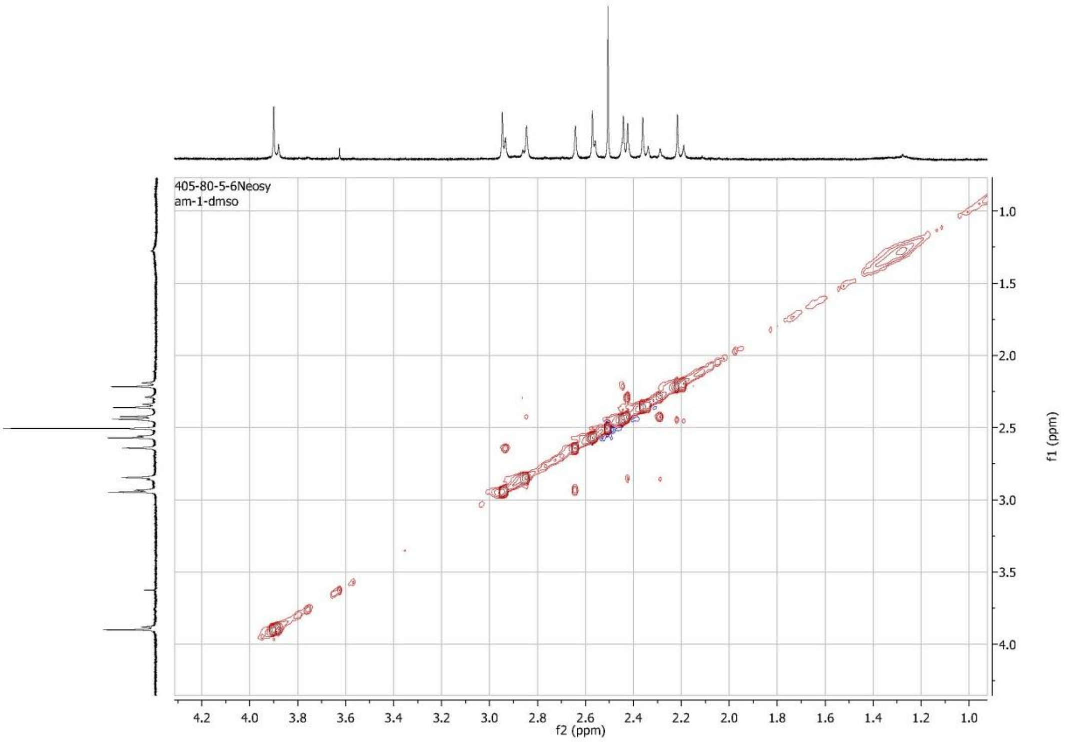

Figure S67 NOESY spectrum of **10** in DMSO-d_6

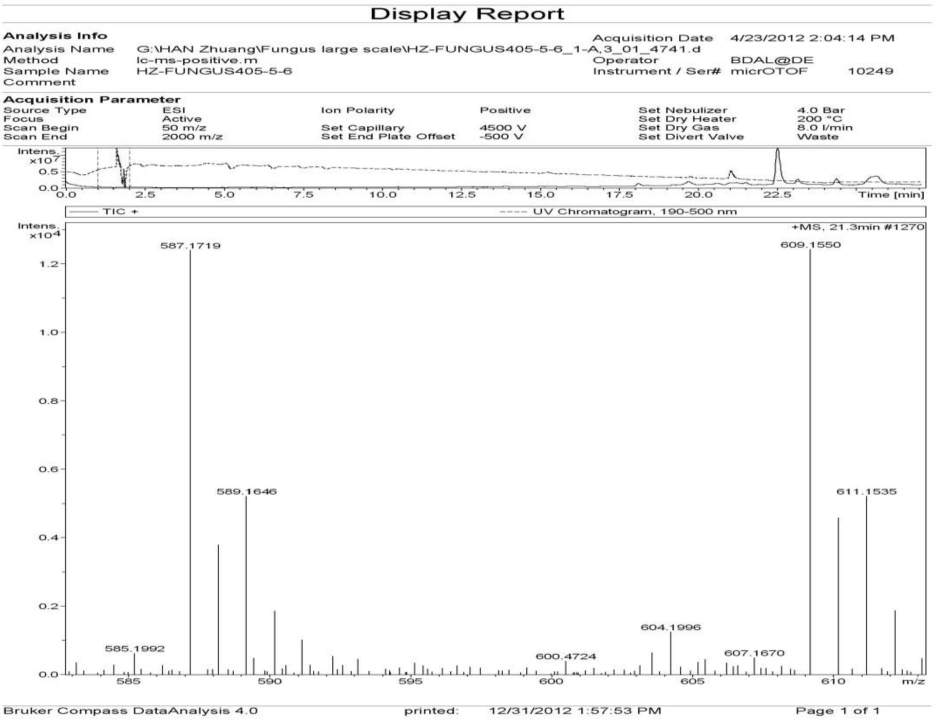

Figure S68 HRESIMS spectrum of **10**

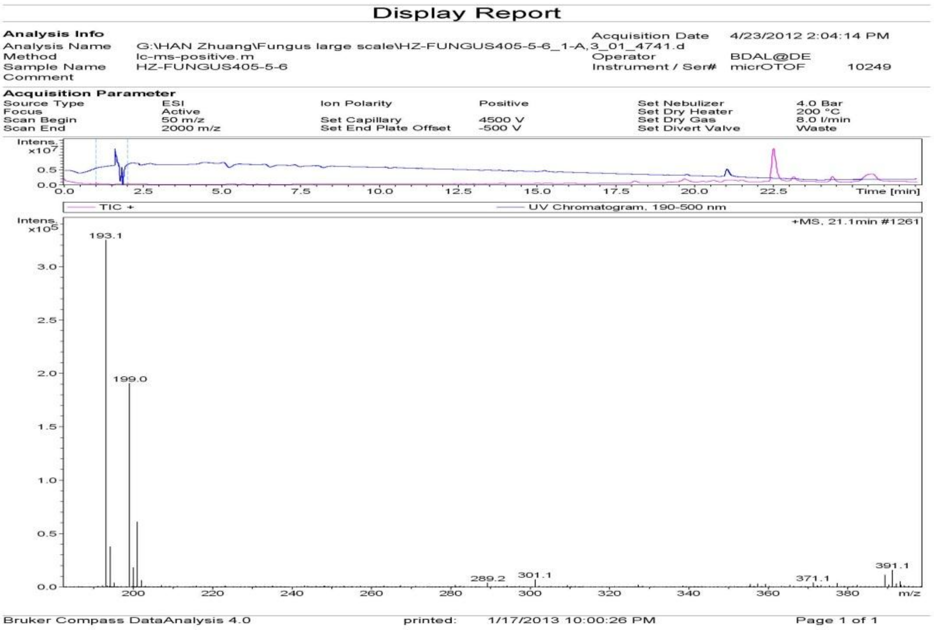

Figure S69A ISCID spectra of **10**

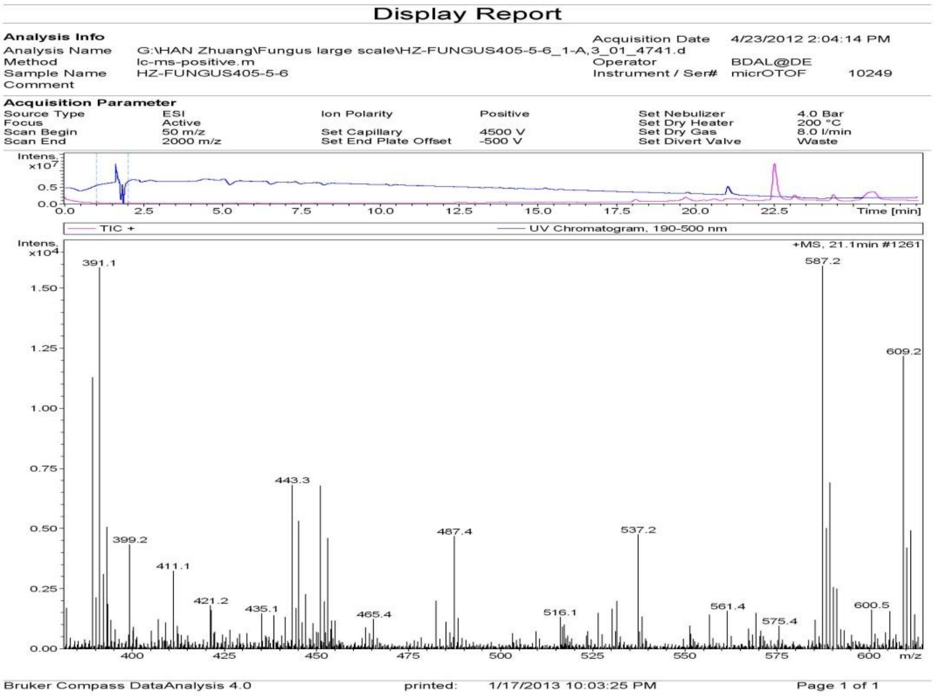

Figure S69B ISCID spectra of **10**

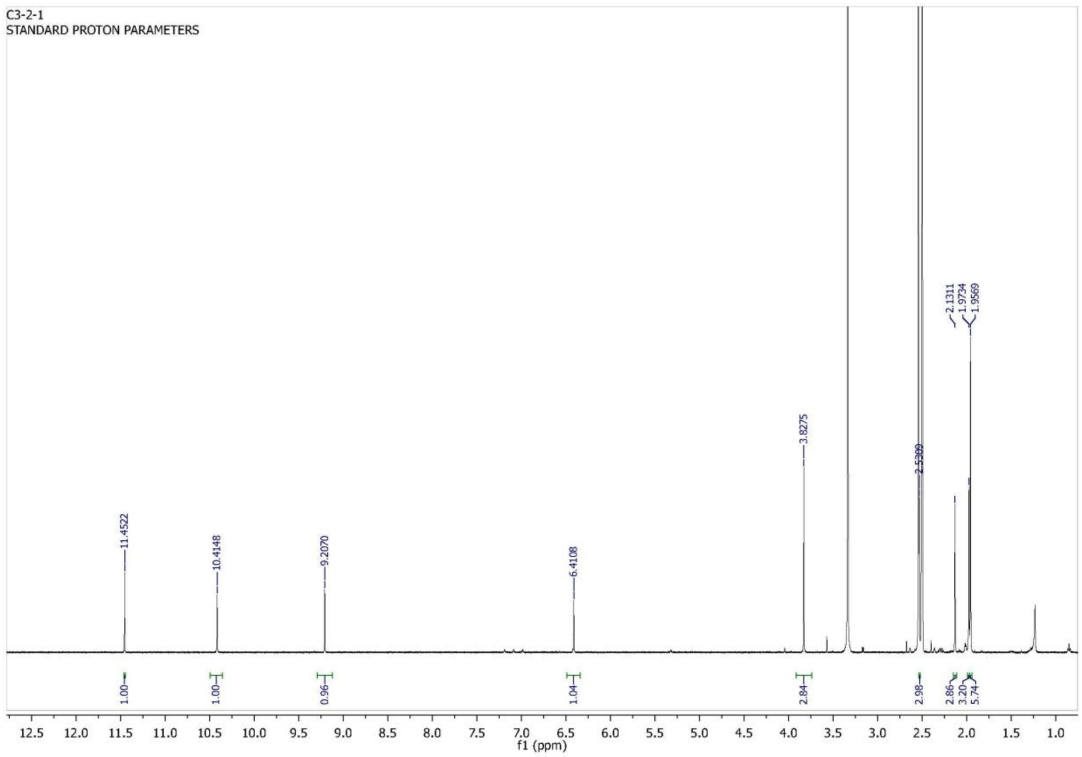

Figure S70 ^{1}H NMR spectrum of **11** in DMSO-d_6 (500 MHz)

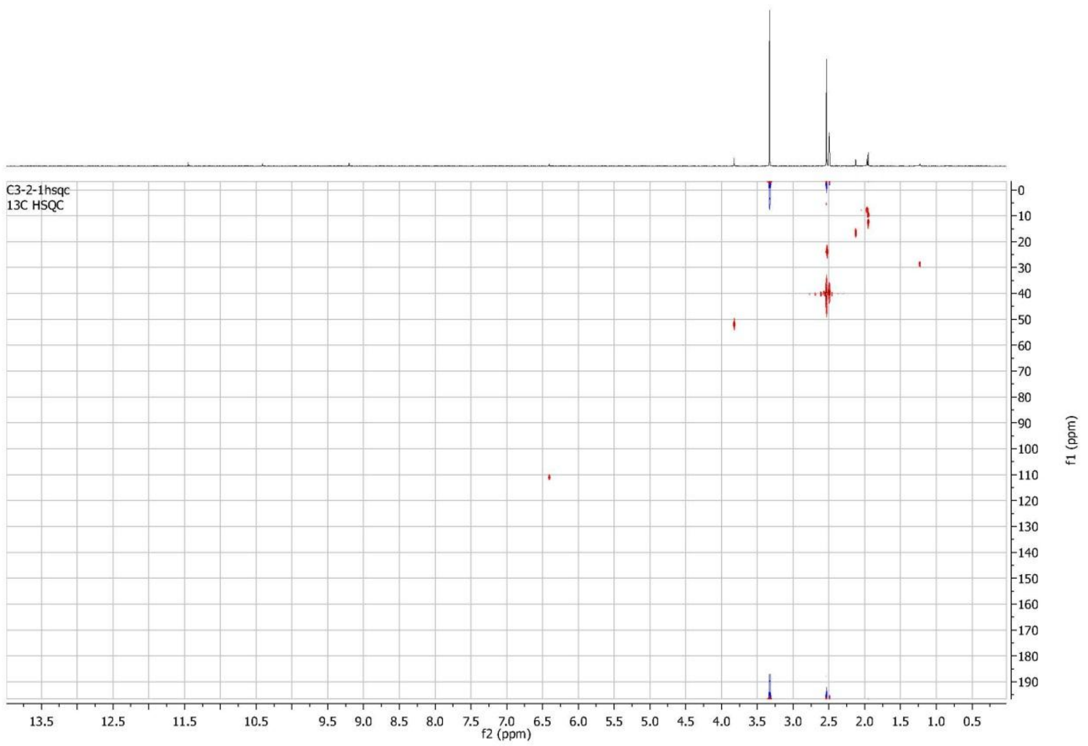

Figure S71 HMQC spectrum of **11** in DMSO-d_6

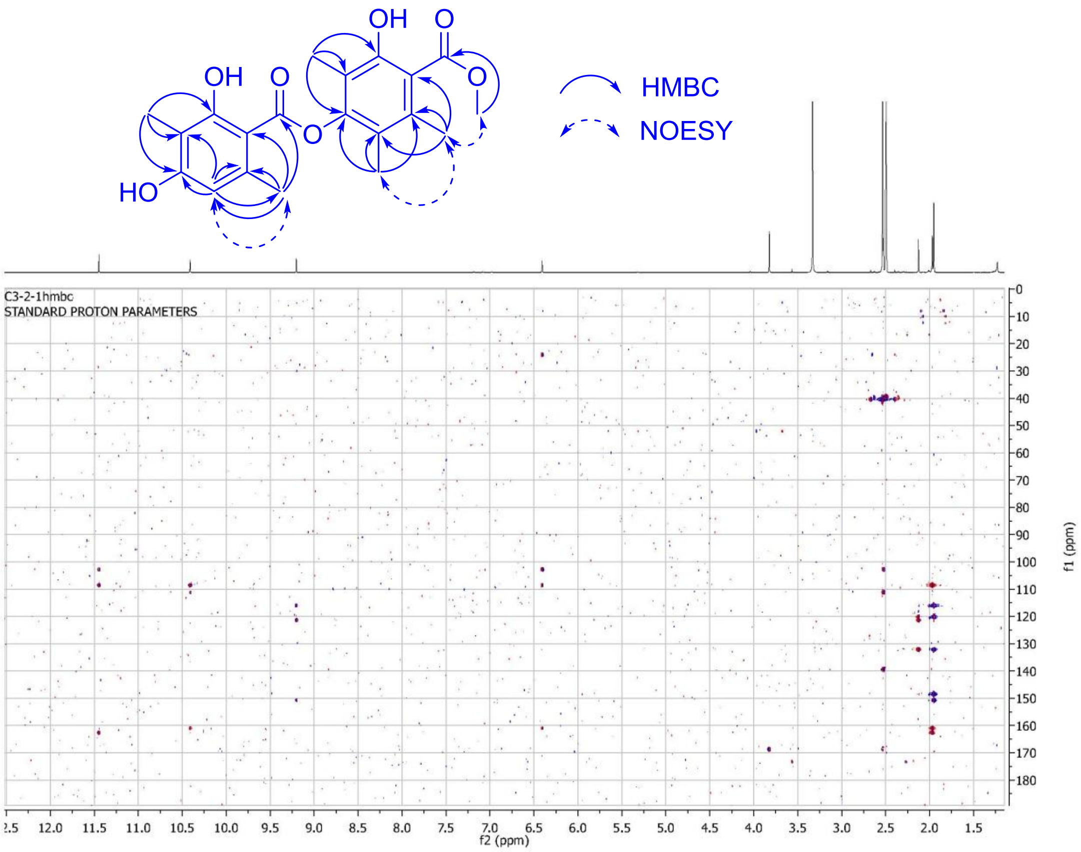

Figure S72 HMBC spectrum of **11** in DMSO-d_6

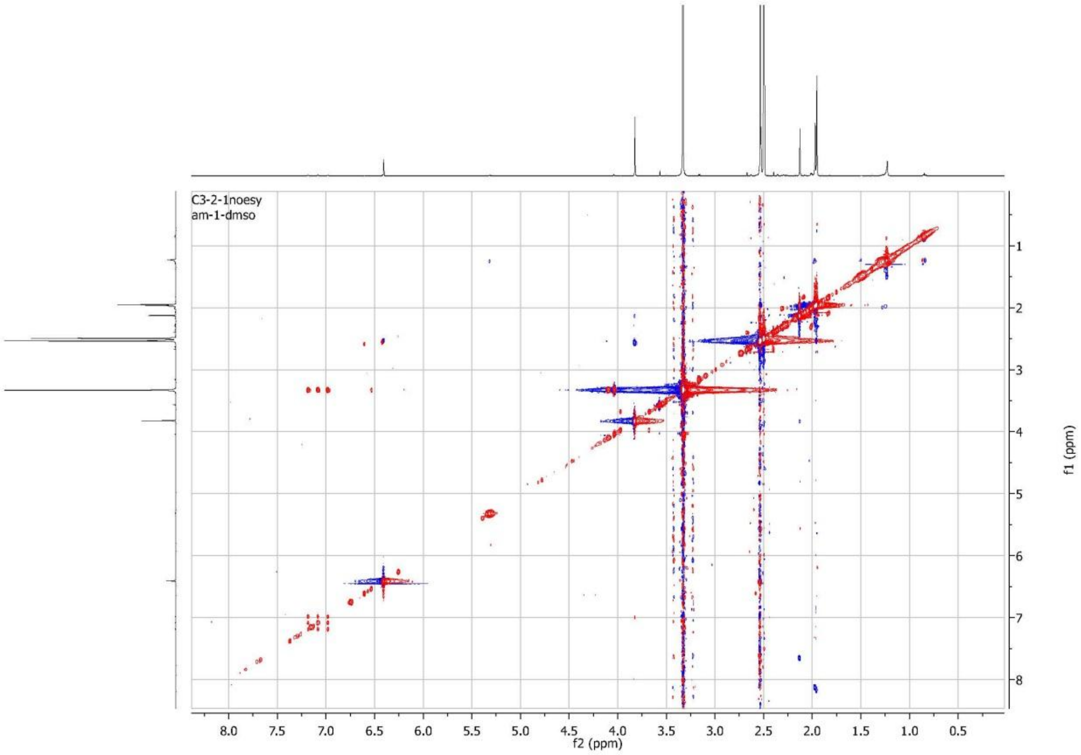

Figure S73 NOESY spectrum of **11** in DMSO-d_6

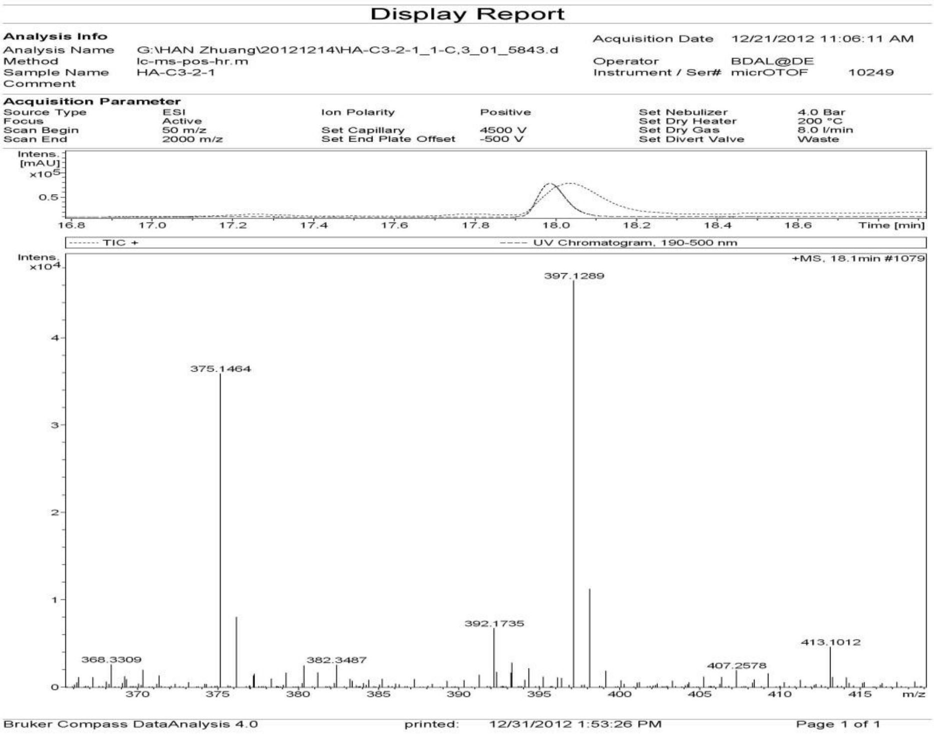

Figure S74 HRESIMS spectrum of **11**

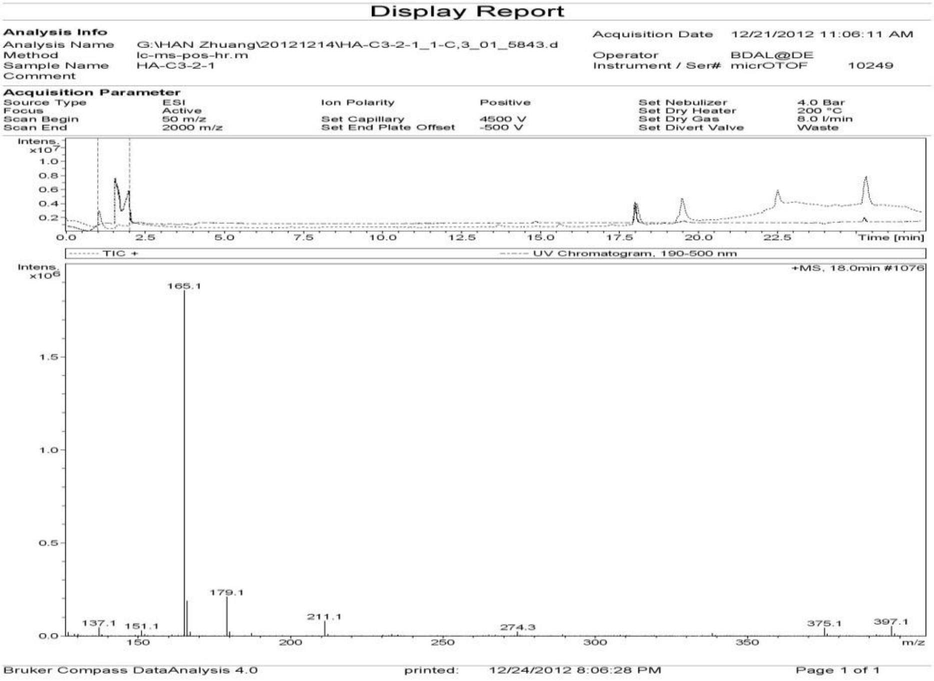

Figure S75 ISCID spectrum of **11**
